# Supplementary material for: Molecular surveillance of multiplicity of infection, haplotype frequencies, and prevalence in infectious diseases
Source: PLoS Comput Biol. 2026 Jul 17;22(7):e1012955. doi: 10.1371/journal.pcbi.1012955 (PMC13485136; doi:10.1371/journal.pcbi.1012955)
Supplement: Supplementary file 1 [file pcbi.1012955.s001.pdf]

Mathematical Appendix

Deriving the EM-algorithm

The derivation of the EM-algorithm consists of the E-step followed by the M-step. This derivation generalizes the ones in [1] for n biallelic loci and [2] for two multiallelic loci to the case of n multiallelic loci. Namely, it turns out that the structure of the derivations is almost identical and only needs slight modifications. For the sake of transparency, the presentation follows the ones in [1] and [2].

Expectation (E)-step

Each sample $\boldsymbol{x}^{(j)}$ in the dataset $\mathscr{X} = (\boldsymbol{x}^{(1)}, \ldots, \boldsymbol{x}^{(N)})$ results from a specific, but unobservable, MOI vector $\boldsymbol{m}^{(j)}$. Given sample size N, the unobservable MOI vectors underlying the observations $\mathscr{X}$ is denoted by $\mathscr{M} = (\boldsymbol{m}^{(1)}, \ldots, \boldsymbol{m}^{(N)})$. Hence, the likelihood function $L_{\mathscr{X},\mathscr{M}}$ of the unobservable data $\mathscr{M}$ and its derived observable data $\mathscr{X}$ is

$$L_{\mathscr{X},\mathscr{M}}(\boldsymbol{\theta}) \coloneqq \prod_{j=1}^{N} P\big(\boldsymbol{x}^{(j)}, \boldsymbol{m}^{(j)} \big| \boldsymbol{\theta}\big). \tag{A.1}$$

Let $\boldsymbol{\theta}_t$, denote the parameter choice in step t of the EM-algorithm. The Q-function is defined as the expectation of the log-likelihood function of the unknown parameter $\boldsymbol{\theta}$ (regarded as variables), with respect to the distribution of the unobserved data $\mathscr{M}$, conditioned on the observations $\mathscr{X}$ and the parameter choice $\boldsymbol{\theta}_t$. Formally,

$$\begin{aligned} Q(\boldsymbol{\theta} \,|\, \boldsymbol{\theta}_t) \coloneqq & \mathbb{E}_{\mathscr{M}|\mathscr{X},\boldsymbol{\theta}_t}[\log L_{\mathscr{X},\mathscr{M}}(\boldsymbol{\theta})] \\ = & \mathbb{E}_{\mathscr{M}|\mathscr{X},\boldsymbol{\theta}_t}\left[\sum_{j=1}^{N} \log P\big(\boldsymbol{x}^{(j)}, \boldsymbol{m}^{(j)} \big| \boldsymbol{\theta}\big)\right] \\ = & \sum_{j=1}^{N} \mathbb{E}_{\mathscr{M}|\mathscr{X},\boldsymbol{\theta}_t}\left[\log P\big(\boldsymbol{x}^{(j)}, \boldsymbol{m}^{(j)} \big| \boldsymbol{\theta}\big)\right]. \end{aligned}$$

Note that

$$\begin{aligned} &\mathbb{E}_{\mathscr{M}|\mathscr{X},\boldsymbol{\theta}_t} \log\left[P\big(\boldsymbol{x}^{(j)}, \boldsymbol{m}^{(j)} \big| \boldsymbol{\theta}\big)\right] \\ &\quad = \sum_{\boldsymbol{m}^{(1)},\ldots,\boldsymbol{m}^{(N)}} \log P\big(\boldsymbol{x}^{(j)}, \boldsymbol{m}^{(j)} \big| \boldsymbol{\theta}\big) P\big(\boldsymbol{m}^{(1)}, \ldots, \boldsymbol{m}^{(N)} \big| \boldsymbol{x}^{(1)}, \ldots, \boldsymbol{x}^{(N)}, \boldsymbol{\theta}_t\big) \\ &\quad = \sum_{\boldsymbol{m}^{(1)},\ldots,\boldsymbol{m}^{(N)}} \log P\big(\boldsymbol{x}^{(j)}, \boldsymbol{m}^{(j)} \big| \boldsymbol{\theta}\big) \frac{P\Big(\big(\boldsymbol{x}^{(1)}, \boldsymbol{m}^{(1)}\big), \ldots, \big(\boldsymbol{x}^{(N)}, \boldsymbol{m}^{(N)}\big) | \boldsymbol{\theta}_t\Big)}{P\big(\boldsymbol{x}^{(1)}, \ldots, \boldsymbol{x}^{(N)} | \boldsymbol{\theta}_t\big)}. \end{aligned}$$

Because samples are independent, one can rewrite the above as

$$\mathbb{E}_{\mathscr{M}|\mathscr{X},\boldsymbol{\theta}_t} \log\left[P\big(\boldsymbol{x}^{(j)}, \boldsymbol{m}^{(j)} \big| \boldsymbol{\theta}\big)\right] = \sum_{\boldsymbol{m}^{(1)},\ldots,\boldsymbol{m}^{(N)}} \log P\big(\boldsymbol{x}^{(j)}, \boldsymbol{m}^{(j)} \big| \boldsymbol{\theta}\big) \prod_{l=1}^{N} \frac{P\big(\boldsymbol{x}^{(l)}, \boldsymbol{m}^{(l)} | \boldsymbol{\theta}_t\big)}{P\big(\boldsymbol{x}^{(l)} \big| \boldsymbol{\theta}_t\big)}. \tag{A.2}$$

The factors in the product above can be rewritten as

$$\frac{P\big(\boldsymbol{x}^{(l)},\boldsymbol{m}^{(l)}\big|\boldsymbol{\theta}_t\big)}{P\big(\boldsymbol{x}^{(l)}\big|\boldsymbol{\theta}_t\big)}=\frac{P\big(\boldsymbol{x}^{(l)},\boldsymbol{m}^{(l)},\boldsymbol{\theta}_t\big)P\big(\boldsymbol{\theta}_t\big)}{P\big(\boldsymbol{x}^{(l)},\boldsymbol{\theta}_t\big)P\big(\boldsymbol{\theta}_t\big)}=\frac{P\big(\boldsymbol{x}^{(l)},\boldsymbol{m}^{(l)},\boldsymbol{\theta}_t\big)}{P\big(\boldsymbol{x}^{(l)},\boldsymbol{\theta}_t\big)}=P\big(\boldsymbol{m}^{(l)}\big|\boldsymbol{x}^{(l)},\boldsymbol{\theta}_t\big).$$

Using the above and $\sum_{\boldsymbol{m}^{(l)}} P\big(\boldsymbol{m}^{(l)}\big|\boldsymbol{x}^{(l)},\boldsymbol{\theta}_t\big)=1$ applied to all $l\neq j$ in (A.2) gives

$$\begin{aligned}\mathbb{E}_{\mathscr{M}|\mathscr{X},\boldsymbol{\theta}_t}\big[\log P\big(\boldsymbol{x}^{(j)},\boldsymbol{m}^{(j)}\big|\boldsymbol{\theta}\big)\big] &= \sum_{\boldsymbol{m}^{(1)},\dots,\boldsymbol{m}^{(N)}}\log P\big(\boldsymbol{x}^{(j)},\boldsymbol{m}^{(j)}\big|\boldsymbol{\theta}\big)\prod_{l=1}^{N}P\big(\boldsymbol{m}^{(l)}\big|\boldsymbol{x}^{(l)},\boldsymbol{\theta}_t\big)\\ &=\sum_{\boldsymbol{m}^{(j)}}\log P\big(\boldsymbol{x}^{(j)},\boldsymbol{m}^{(j)}\big|\boldsymbol{\theta}\big)P\big(\boldsymbol{m}^{(j)}\big|\boldsymbol{x}^{(j)},\boldsymbol{\theta}_t\big)\sum_{\substack{\boldsymbol{m}^{(1)},\dots,\boldsymbol{m}^{(j-1)},\\ \boldsymbol{m}^{(j+1)},\dots,\boldsymbol{m}^{(N)}}}\prod_{\substack{l=1\\ l\neq j}}^{N}P\big(\boldsymbol{m}^{(l)}\big|\boldsymbol{x}^{(l)},\boldsymbol{\theta}_t\big)\\ &=\sum_{\boldsymbol{m}^{(j)}}\log P\big(\boldsymbol{x}^{(j)},\boldsymbol{m}^{(j)}\big|\boldsymbol{\theta}\big)P\big(\boldsymbol{m}^{(j)}\big|\boldsymbol{x}^{(j)},\boldsymbol{\theta}_t\big).\end{aligned} \tag{A.3}$$

From the last line, one recognizes

$$\mathbb{E}_{\mathscr{M}|\mathscr{X},\boldsymbol{\theta}_t}\big[\log P\big(\boldsymbol{x}^{(j)},\boldsymbol{m}^{(j)}\big|\boldsymbol{\theta}\big)\big]=\mathbb{E}_{\boldsymbol{m}^{(j)}|\boldsymbol{x}^{(j)},\boldsymbol{\theta}_t}\big[\log P\big(\boldsymbol{x}^{(j)},\boldsymbol{m}^{(j)}\big|\boldsymbol{\theta}\big)\big]. \tag{A.4}$$

Hence, the Q-function becomes

$$Q(\boldsymbol{\theta}\,|\,\boldsymbol{\theta}_t)=\sum_{j=1}^{N}\mathbb{E}_{\boldsymbol{m}^{(j)}|\boldsymbol{x}^{(j)},\boldsymbol{\theta}_t}\big[\log P\big(\boldsymbol{x}^{(j)},\boldsymbol{m}^{(j)}\big|\boldsymbol{\theta}\big)\big]. \tag{A.5}$$

An observation $\boldsymbol{x}$ might occur several times in the data. Let $n_{\boldsymbol{x}}$ be the number of times observation $\boldsymbol{x}$ occurs in the data $\mathscr{X}$. Using this notation, the Q-function can be rewritten as

$$Q(\boldsymbol{\theta}\,|\,\boldsymbol{\theta}_t)=\sum_{\boldsymbol{x}\in\mathscr{O}}n_{\boldsymbol{x}}\mathbb{E}_{\boldsymbol{m}|\boldsymbol{x},\boldsymbol{\theta}_t}\big[\log P(\boldsymbol{x},\boldsymbol{m}|\boldsymbol{\theta})\big]. \tag{A.6a}$$

By defining

$$Q_{\boldsymbol{x}}(\boldsymbol{\theta}\,|\,\boldsymbol{\theta}_t):=\mathbb{E}_{\boldsymbol{m}|\boldsymbol{x},\boldsymbol{\theta}_t}\big[\log P(\boldsymbol{x},\boldsymbol{m}|\boldsymbol{\theta})\big], \tag{A.6b}$$

the Q-function becomes

$$Q(\boldsymbol{\theta}\,|\,\boldsymbol{\theta}_t)=\sum_{\boldsymbol{x}\in\mathscr{O}}n_{\boldsymbol{x}}Q_{\boldsymbol{x}}(\boldsymbol{\theta}\,|\,\boldsymbol{\theta}_t). \tag{A.6c}$$

First, expression (A.6b) is manipulated. From (40) one obtains

$$Q_{\boldsymbol{x}}(\boldsymbol{\theta}\,|\,\boldsymbol{\theta}_t)=\mathbb{E}_{\boldsymbol{m}|\boldsymbol{x},\boldsymbol{\theta}_t}\big[\log P(\boldsymbol{x},\boldsymbol{m}|\boldsymbol{\theta})\big]=\sum_{m=1}^{\infty}\sum_{\boldsymbol{m}\in M_{\boldsymbol{x}}^{(m)}}P(\boldsymbol{m}|\boldsymbol{x},\boldsymbol{\theta}_t)\log P(\boldsymbol{x},\boldsymbol{m}|\boldsymbol{\theta}), \tag{A.7a}$$

where the second equation is satisfied because $P(\boldsymbol{m}|\boldsymbol{x},\boldsymbol{\theta}_t)=0$ if $\boldsymbol{m}\notin M_{\boldsymbol{x}}^{(m)}$ for some m (see eq. 40 for the definition of the set $M_{\boldsymbol{x}}^{(m)}$). Because, $P(\boldsymbol{m}|\boldsymbol{x},\boldsymbol{\theta}_t)=\dfrac{P(\boldsymbol{x},\boldsymbol{m}|\boldsymbol{\theta}_t)}{P(\boldsymbol{x}|\boldsymbol{\theta}_t)}$ (see

above), combination with (A.7a) yields

$$\begin{aligned} Q_{\boldsymbol{x}}(\boldsymbol{\theta}\,|\,\boldsymbol{\theta}_t) &= \sum_{m=1}^{\infty} \sum_{\boldsymbol{m}\in M_{\boldsymbol{x}}^{(m)}} \frac{P(\boldsymbol{x},\boldsymbol{m}|\boldsymbol{\theta}_t)}{P(\boldsymbol{x}|\boldsymbol{\theta}_t)} \log P(\boldsymbol{x},\boldsymbol{m}|\boldsymbol{\theta}) \\ &= \frac{1}{P(\boldsymbol{x}|\boldsymbol{\theta}_t)} \sum_{m=1}^{\infty} \sum_{\boldsymbol{m}\in M_{\boldsymbol{x}}^{(m)}} P(\boldsymbol{x},\boldsymbol{m}|\boldsymbol{\theta}_t) \log P(\boldsymbol{x},\boldsymbol{m}|\boldsymbol{\theta}). \end{aligned}$$

Because $P(\boldsymbol{x},\boldsymbol{m}|\boldsymbol{\theta}_t) = P(\boldsymbol{x}|\boldsymbol{m},\boldsymbol{\theta}_t)P(\boldsymbol{m}|\boldsymbol{\theta}_t)$,

$$Q_{\boldsymbol{x}}(\boldsymbol{\theta}\,|\,\boldsymbol{\theta}_t) = \frac{1}{P(\boldsymbol{x}|\boldsymbol{\theta}_t)} \sum_{m=1}^{\infty} \sum_{\boldsymbol{m}\in M_{\boldsymbol{x}}^{(m)}} P(\boldsymbol{x}|\boldsymbol{m},\boldsymbol{\theta}_t)P(\boldsymbol{m}|\boldsymbol{\theta}_t) \log \big[P(\boldsymbol{x}|\boldsymbol{m},\boldsymbol{\theta})P(\boldsymbol{m}|\boldsymbol{\theta})\big].$$

For $\boldsymbol{m}\in M_{\boldsymbol{x}}^{(m)}$, clearly $P(\boldsymbol{x}|\boldsymbol{m},\boldsymbol{\theta}_t) = 1$ holds, such that

$$Q_{\boldsymbol{x}}(\boldsymbol{\theta}\,|\,\boldsymbol{\theta}_t) = \frac{1}{P(\boldsymbol{x}|\boldsymbol{\theta}_t)} \sum_{m=1}^{\infty} \sum_{\boldsymbol{m}\in M_{\boldsymbol{x}}^{(m)}} P(\boldsymbol{m}|\boldsymbol{\theta}_t) \log P(\boldsymbol{m}|\boldsymbol{\theta}). \tag{A.7b}$$

Furthermore, $P(\boldsymbol{m}|\boldsymbol{\theta}_t) = P(\boldsymbol{m}|m,\boldsymbol{\theta}_t)P(m|\boldsymbol{\theta}_t)$ for $m = |\boldsymbol{m}|$. Let $\kappa_m^{(t)} := P(m|\boldsymbol{\theta}_t)$ in analogy to $\kappa_m = P(m|\boldsymbol{\theta})$, so that (A.7b) becomes

$$\begin{aligned} Q_{\boldsymbol{x}}(\boldsymbol{\theta}\,|\,\boldsymbol{\theta}_t) &= \frac{1}{P(\boldsymbol{x}|\boldsymbol{\theta}_t)} \sum_{m=1}^{\infty} \sum_{\boldsymbol{m}\in M_{\boldsymbol{x}}^{(m)}} P(\boldsymbol{m}|m,\boldsymbol{\theta}_t)P(m|\boldsymbol{\theta}_t) \log \big[P(\boldsymbol{m}|m,\boldsymbol{\theta})P(m|\boldsymbol{\theta})\big] \\ &= \frac{1}{P(\boldsymbol{x}|\boldsymbol{\theta}_t)} \sum_{m=1}^{\infty} \sum_{\boldsymbol{m}\in M_{\boldsymbol{x}}^{(m)}} \kappa_m^{(t)} \binom{m}{\boldsymbol{m}} \boldsymbol{p}_t^{\boldsymbol{m}} \log \left(\kappa_m \binom{m}{\boldsymbol{m}} \boldsymbol{p}^{\boldsymbol{m}}\right). \end{aligned} \tag{A.7c}$$

The inclusion-exclusion principle as used in (42) leads to

$$Q_{\boldsymbol{x}}(\boldsymbol{\theta}\,|\,\boldsymbol{\theta}_t) = \frac{1}{P(\boldsymbol{x}|\boldsymbol{\theta}_t)} \sum_{m=1}^{\infty} \kappa_m^{(t)} \sum_{\boldsymbol{y}\in\mathscr{A}_{\boldsymbol{x}}} (-1)^{|\boldsymbol{x}|-|\boldsymbol{y}|} \sum_{\substack{\boldsymbol{m}:|\boldsymbol{m}|=m \\ m_{\boldsymbol{h}}=0 \text{ if } \boldsymbol{h}\notin A_{\boldsymbol{y}}}} \binom{m}{\boldsymbol{m}} \boldsymbol{p}_t^{\boldsymbol{m}} \log \left(\kappa_m \binom{m}{\boldsymbol{m}} \boldsymbol{p}^{\boldsymbol{m}}\right), \tag{A.8a}$$

where $|\boldsymbol{x}|$ and $|\boldsymbol{y}|$ are the cardinality of the observations (sets) $\boldsymbol{x}$ and $\boldsymbol{y}$, respectively. Therefore, multiplication with $P(\boldsymbol{x}\,|\,\boldsymbol{\theta}_t)$ gives

$$\begin{aligned} &P(\boldsymbol{x}\,|\,\boldsymbol{\theta}_t) Q_{\boldsymbol{x}}(\boldsymbol{\theta}\,|\,\boldsymbol{\theta}_t) \\ &= \sum_{\boldsymbol{y}\in\mathscr{A}_{\boldsymbol{x}}} (-1)^{|\boldsymbol{x}|-|\boldsymbol{y}|} \sum_{m=1}^{\infty} \kappa_m^{(t)} \sum_{\substack{\boldsymbol{m}:|\boldsymbol{m}|=m \\ m_{\boldsymbol{h}}=0 \text{ if } \boldsymbol{h}\notin A_{\boldsymbol{y}}}} \binom{m}{\boldsymbol{m}} \boldsymbol{p}_t^{\boldsymbol{m}} \log \left(\kappa_m \binom{m}{\boldsymbol{m}} \boldsymbol{p}^{\boldsymbol{m}}\right) \\ &= \sum_{\boldsymbol{y}\in\mathscr{A}_{\boldsymbol{x}}} (-1)^{|\boldsymbol{x}|-|\boldsymbol{y}|} \sum_{m=1}^{\infty} \kappa_m^{(t)} \sum_{\substack{\boldsymbol{m}:|\boldsymbol{m}|=m \\ m_{\boldsymbol{h}}=0 \text{ if } \boldsymbol{h}\notin A_{\boldsymbol{y}}}} \binom{m}{\boldsymbol{m}} \boldsymbol{p}_t^{\boldsymbol{m}} \left(\log \kappa_m + \log \binom{m}{\boldsymbol{m}} + \log \boldsymbol{p}^{\boldsymbol{m}}\right) \\ &= \sum_{\boldsymbol{y}\in\mathscr{A}_{\boldsymbol{x}}} (-1)^{|\boldsymbol{x}|-|\boldsymbol{y}|} \sum_{m=1}^{\infty} \kappa_m^{(t)} \sum_{\substack{\boldsymbol{m}:|\boldsymbol{m}|=m \\ m_{\boldsymbol{h}}=0 \text{ if } \boldsymbol{h}\notin A_{\boldsymbol{y}}}} \binom{m}{\boldsymbol{m}} \boldsymbol{p}_t^{\boldsymbol{m}} \left(\log \left(\frac{1}{e^{\lambda}-1}\frac{\lambda^m}{m!}\right) + \log \binom{m}{\boldsymbol{m}} + \log \boldsymbol{p}^{\boldsymbol{m}}\right) \\ &= \sum_{\boldsymbol{y}\in\mathscr{A}_{\boldsymbol{x}}} (-1)^{|\boldsymbol{x}|-|\boldsymbol{y}|} \sum_{m=1}^{\infty} \kappa_m^{(t)} \sum_{\substack{\boldsymbol{m}:|\boldsymbol{m}|=m \\ m_{\boldsymbol{h}}=0 \text{ if } \boldsymbol{h}\notin A_{\boldsymbol{y}}}} \binom{m}{\boldsymbol{m}} \boldsymbol{p}_t^{\boldsymbol{m}} \left(-\log(e^{\lambda}-1) + m\log\lambda + \log \boldsymbol{p}^{\boldsymbol{m}}\right) + \tilde{A}_t, \end{aligned}$$

where

$$\tilde{A}_t := \sum_{\boldsymbol{y}\in\mathscr{A}_{\boldsymbol{x}}} (-1)^{|\boldsymbol{x}|-|\boldsymbol{y}|} \sum_{m=1}^{\infty} \kappa_m^{(t)} \sum_{\substack{\boldsymbol{m}:|\boldsymbol{m}|=m\\ m_{\boldsymbol{h}}=0 \text{ if } \boldsymbol{h}\notin A_{\boldsymbol{y}}}} \binom{m}{\boldsymbol{m}} \boldsymbol{p}_t^{\boldsymbol{m}} \left(\log \binom{m}{\boldsymbol{m}} - \log(m!) \right),$$

is independent of $\boldsymbol{\theta}$. Hence,

$$\begin{aligned} P(\boldsymbol{x}\,|\,\boldsymbol{\theta}_t)Q_{\boldsymbol{x}}(\boldsymbol{\theta}\,|\,\boldsymbol{\theta}_t) =& -\log(e^{\lambda}-1) \sum_{\boldsymbol{y}\in\mathscr{A}_{\boldsymbol{x}}} (-1)^{|\boldsymbol{x}|-|\boldsymbol{y}|} \sum_{m=1}^{\infty} \kappa_m^{(t)} \sum_{\substack{\boldsymbol{m}:|\boldsymbol{m}|=m\\ m_{\boldsymbol{h}}=0 \text{ if } \boldsymbol{h}\notin A_{\boldsymbol{y}}}} \binom{m}{\boldsymbol{m}} \boldsymbol{p}_t^{\boldsymbol{m}} \\ &+ \log\lambda \sum_{\boldsymbol{y}\in\mathscr{A}_{\boldsymbol{x}}} (-1)^{|\boldsymbol{x}|-|\boldsymbol{y}|} \sum_{m=1}^{\infty} m\kappa_m^{(t)} \sum_{\substack{\boldsymbol{m}:|\boldsymbol{m}|=m\\ m_{\boldsymbol{h}}=0 \text{ if } \boldsymbol{h}\notin A_{\boldsymbol{y}}}} \binom{m}{\boldsymbol{m}} \boldsymbol{p}_t^{\boldsymbol{m}} \\ &+ \sum_{\boldsymbol{y}\in\mathscr{A}_{\boldsymbol{x}}} (-1)^{|\boldsymbol{x}|-|\boldsymbol{y}|} \sum_{m=1}^{\infty} \kappa_m^{(t)} \sum_{\boldsymbol{h}\in\mathscr{H}} \log p_{\boldsymbol{h}} \sum_{\substack{\boldsymbol{m}:|\boldsymbol{m}|=m\\ m_{\boldsymbol{i}}=0 \text{ if } \boldsymbol{i}\notin A_{\boldsymbol{y}}}} m_{\boldsymbol{h}} \binom{m}{\boldsymbol{m}} \boldsymbol{p}_t^{\boldsymbol{m}} \\ &+ \tilde{A}_t. \end{aligned} \tag{A.8b}$$

From (46) and the multinomial theorem one obtains

$$\begin{aligned} P(\boldsymbol{x}\,|\,\boldsymbol{\theta}_t)Q_{\boldsymbol{x}}(\boldsymbol{\theta}\,|\,\boldsymbol{\theta}_t) =& -P(\boldsymbol{x}\,|\,\boldsymbol{\theta}_t)\log\left(e^{\lambda}-1\right) \\ &+ \log\lambda \sum_{\boldsymbol{y}\in\mathscr{A}_{\boldsymbol{x}}} (-1)^{|\boldsymbol{x}|-|\boldsymbol{y}|} \sum_{m=1}^{\infty} m\kappa_m^{(t)} \Big(\sum_{\boldsymbol{i}\in A_{\boldsymbol{y}}} p_{\boldsymbol{i}}^{(t)} \Big)^m \\ &+ \sum_{\boldsymbol{y}\in\mathscr{A}_{\boldsymbol{x}}} (-1)^{|\boldsymbol{x}|-|\boldsymbol{y}|} \sum_{m=1}^{\infty} \kappa_m^{(t)} \sum_{\boldsymbol{h}\in\mathscr{H}} \log p_{\boldsymbol{h}} \sum_{\substack{\boldsymbol{m}:|\boldsymbol{m}|=m\\ m_{\boldsymbol{i}}=0 \text{ if } \boldsymbol{i}\notin A_{\boldsymbol{y}}}} m_{\boldsymbol{h}} \binom{m}{\boldsymbol{m}} \boldsymbol{p}_t^{\boldsymbol{m}} \\ &+ \tilde{A}_t. \end{aligned} \tag{A.8c}$$

To simplify the last row above, the notation $\boldsymbol{m}_{-\boldsymbol{h}}$ is introduced, which is the MOI vector $\boldsymbol{m}$ with the component of haplotype $\boldsymbol{h}$ reduced by 1, i.e., $m_{\boldsymbol{h}}$ is replaced by $m_{\boldsymbol{h}}-1$. Therefore, by rearranging the binomial coefficient $\binom{m}{\boldsymbol{m}}$ one obtains

$$\begin{aligned} P(\boldsymbol{x}\,|\,\boldsymbol{\theta}_t)Q_{\boldsymbol{x}}(\boldsymbol{\theta}\,|\,\boldsymbol{\theta}_t) =& -P(\boldsymbol{x}\,|\,\boldsymbol{\theta}_t)\log\left(e^{\lambda}-1\right) \\ &+ \log\lambda \sum_{\boldsymbol{y}\in\mathscr{A}_{\boldsymbol{x}}} (-1)^{|\boldsymbol{x}|-|\boldsymbol{y}|} \sum_{m=1}^{\infty} m\kappa_m^{(t)} \Big(\sum_{\boldsymbol{i}\in A_{\boldsymbol{y}}} p_{\boldsymbol{i}}^{(t)} \Big)^m \\ &+ \sum_{\boldsymbol{y}\in\mathscr{A}_{\boldsymbol{x}}} (-1)^{|\boldsymbol{x}|-|\boldsymbol{y}|} \sum_{\boldsymbol{h}\in\mathscr{H}} p_{\boldsymbol{h}}^{(t)} \log p_{\boldsymbol{h}} \sum_{m=1}^{\infty} \kappa_m^{(t)} \sum_{\substack{\boldsymbol{m}:|\boldsymbol{m}|=m\\ m_{\boldsymbol{i}}=0 \text{ if } \boldsymbol{i}\notin A_{\boldsymbol{y}}\\ m_{\boldsymbol{h}}>0}} \binom{m-1}{\boldsymbol{m}_{-\boldsymbol{h}}} m \boldsymbol{p}_t^{\boldsymbol{m}_{-\boldsymbol{h}}} \\ &+ \tilde{A}_t. \end{aligned}$$

Rearrangement in the second line and using the multinomial theorem in the third line

(together with the fact that $\boldsymbol{h} \in A_{\boldsymbol{y}}$ in the last sum) yields

$$\begin{aligned}P(\boldsymbol{x}\,|\,\boldsymbol{\theta}_t)Q_{\boldsymbol{x}}(\boldsymbol{\theta}\,|\,\boldsymbol{\theta}_t) = &-P(\boldsymbol{x}\,|\,\boldsymbol{\theta}_t)\log\left(e^{\lambda}-1\right)\\ &+\log\lambda\sum_{\boldsymbol{y}\in\mathscr{A}_{\boldsymbol{x}}}(-1)^{|\boldsymbol{x}|-|\boldsymbol{y}|}\sum_{\boldsymbol{h}\in A_{\boldsymbol{y}}}p_{\boldsymbol{h}}^{(t)}\sum_{m=1}^{\infty}m\kappa_m^{(t)}\Big(\sum_{\boldsymbol{i}\in A_{\boldsymbol{y}}}p_{\boldsymbol{i}}^{(t)}\Big)^{m-1}\\ &+\sum_{\boldsymbol{y}\in\mathscr{A}_{\boldsymbol{x}}}(-1)^{|\boldsymbol{x}|-|\boldsymbol{y}|}\sum_{\boldsymbol{h}\in\mathscr{H}}p_{\boldsymbol{h}}^{(t)}\log p_{\boldsymbol{h}}\sum_{m=1}^{\infty}m\kappa_m^{(t)}\Big(\sum_{\boldsymbol{i}\in A_{\boldsymbol{y}}}p_{\boldsymbol{i}}^{(t)}\Big)^{m-1}\\ &+\tilde{A}_t.\end{aligned} \tag{A.8d}$$

Inside the sums in the second and third lines of the above equation one recognizes derivatives of the PGF. The fact that $\boldsymbol{h} \in A_{\boldsymbol{y}}$ and the definition of the PGF lead to

$$\begin{aligned}\sum_{m=1}^{\infty}m\kappa_m^{(t)}\Big(\sum_{\boldsymbol{i}\in A_{\boldsymbol{y}}}p_{\boldsymbol{i}}^{(t)}\Big)^{m-1} &= \sum_{m=1}^{\infty}\kappa_m^{(t)}\frac{\partial}{\partial p_{\boldsymbol{h}}^{(t)}}\Big(\sum_{\boldsymbol{i}\in A_{\boldsymbol{y}}}p_{\boldsymbol{i}}^{(t)}\Big)^{m}\\ &= \frac{\partial}{\partial p_{\boldsymbol{h}}^{(t)}}\sum_{m=1}^{\infty}\kappa_m^{(t)}\Big(\sum_{\boldsymbol{i}\in A_{\boldsymbol{y}}}p_{\boldsymbol{i}}^{(t)}\Big)^{m}\\ &= \frac{\partial}{\partial p_{\boldsymbol{h}}^{(t)}}G\Big(\sum_{\boldsymbol{i}\in A_{\boldsymbol{y}}}p_{\boldsymbol{i}}^{(t)}\Big).\end{aligned} \tag{A.8e}$$

Combination of (A.8e) and (A.8d) gives

$$\begin{aligned}P(\boldsymbol{x}\,|\,\boldsymbol{\theta}_t)Q_{\boldsymbol{x}}(\boldsymbol{\theta}\,|\,\boldsymbol{\theta}_t) = &-P(\boldsymbol{x}\,|\,\boldsymbol{\theta}_t)\log\left(e^{\lambda}-1\right)\\ &+\log\lambda\sum_{\boldsymbol{y}\in\mathscr{A}_{\boldsymbol{x}}}(-1)^{|\boldsymbol{x}|-|\boldsymbol{y}|}\sum_{\boldsymbol{h}\in A_{\boldsymbol{y}}}p_{\boldsymbol{h}}^{(t)}\frac{\partial}{\partial p_{\boldsymbol{h}}^{(t)}}G_t\Big(\sum_{\boldsymbol{i}\in A_{\boldsymbol{y}}}p_{\boldsymbol{i}}^{(t)}\Big)\\ &+\sum_{\boldsymbol{y}\in\mathscr{A}_{\boldsymbol{x}}}(-1)^{|\boldsymbol{x}|-|\boldsymbol{y}|}\sum_{\boldsymbol{h}\in\mathscr{H}}p_{\boldsymbol{h}}^{(t)}\log p_{\boldsymbol{h}}\frac{\partial}{\partial p_{\boldsymbol{h}}^{(t)}}G_t\Big(\sum_{\boldsymbol{i}\in A_{\boldsymbol{y}}}p_{\boldsymbol{i}}^{(t)}\Big)\\ &+\tilde{A}_t,\end{aligned} \tag{A.8f}$$

where the subscript t in G_t emphasizes the dependency of the PGF G on λ_t in iteration t. Therefore,

$$\begin{aligned}Q_{\boldsymbol{x}}(\boldsymbol{\theta}\,|\,\boldsymbol{\theta}_t) = &-\log\left(e^{\lambda}-1\right)\\ &+\log\lambda\frac{\sum_{\boldsymbol{y}\in\mathscr{A}_{\boldsymbol{x}}}(-1)^{|\boldsymbol{x}|-|\boldsymbol{y}|}\sum_{\boldsymbol{h}\in A_{\boldsymbol{y}}}p_{\boldsymbol{h}}^{(t)}\frac{\partial}{\partial p_{\boldsymbol{h}}^{(t)}}G_t\Big(\sum_{\boldsymbol{i}\in A_{\boldsymbol{y}}}p_{\boldsymbol{i}}^{(t)}\Big)}{P(\boldsymbol{x}\,|\,\boldsymbol{\theta}_t)}\\ &+\frac{\sum_{\boldsymbol{y}\in\mathscr{A}_{\boldsymbol{x}}}(-1)^{|\boldsymbol{x}|-|\boldsymbol{y}|}\sum_{\boldsymbol{h}\in\mathscr{H}}p_{\boldsymbol{h}}^{(t)}\log p_{\boldsymbol{h}}\frac{\partial}{\partial p_{\boldsymbol{h}}^{(t)}}G_t\Big(\sum_{\boldsymbol{i}\in A_{\boldsymbol{y}}}p_{\boldsymbol{i}}^{(t)}\Big)}{P(\boldsymbol{x}\,|\,\boldsymbol{\theta}_t)}\\ &+\tilde{\tilde{A}}_t,\end{aligned} \tag{A.9a}$$

where $\tilde{\tilde{A}}_t = \dfrac{\tilde{A}_t}{P(\boldsymbol{x}\,|\,\boldsymbol{\theta}_t)}$. Note that

$$\frac{\partial}{\partial p_{\boldsymbol{h}}^{(t)}}G_t\Big(\sum_{\boldsymbol{i}\in A_{\boldsymbol{y}}}p_{\boldsymbol{i}}^{(t)}\Big) = G_t'\Big(\sum_{\boldsymbol{i}\in A_{\boldsymbol{y}}}p_{\boldsymbol{i}}^{(t)}\Big)I_{A_{\boldsymbol{y}}}(\boldsymbol{h}), \tag{A.9b}$$

where

$$I_{A_{\boldsymbol{y}}}(\boldsymbol{h}) = \begin{cases} 1 & \text{if } \boldsymbol{h} \in A_{\boldsymbol{y}}, \\ 0 & \text{if } \boldsymbol{h} \notin A_{\boldsymbol{y}}. \end{cases} \tag{A.9c}$$

Using this fact and the definition of $P(\boldsymbol{x} \,|\, \boldsymbol{\theta}_t)$ in (46) yields,

$$\begin{aligned} Q_{\boldsymbol{x}}(\boldsymbol{\theta} \,|\, \boldsymbol{\theta}_t) = &-\log(e^{\lambda} - 1) \\ &+ \log \lambda \frac{\sum_{\boldsymbol{y} \in \mathscr{A}_{\boldsymbol{x}}} (-1)^{|\boldsymbol{x}|-|\boldsymbol{y}|} \sum_{\boldsymbol{h} \in A_{\boldsymbol{y}}} p_{\boldsymbol{h}}^{(t)} G_t' \Big(\sum_{\boldsymbol{i} \in A_{\boldsymbol{y}}} p_{\boldsymbol{i}}^{(t)} \Big)}{\sum_{\boldsymbol{y} \in \mathscr{A}_{\boldsymbol{x}}} (-1)^{|\boldsymbol{x}|-|\boldsymbol{y}|} G_t \big(\sum_{\boldsymbol{i} \in A_{\boldsymbol{y}}} p_{\boldsymbol{i}}^{(t)} \big)} \\ &+ \frac{\sum_{\boldsymbol{y} \in \mathscr{A}_{\boldsymbol{x}}} (-1)^{|\boldsymbol{x}|-|\boldsymbol{y}|} \sum_{\boldsymbol{h} \in \mathscr{H}} p_{\boldsymbol{h}}^{(t)} \log p_{\boldsymbol{h}} G_t' \Big(\sum_{\boldsymbol{i} \in A_{\boldsymbol{y}}} p_{\boldsymbol{i}}^{(t)} \Big) I_{A_{\boldsymbol{y}}}(\boldsymbol{h})}{\sum_{\boldsymbol{y} \in \mathscr{A}_{\boldsymbol{x}}} (-1)^{|\boldsymbol{x}|-|\boldsymbol{y}|} G_t \Big(\sum_{\boldsymbol{i} \in A_{\boldsymbol{y}}} p_{\boldsymbol{i}}^{(t)} \Big)} + \tilde{\tilde{A}}_t. \end{aligned} \tag{A.9d}$$

The Q-function (A.6c) becomes

$$\begin{aligned} Q(\boldsymbol{\theta} \,|\, \boldsymbol{\theta}_t) = &-N \log \left(e^{\lambda} - 1 \right) \\ &+ \log \lambda \sum_{\boldsymbol{x} \in \mathscr{O}} n_{\boldsymbol{x}} \frac{\sum_{\boldsymbol{y} \in \mathscr{A}_{\boldsymbol{x}}} (-1)^{|\boldsymbol{x}|-|\boldsymbol{y}|} \sum_{\boldsymbol{h} \in A_{\boldsymbol{y}}} p_{\boldsymbol{h}}^{(t)} G_t' \Big(\sum_{\boldsymbol{i} \in A_{\boldsymbol{y}}} p_{\boldsymbol{i}}^{(t)} \Big)}{\sum_{\boldsymbol{y} \in \mathscr{A}_{\boldsymbol{x}}} (-1)^{|\boldsymbol{x}|-|\boldsymbol{y}|} G_t \Big(\sum_{\boldsymbol{i} \in A_{\boldsymbol{y}}} p_{\boldsymbol{i}}^{(t)} \Big)} \\ &+ \sum_{\boldsymbol{x} \in \mathscr{O}} n_{\boldsymbol{x}} \frac{\sum_{\boldsymbol{y} \in \mathscr{A}_{\boldsymbol{x}}} (-1)^{|\boldsymbol{x}|-|\boldsymbol{y}|} \sum_{\boldsymbol{h} \in \mathscr{H}} p_{\boldsymbol{h}}^{(t)} \log p_{\boldsymbol{h}} G_t' \Big(\sum_{\boldsymbol{i} \in A_{\boldsymbol{y}}} p_{\boldsymbol{i}}^{(t)} \Big) I_{A_{\boldsymbol{y}}}(\boldsymbol{h})}{\sum_{\boldsymbol{y} \in \mathscr{A}_{\boldsymbol{x}}} (-1)^{|\boldsymbol{x}|-|\boldsymbol{y}|} G_t \Big(\sum_{\boldsymbol{i} \in A_{\boldsymbol{y}}} p_{\boldsymbol{i}}^{(t)} \Big)} \\ &+ \tilde{\tilde{A}}_t. \end{aligned} \tag{A.9e}$$

Rearranging the sums in the third term yields

$$\begin{aligned} Q(\boldsymbol{\theta} \,|\, \boldsymbol{\theta}_t) = &-N \log \left(e^{\lambda} - 1 \right) \\ &+ \log \lambda \sum_{\boldsymbol{x} \in \mathscr{O}} n_{\boldsymbol{x}} \frac{\sum_{\boldsymbol{y} \in \mathscr{A}_{\boldsymbol{x}}} (-1)^{|\boldsymbol{x}|-|\boldsymbol{y}|} \sum_{\boldsymbol{h} \in A_{\boldsymbol{y}}} p_{\boldsymbol{h}}^{(t)} G_t' \Big(\sum_{\boldsymbol{i} \in A_{\boldsymbol{y}}} p_{\boldsymbol{i}}^{(t)} \Big)}{\sum_{\boldsymbol{y} \in \mathscr{A}_{\boldsymbol{x}}} (-1)^{|\boldsymbol{x}|-|\boldsymbol{y}|} G_t \Big(\sum_{\boldsymbol{i} \in A_{\boldsymbol{y}}} p_{\boldsymbol{i}}^{(t)} \Big)} \\ &+ \sum_{\boldsymbol{h} \in \mathscr{H}} \frac{\sum_{\boldsymbol{x} \in \mathscr{O}} n_{\boldsymbol{x}} \sum_{\boldsymbol{y} \in \mathscr{A}_{\boldsymbol{x}}} (-1)^{|\boldsymbol{x}|-|\boldsymbol{y}|} p_{\boldsymbol{h}}^{(t)} \log p_{\boldsymbol{h}} G_t' \Big(\sum_{\boldsymbol{i} \in A_{\boldsymbol{y}}} p_{\boldsymbol{i}}^{(t)} \Big) I_{A_{\boldsymbol{y}}}(\boldsymbol{h})}{\sum_{\boldsymbol{y} \in \mathscr{A}_{\boldsymbol{x}}} (-1)^{|\boldsymbol{x}|-|\boldsymbol{y}|} G_t \Big(\sum_{\boldsymbol{i} \in A_{\boldsymbol{y}}} p_{\boldsymbol{i}}^{(t)} \Big)} \\ &+ \tilde{\tilde{A}}_t. \end{aligned} \tag{A.9f}$$

Let

$$B_t := \sum_{\boldsymbol{x}\in\mathscr{O}} n_{\boldsymbol{x}} \frac{\sum\limits_{\boldsymbol{y}\in\mathscr{A}_{\boldsymbol{x}}} (-1)^{|\boldsymbol{x}|-|\boldsymbol{y}|} \sum\limits_{\boldsymbol{h}\in A_{\boldsymbol{y}}} p_{\boldsymbol{h}}^{(t)} G_t'\Big(\sum\limits_{\boldsymbol{i}\in A_{\boldsymbol{y}}} p_{\boldsymbol{i}}^{(t)}\Big)}{\sum\limits_{\boldsymbol{y}\in\mathscr{A}_{\boldsymbol{x}}} (-1)^{|\boldsymbol{x}|-|\boldsymbol{y}|} G_t\Big(\sum\limits_{\boldsymbol{i}\in A_{\boldsymbol{y}}} p_{\boldsymbol{i}}^{(t)}\Big)}, \tag{A.10a}$$

and

$$C_{\boldsymbol{h}}^{(t)} := p_{\boldsymbol{h}}^{(t)} \sum_{\boldsymbol{x}\in\mathscr{O}} n_{\boldsymbol{x}} \frac{\sum\limits_{\boldsymbol{y}\in\mathscr{A}_{\boldsymbol{x}}} (-1)^{|\boldsymbol{x}|-|\boldsymbol{y}|} G_t'\Big(\sum\limits_{\boldsymbol{i}\in A_{\boldsymbol{y}}} p_{\boldsymbol{i}}^{(t)}\Big) I_{A_{\boldsymbol{y}}}(\boldsymbol{h})}{\sum\limits_{\boldsymbol{y}\in\mathscr{A}_{\boldsymbol{x}}} (-1)^{|\boldsymbol{x}|-|\boldsymbol{y}|} G_t\Big(\sum\limits_{\boldsymbol{i}\in A_{\boldsymbol{y}}} p_{\boldsymbol{i}}^{(t)}\Big)}, \tag{A.10b}$$

such that the Q-function is rewritten as

$$Q(\boldsymbol{\theta}\,|\,\boldsymbol{\theta}_t) = -N\log(e^{\lambda}-1) + B_t\log\lambda + \sum_{\boldsymbol{h}\in\mathscr{H}} C_{\boldsymbol{h}}^{(t)} \log p_{\boldsymbol{h}} + \tilde{\tilde{A}}_t. \tag{A.10c}$$

Maximization (M)-step

Recall that, the parameter space is denoted by Θ. In the M-step of the EM-algorithm, the Q-function is maximized with respect to $\boldsymbol{\theta}$ (while $\boldsymbol{\theta}_t$ is constant). The updated parameters $\boldsymbol{\theta}_{t+1}$ in iteration $t+1$ are obtained as

$$\boldsymbol{\theta}_{t+1} = \arg\max_{\boldsymbol{\theta}\in\Theta} Q(\boldsymbol{\theta}\,|\,\boldsymbol{\theta}_t).$$

The Q-function is maximized using the method of Lagrange-multipliers under the constraint $\sum\limits_{\boldsymbol{h}\in\mathscr{H}} p_{\boldsymbol{h}} = 1$. The Lagrangian function is

$$\Lambda(\boldsymbol{\theta}\,|\,\boldsymbol{\theta}_t) := Q(\boldsymbol{\theta}\,|\,\boldsymbol{\theta}_t) - \beta\Big(\sum_{\boldsymbol{h}\in\mathscr{H}} p_{\boldsymbol{h}} - 1\Big),$$

where β is the Lagrange multiplier. The parameters $\boldsymbol{\theta}_{t+1}$ in step $t+1$ of the EM-algorithm are found by solving

$$\nabla\Lambda(\boldsymbol{\theta}\,|\,\boldsymbol{\theta}_t) = \mathbf{0}.$$

The Lagrangian function is given by

$$\Lambda(\boldsymbol{\theta}\,|\,\boldsymbol{\theta}_t) = -N\log(e^{\lambda}-1) + B_t\log\lambda + \sum_{\boldsymbol{h}\in\mathscr{H}} C_{\boldsymbol{h}}^{(t)} \log p_{\boldsymbol{h}} - \beta\Big(\sum_{\boldsymbol{h}\in\mathscr{H}} p_{\boldsymbol{h}} - 1\Big) + \tilde{\tilde{A}}_t, \tag{A.11}$$

and its gradient by

$$\frac{\partial\Lambda(\boldsymbol{\theta}\,|\,\boldsymbol{\theta}_t)}{\partial\lambda} = -\frac{Ne^{\lambda}}{e^{\lambda}-1} + \frac{B_t}{\lambda}, \tag{A.12a}$$

$$\frac{\partial\Lambda(\boldsymbol{\theta}\,|\,\boldsymbol{\theta}_t)}{\partial p_{\boldsymbol{h}}} = C_{\boldsymbol{h}}^{(t)}\frac{1}{p_{\boldsymbol{h}}} - \beta, \tag{A.12b}$$

and

$$\frac{\partial \Lambda(\boldsymbol{\theta} \,|\, \boldsymbol{\theta}_t)}{\partial \beta} = -\sum_{\boldsymbol{h} \in \mathscr{H}} p_{\boldsymbol{h}} + 1. \tag{A.12c}$$

To obtain the parameter update $\boldsymbol{\theta}_{t+1}$, we equate the above components of the gradient to zero. Equating (A.12b) to 0 gives

$$p_{\boldsymbol{h}} = \frac{C_{\boldsymbol{h}}^{(t)}}{\beta}.$$

Substituting this expression into (A.12c) and equating the resulting term to 0 yields

$$\beta = \sum_{\boldsymbol{h} \in \mathscr{H}} C_{\boldsymbol{h}}^{(t)}.$$

Combining the last two equations gives the frequency of haplotype $\boldsymbol{h}$ in step $t+1$, namely

$$p_{\boldsymbol{h}}^{(t+1)} = \frac{C_{\boldsymbol{h}}^{(t)}}{\sum\limits_{\boldsymbol{h} \in \mathscr{H}} C_{\boldsymbol{h}}^{(t)}}. \tag{A.13}$$

The MOI parameter λ in step $t+1$ is obtained by equating (A.12a) to 0, i.e.,

$$-\frac{N e^{\lambda}}{e^{\lambda} - 1} + \frac{B_t}{\lambda} = 0.$$

This non-linear equation has no closed solution and needs to be solved numerically. This can be achieved by the 1-dimensional Newton-Raphson method. Let the function f be defined as

$$f(\lambda) := \frac{\lambda e^{\lambda}}{e^{\lambda} - 1} - \frac{B_t}{N}. \tag{A.14a}$$

The 1-dimensional Newton-Raphson method yields the recursion equation

$$\lambda_{\tau+1} = \lambda_\tau - \frac{f(\lambda_\tau)}{f'(\lambda_\tau)}, \tag{A.14b}$$

where

$$f'(\lambda) = \frac{(1+\lambda)(1-e^{-\lambda}) - \lambda}{(1-e^{-\lambda})^2}. \tag{A.14c}$$

Therefore, the recursion equation is given by

$$\lambda_{\tau+1} = \lambda_\tau - \frac{\lambda_\tau - \frac{B_t}{N}(1-e^{-\lambda_\tau})}{1 + \lambda_\tau - \frac{\lambda_\tau}{1-e^{-\lambda_\tau}}}. \tag{A.14d}$$

The value of the MOI parameter λ_{t+1} in step $t+1$ of the EM-algorithm is the limit of the recursion (A.14d).

Prevalence estimates

First, we derive expression (6) for the probability of observing haplotype $\boldsymbol{h}$ in an unambiguous observation, $\tilde{q}_{\boldsymbol{h}}$. Recall the definition of the set $U_{\boldsymbol{h}}$ of all haplotype $\boldsymbol{i}$, which yields unambiguous observations together with haplotype $\boldsymbol{h}$, defined in (7). Note that there are exactly $|U_{\boldsymbol{h}}| = \sum_{k=1}^{L} n_k - L$ haplotypes $U_{\boldsymbol{h}}$.

Some rearrangement of (8a) in order to use the binomial theorem gives

$$\begin{aligned}
\tilde{q}_{\boldsymbol{h}} &= \sum_{m=1}^{\infty} \kappa_m \sum_{\boldsymbol{i}\in U_{\boldsymbol{h}}} \sum_{m_{\boldsymbol{i}}=1}^{m-1} \binom{m}{m_{\boldsymbol{i}}} p_{\boldsymbol{i}}^{m_{\boldsymbol{i}}} p_{\boldsymbol{h}}^{m-m_{\boldsymbol{i}}} + \sum_{m=1}^{\infty} \kappa_m p_{\boldsymbol{h}}^{m} \\
&= \sum_{m=1}^{\infty} \kappa_m \sum_{\boldsymbol{i}\in U_{\boldsymbol{h}}} \sum_{m_{\boldsymbol{i}}=0}^{m} \binom{m}{m_{\boldsymbol{i}}} p_{\boldsymbol{i}}^{m_{\boldsymbol{i}}} p_{\boldsymbol{h}}^{m-m_{\boldsymbol{i}}} - \sum_{m=1}^{\infty} \kappa_m \sum_{\boldsymbol{i}\in U_{\boldsymbol{h}}} p_{\boldsymbol{i}}^{m} - (|U_{\boldsymbol{h}}|-1) \sum_{m=1}^{\infty} \kappa_m p_{\boldsymbol{h}}^{m} \\
&= \sum_{\boldsymbol{i}\in U_{\boldsymbol{h}}} \sum_{m=1}^{\infty} \kappa_m (p_{\boldsymbol{h}} + p_{\boldsymbol{i}})^m - \sum_{\boldsymbol{i}\in U_{\boldsymbol{h}}} \sum_{m=1}^{\infty} \kappa_m p_{\boldsymbol{i}}^{m} - (|U_{\boldsymbol{h}}|-1) \sum_{m=1}^{\infty} \kappa_m p_{\boldsymbol{h}}^{m}.
\end{aligned}$$

Using the definition of the PGF (48b) yields

$$\tilde{q}_{\boldsymbol{h}} = \sum_{\boldsymbol{i}\in U_{\boldsymbol{h}}} \Big(G(p_{\boldsymbol{h}} + p_{\boldsymbol{i}}) - G(p_{\boldsymbol{i}}) \Big) - (|U_{\boldsymbol{h}}|-1) G(p_{\boldsymbol{h}}).$$

Therefore,

$$\tilde{q}_{\boldsymbol{h}} = \sum_{\boldsymbol{i}\in U_{\boldsymbol{h}}} \Big(G(p_{\boldsymbol{h}} + p_{\boldsymbol{i}}) - G(p_{\boldsymbol{i}}) \Big) - \Big(\sum_{k=1}^{L} n_k - L - 1 \Big) G(p_{\boldsymbol{h}}). \tag{A.15a}$$

Next, the probability of all unambiguous observations $P(\tilde{\mathscr{O}})$ is derived. Here, one cannot just sum over all quantities $\tilde{q}_{\boldsymbol{h}}$, because the sets $U_{\boldsymbol{h}}$ are not pairwise disjoint. Specifically, the correct expression is

$$P(\tilde{\mathscr{O}}) := \sum_{\boldsymbol{h}\in\mathscr{H}} \left(\frac{1}{2} \sum_{m=1}^{\infty} \kappa_m \sum_{\boldsymbol{i}\in U_{\boldsymbol{h}}} \sum_{m_{\boldsymbol{i}}=1}^{m-1} \binom{m}{m_{\boldsymbol{i}}} p_{\boldsymbol{i}}^{m_{\boldsymbol{i}}} p_{\boldsymbol{h}}^{m-m_{\boldsymbol{i}}} + \sum_{m=1}^{\infty} \kappa_m p_{\boldsymbol{h}}^{m} \right)$$

where the factor $\frac{1}{2}$ adjust for the fact that for any two haplotypes $\boldsymbol{h}$ and $\boldsymbol{i}$, $\boldsymbol{i} \in U_{\boldsymbol{h}}$, then $\boldsymbol{h} \in U_{\boldsymbol{i}}$. In other words, the factor $\frac{1}{2}$ guarantees that terms are not counted twice. Following the same steps as above, while considering that each haplotype $\boldsymbol{h}$ is contained in exactly $\sum_{k=1}^{L} n_k - L$ sets $U_{\boldsymbol{i}}$, leads to

$$\begin{aligned}
P(\tilde{\mathscr{O}}) := \sum_{\boldsymbol{h}\in\mathscr{H}} \Bigg(&\frac{1}{2} \sum_{m=1}^{\infty} \kappa_m \sum_{\boldsymbol{i}\in U_{\boldsymbol{h}}} \sum_{m_{\boldsymbol{i}}=0}^{m} \binom{m}{m_{\boldsymbol{i}}} p_{\boldsymbol{i}}^{m_{\boldsymbol{i}}} p_{\boldsymbol{h}}^{m-m_{\boldsymbol{i}}} \\
&- \frac{1}{2} \sum_{m=1}^{\infty} \kappa_m \sum_{\boldsymbol{i}\in U_{\boldsymbol{h}}} p_{\boldsymbol{i}}^{m} - \Big(\frac{1}{2} \Big(\sum_{k=1}^{L} n_k - L \Big) - 1 \Big) \sum_{m=1}^{\infty} \kappa_m p_{\boldsymbol{h}}^{m} \Bigg).
\end{aligned}$$

Using the binomial theorem and the definition of the generating function leads to

$$P(\tilde{\mathscr{O}}) = \sum_{\boldsymbol{h}\in\mathscr{H}} \left(\frac{1}{2} \sum_{\boldsymbol{i}\in U_{\boldsymbol{h}}} \Big(G(p_{\boldsymbol{h}} + p_{\boldsymbol{i}}) - G(p_{\boldsymbol{i}}) \Big) - \Big(\frac{1}{2} \Big(\sum_{k=1}^{L} n_k - L \Big) - 1 \Big) G(p_{\boldsymbol{h}}) \right).$$

Fisher information matrix

Assume a twice-differentiable log-likelihood function L with parameter space

$$\Theta := \mathbb{R}^+ \times \mathcal{S}_H = \Big\{(\lambda, \boldsymbol{p}) \,|\, \lambda > 0 \text{ and } \sum_{k=1}^{H} p_k = 1,\, 0 \leq p_k \leq 1, k \in 1, \ldots, H\Big\},$$

where $\mathcal{S}_H$ is an $(H-1)$-dimensional simplex. Hence, the parameter space is not an open set in a real dimensional space. Particularly, one parameter in the simplex is redundant and can be expressed by the others, e.g., $p_H = 1 - \sum_{k=1}^{H-1} p_k$. Therefore, caution must be taken when deriving the asymptotic variance as the inverse of the Fisher information. Namely, the underlying theory assumes that the parameter space is an open real-dimensional set. To derive the asymptotic variance, one of the following approaches is possible: (i) one of the redundant parameters can be substituted by an expression of the others; (ii) the parameter space can be embedded in a higher dimensional space by introducing a nuisance parameter.

While the first approach is more straightforward, it has two disadvantages. First, the expressions for the derivatives and hence the Fisher information is typically more complicated. This becomes obvious for the model in the main text in the special case of a single molecular marker (see [3]). Second, the asymptotic covariance matrix does neither contain the variance of the eliminated parameter, nor its covariance with the other parameters. For instance, when substituting $p_H = 1 - \sum_{k=1}^{H-1} p_k$,

$$\mathrm{Cov}(\lambda, p_H) = \mathrm{Cov}(p_H, \lambda) = \mathrm{Cov}\left(1 - \sum_{i=1}^{H-1} p_i, \lambda\right) = -\sum_{i=1}^{H-1} \mathrm{Cov}(p_i, \lambda). \tag{A.16a}$$

Furthermore,

$$\mathrm{Cov}(p_j, p_H) = \mathrm{Cov}(p_H, p_j) = \mathrm{Cov}\left(1 - \sum_{i=1}^{H-1} p_i, p_j\right) = -\sum_{i=1}^{H-1} \mathrm{Cov}(p_i, p_j), \tag{A.16b}$$

and

$$\begin{aligned} \mathrm{Var}(p_H) &= \mathrm{Cov}(p_H, p_H) = \mathrm{Cov}\left(1 - \sum_{k=1}^{H-1} p_k, 1 - \sum_{k=1}^{H-1} p_k\right) = \sum_{i,j=1}^{H-1} \mathrm{Cov}(p_i, p_j) \\ &= \sum_{k=1}^{H-1} \mathrm{Var}(p_k) + \sum_{\substack{i,j=1: \\ i \neq j}}^{H-1} \mathrm{Cov}(p_i, p_j). \end{aligned} \tag{A.16c}$$

Because of the aforementioned disadvantages, we pursue with the second approach, namely to introduce a nuisance parameter.

Higher dimensional embedding

By introducing a Lagrange multiplier as a nuisance parameter, it is guaranteed that the function

$$\Lambda(\boldsymbol{\theta}, \beta) \coloneqq L(\boldsymbol{\theta}) + \beta\Big(1 - \sum_{k=1}^{H} p_k\Big) \tag{A.17}$$

attains the maximum for the same parameter choices $\boldsymbol{\theta}$ as the log-likelihood function L. Note, $\Lambda(\boldsymbol{\theta}, \beta)$ is not a log-likelihood function and is defined on the domain

$$\tilde{\Theta} \coloneqq \mathbb{R}^{+} \times (0,1)^{H} \times \mathbb{R}.$$

Let $\tilde{\boldsymbol{\theta}} \coloneqq (\boldsymbol{\theta}, \beta) \in \tilde{\Theta}$. Under certain regularity conditions, which hold in most cases, the entries of the "Fisher information matrix" $\tilde{\mathcal{I}}$ are defined by (see below for more discussion)

$$\tilde{\mathcal{I}}_{i,j} \coloneqq -\mathbb{E}\left[\frac{\partial^2 \Lambda}{\partial \tilde{\theta}_i \partial \tilde{\theta}_j}(\tilde{\boldsymbol{\theta}})\right], \tag{A.18}$$

with $\tilde{\theta}_i$, $\tilde{\theta}_j \in \tilde{\Theta}$. In particular, for $\tilde{\theta}_i = \tilde{\theta}_j = \lambda$,

$$\tilde{\mathcal{I}}_{\lambda,\lambda} \coloneqq -\mathbb{E}\left[\frac{\partial^2 \Lambda}{\partial \lambda^2}(\tilde{\boldsymbol{\theta}})\right] = -\mathbb{E}\left[\frac{\partial^2 L}{\partial \lambda^2}(\boldsymbol{\theta})\right] \eqqcolon \mathcal{I}_{\lambda,\lambda}. \tag{A.19}$$

For $\tilde{\theta}_i = \lambda$ and $\tilde{\theta}_j = p_i$,

$$\tilde{\mathcal{I}}_{\lambda,p_i} \coloneqq -\mathbb{E}\left[\frac{\partial^2 \Lambda}{\partial \lambda \partial p_i}(\tilde{\boldsymbol{\theta}})\right] = -\mathbb{E}\left[\frac{\partial^2 L}{\partial \lambda \partial p_i}(\boldsymbol{\theta})\right] \eqqcolon \mathcal{I}_{\lambda,p_i}. \tag{A.20}$$

For $\tilde{\theta}_i = \lambda$ and $\tilde{\theta}_j = \beta$,

$$\tilde{\mathcal{I}}_{\lambda,\beta} \coloneqq -\mathbb{E}\left[\frac{\partial^2 \Lambda}{\partial \lambda \partial \beta}(\tilde{\boldsymbol{\theta}})\right] = 0. \tag{A.21}$$

Moreover, for $\tilde{\theta}_i = \tilde{\theta}_j = p_i$,

$$\tilde{\mathcal{I}}_{p_i,p_i} \coloneqq -\mathbb{E}\left[\frac{\partial^2 \Lambda}{\partial p_i^2}(\tilde{\boldsymbol{\theta}})\right] = -\mathbb{E}\left[\frac{\partial^2 L}{\partial p_i^2}(\boldsymbol{\theta})\right] \eqqcolon \mathcal{I}_{p_i,p_i}, \tag{A.22}$$

for $\tilde{\theta}_i = p_i, \tilde{\theta}_j = p_j$,

$$\tilde{\mathcal{I}}_{p_i,p_j} \coloneqq -\mathbb{E}\left[\frac{\partial^2 \Lambda}{\partial p_i \partial p_j}(\tilde{\boldsymbol{\theta}})\right] = -\mathbb{E}\left[\frac{\partial^2 L}{\partial p_i \partial p_j}(\boldsymbol{\theta})\right] \eqqcolon \mathcal{I}_{p_i,p_j}, \tag{A.23}$$

for $\tilde{\theta}_i = p_i, \tilde{\theta}_j = \beta$,

$$\tilde{\mathcal{I}}_{p_i,\beta} \coloneqq -\mathbb{E}\left[\frac{\partial^2 \Lambda}{\partial p_i \partial \beta}(\tilde{\boldsymbol{\theta}})\right] = 1, \tag{A.24}$$

and for $\tilde{\theta}_i = \tilde{\theta}_j = \beta$,

$$\tilde{\mathcal{I}}_{\beta,\beta} = -\mathbb{E}\left[\frac{\partial^2 \Lambda}{\partial \beta^2}(\tilde{\boldsymbol{\theta}})\right] = 0. \tag{A.25}$$

The Fisher information matrix hence has the form

$$\tilde{\mathcal{I}}(\tilde{\boldsymbol{\theta}}) = \begin{pmatrix} \mathcal{I}_{\lambda,\lambda} & \mathcal{I}_{\lambda,p_1} & \cdots & \mathcal{I}_{\lambda,p_H} & 0 \\ \mathcal{I}_{p_1,\lambda} & \mathcal{I}_{p_1,p_1} & \cdots & \mathcal{I}_{p_1,p_H} & 1 \\ \vdots & \vdots & \ddots & \vdots & \vdots \\ \mathcal{I}_{p_H,\lambda} & \mathcal{I}_{p_H,p_1} & \cdots & \mathcal{I}_{p_H,p_H} & 1 \\ 0 & 1 & \cdots & 1 & 0 \end{pmatrix}. \tag{A.26}$$

Alternative derivation of the asymptotic covariance matrix

The alternative approach to derive the CRLB is to eliminate a redundant parameter. Recall that $\boldsymbol{p}$ is an element of the simplex $\mathcal{S}_H$, i.e., $\sum\limits_{k=1}^{H} p_k = 1$. Hence, p_H can be rewritten as $p_H = 1 - \sum\limits_{k=1}^{H-1} p_k = f(p_1, \dots, p_{H-1})$ and the parameters in the reduced space $\boldsymbol{\theta}' = (\lambda, p_1, \dots, p_{H-1}) \in \Theta' \subseteq \mathbb{R}^+ \times (0,1)^{H-1}$ transform to $\boldsymbol{\theta} = \big(\lambda, p_1, \dots, p_{H-1}, f(p_1, \dots, p_{H-1})\big)$. In the reduced space, the likelihood function becomes

$$l(\boldsymbol{\theta}') \coloneqq L\big(\lambda, p_1, \dots, p_{H-1}, f(p_1, \dots, p_{H-1})\big), \tag{A.27}$$

and the entries of the Fisher information matrix $\mathcal{I}'$ are given by

$$\mathcal{I}'_{i,j} = -\mathbb{E}\left[\frac{\partial^2 l}{\partial \theta'_i \theta'_j}(\boldsymbol{\theta}')\right]. \tag{A.28}$$

For $\theta'_i = \theta'_j = \lambda$,

$$\mathcal{I}'_{\lambda,\lambda} = -\mathbb{E}\left[\frac{\partial^2 l}{\partial \lambda^2}(\boldsymbol{\theta}')\right] = -\mathbb{E}\left[\frac{\partial^2 L}{\partial \lambda^2}(\boldsymbol{\theta})\right] = \mathcal{I}_{\lambda,\lambda}. \tag{A.29}$$

For $\theta'_i = \lambda, \theta'_j = p_i$,

$$\mathcal{I}'_{\lambda,p_i} = -\mathbb{E}\left[\frac{\partial^2 l}{\partial \lambda \partial p_i}(\boldsymbol{\theta}')\right], \tag{A.30}$$

with

$$\frac{\partial^2 l}{\partial \lambda \partial p_i}(\boldsymbol{\theta}') = \frac{\partial}{\partial \lambda}\Big(\frac{\partial l}{\partial p_i}(\boldsymbol{\theta}')\Big).$$

Applying the chain rule yields,

$$\frac{\partial l}{\partial p_i}(\boldsymbol{\theta}') = \frac{\partial L}{\partial p_i}(\boldsymbol{\theta})\frac{\partial p_i}{\partial p_i} + \frac{\partial L}{\partial p_H}(\boldsymbol{\theta})\frac{\partial f}{\partial p_i}(\boldsymbol{\theta}') = \frac{\partial L}{\partial p_i}(\boldsymbol{\theta}) - \frac{\partial L}{\partial p_H}(\boldsymbol{\theta}),$$

and

$$\frac{\partial^2 l}{\partial\lambda\partial p_i}(\boldsymbol{\theta}') = \frac{\partial^2 L}{\partial\lambda\partial p_i}(\boldsymbol{\theta}) - \frac{\partial^2 L}{\partial\lambda\partial p_H}(\boldsymbol{\theta}).$$

Hence,

$$\mathcal{I}'_{\lambda,p_i} = -\mathbb{E}\left[\frac{\partial^2 L}{\partial\lambda\partial p_i}(\boldsymbol{\theta})\right] + \mathbb{E}\left[\frac{\partial^2 L}{\partial\lambda\partial p_H}(\boldsymbol{\theta})\right] = \mathcal{I}_{\lambda,p_i} - \mathcal{I}_{\lambda,p_H}. \tag{A.31}$$

Moreover, for $\theta'_i = \theta'_j = p_i$

$$\mathcal{I}'_{p_i,p_i} = -\mathbb{E}\left[\frac{\partial^2 l}{\partial p_i^2}(\boldsymbol{\theta}')\right],$$

with

$$\begin{aligned}\frac{\partial^2 l}{\partial p_i^2}(\boldsymbol{\theta}') =& \frac{\partial}{\partial p_i}\Big(\frac{\partial L}{\partial p_i}(\boldsymbol{\theta}) - \frac{\partial L}{\partial p_H}(\boldsymbol{\theta})\Big)\\ =& \frac{\partial^2 L}{\partial p_i^2}(\boldsymbol{\theta}) - 2\frac{\partial^2 L}{\partial p_i\partial p_H}(\boldsymbol{\theta}) + \frac{\partial^2 L}{\partial p_H^2}(\boldsymbol{\theta}).\end{aligned}$$

Therefore,

$$\begin{aligned}\mathcal{I}'_{p_i,p_i} =& -\mathbb{E}\left[\frac{\partial^2 L}{\partial p_i^2}(\boldsymbol{\theta})\right] + 2\mathbb{E}\left[\frac{\partial^2 L}{\partial p_i\partial p_H}(\boldsymbol{\theta})\right] - \mathbb{E}\left[\frac{\partial^2 L}{\partial p_H^2}(\boldsymbol{\theta})\right]\\ =& \mathcal{I}_{p_i,p_i} - 2\mathcal{I}_{p_i,p_H} + \mathcal{I}_{p_H,p_H}.\end{aligned} \tag{A.32}$$

Similarly, for $\theta'_i = p_i, \theta'_j = p_j$,

$$\begin{aligned}\mathcal{I}'_{p_i,p_j} =& -\mathbb{E}\left[\frac{\partial^2 L}{\partial p_i\partial p_j}(\boldsymbol{\theta})\right] + \mathbb{E}\left[\frac{\partial^2 L}{\partial p_i\partial p_H}(\boldsymbol{\theta})\right] + \mathbb{E}\left[\frac{\partial^2 L}{\partial p_j\partial p_H}(\boldsymbol{\theta})\right] - \mathbb{E}\left[\frac{\partial^2 L}{\partial p_H^2}(\boldsymbol{\theta})\right]\\ =& \mathcal{I}_{p_i,p_j} - \mathcal{I}_{p_i,p_H} - \mathcal{I}_{p_j,p_H} + \mathcal{I}_{p_H,p_H}.\end{aligned} \tag{A.33}$$

The Fisher information matrix in the reduced space has the form

$$\mathcal{I}'(\boldsymbol{\theta}') = \begin{pmatrix} \mathcal{I}_{\lambda,\lambda} & \mathcal{I}_{\lambda,p_1} - \mathcal{I}_{\lambda,p_H} & \cdots & \mathcal{I}_{\lambda,p_{H-1}} - \mathcal{I}_{\lambda,p_H} \\ \mathcal{I}_{\lambda,p_1} - \mathcal{I}_{\lambda,p_H} & \begin{pmatrix}\mathcal{I}_{p_1,p_1} - 2\mathcal{I}_{p_1,p_H}\\ +\mathcal{I}_{p_H,p_H}\end{pmatrix} & \cdots & \begin{pmatrix}\mathcal{I}_{p_1,p_{H-1}} - \mathcal{I}_{p_1,p_H}\\ -\mathcal{I}_{p_{H-1},p_H} + \mathcal{I}_{p_H,p_H}\end{pmatrix} \\ \vdots & \vdots & \ddots & \vdots \\ \mathcal{I}_{\lambda,p_{H-1}} - \mathcal{I}_{\lambda,p_H} & \begin{pmatrix}\mathcal{I}_{p_1,p_{H-1}} - \mathcal{I}_{p_1,p_H}\\ -\mathcal{I}_{p_{H-1},p_H} + \mathcal{I}_{p_H,p_H}\end{pmatrix} & \cdots & \begin{pmatrix}\mathcal{I}_{p_{H-1},p_{H-1}} - 2\mathcal{I}_{p_{H-1},p_H}\\ +\mathcal{I}_{p_H,p_H}\end{pmatrix} \end{pmatrix}.$$

Equivalence of alternative approaches to derive the inverse Fisher information

Here, it is shown that inverting the matrixes $\tilde{\mathcal{I}}$ and $\mathcal{I}'$ yield equivalent results concerning the asymptotic covariances of the original model parameters. The inverse of

the “Fisher information matrix” including the nuisance parameter $\tilde{\mathcal{I}}$ can be calculated by applying Cramer’s rule. First, we find the determinant of $\tilde{\mathcal{I}}$, i.e.,

$$\det \tilde{\mathcal{I}}(\tilde{\boldsymbol{\theta}}) = \begin{vmatrix} \mathcal{I}_{\lambda,\lambda} & \mathcal{I}_{\lambda,p_1} & \cdots & \mathcal{I}_{\lambda,p_{H-1}} & \mathcal{I}_{\lambda,p_H} & 0 \\ \mathcal{I}_{p_1,\lambda} & \mathcal{I}_{p_1,p_1} & \cdots & \mathcal{I}_{p_1,p_{H-1}} & \mathcal{I}_{p_1,p_H} & 1 \\ \vdots & \vdots & \ddots & \vdots & \vdots & \\ \mathcal{I}_{p_{H-1},\lambda} & \mathcal{I}_{p_{H-1},p_1} & \cdots & \mathcal{I}_{p_{H-1},p_{H-1}} & \mathcal{I}_{p_{H-1},p_H} & 1 \\ \mathcal{I}_{p_H,\lambda} & \mathcal{I}_{p_H,p_1} & \cdots & \mathcal{I}_{p_H,p_{H-1}} & \mathcal{I}_{p_H,p_H} & 1 \\ 0 & 1 & \cdots & 1 & 1 & 0 \end{vmatrix}.$$

The determinant remains unchanged when subtracting $\mathcal{I}_{\lambda,p_H}$ times the last row from the first, and $\mathcal{I}_{p_k,p_H}$ times the last row from the k-th row (for $k = 2, \ldots, H+1$). This yields

$$\det \tilde{\mathcal{I}}(\tilde{\boldsymbol{\theta}}) = \begin{vmatrix} \mathcal{I}_{\lambda,\lambda} & \mathcal{I}_{\lambda,p_1} - \mathcal{I}_{\lambda,p_H} & \cdots & \mathcal{I}_{\lambda,p_{H-1}} - \mathcal{I}_{\lambda,p_H} & 0 & 0 \\ \mathcal{I}_{p_1,\lambda} & \mathcal{I}_{p_1,p_1} - \mathcal{I}_{p_1,p_H} & \cdots & \mathcal{I}_{p_1,p_{H-1}} - \mathcal{I}_{p_1,p_H} & 0 & 1 \\ \vdots & \vdots & \ddots & \vdots & \vdots & \\ \mathcal{I}_{p_{H-1},\lambda} & \mathcal{I}_{p_{H-1},p_1} - \mathcal{I}_{p_{H-1},p_H} & \cdots & \mathcal{I}_{p_{H-1},p_{H-1}} - \mathcal{I}_{p_{H-1},p_H} & 0 & 1 \\ \mathcal{I}_{p_H,\lambda} & \mathcal{I}_{p_H,p_1} - \mathcal{I}_{p_H,p_H} & \cdots & \mathcal{I}_{p_H,p_{H-1}} - \mathcal{I}_{p_H,p_H} & 0 & 1 \\ 0 & 1 & \cdots & 1 & 1 & 0 \end{vmatrix}.$$

Next, the determinant remains unchanged when subtracting $\mathcal{I}_{p_H,\lambda}$ times the last column from the first, and $\mathcal{I}_{p_k,p_H} - \mathcal{I}_{p_H,p_H}$ times the last column from the k-th column (for $k = 2, \ldots, H+1$), which yields

$$\det \tilde{\mathcal{I}}(\tilde{\boldsymbol{\theta}}) = \begin{vmatrix} \mathcal{I}_{\lambda,\lambda} & \mathcal{I}_{\lambda,p_1} - \mathcal{I}_{\lambda,p_H} & \cdots & \mathcal{I}_{\lambda,p_{H-1}} - \mathcal{I}_{\lambda,p_H} & 0 & 0 \\ \mathcal{I}_{p_1,\lambda} - \mathcal{I}_{p_H,\lambda} & \begin{pmatrix} \mathcal{I}_{p_1,p_1} - 2\mathcal{I}_{p_1,p_H} \\ +\mathcal{I}_{p_H,p_H} \end{pmatrix} & \cdots & \begin{pmatrix} \mathcal{I}_{p_1,p_{H-1}} - \mathcal{I}_{p_1,p_H} \\ -\mathcal{I}_{p_H,p_{H-1}} + \mathcal{I}_{p_H,p_H} \end{pmatrix} & 0 & 1 \\ \vdots & \vdots & \ddots & \vdots & \vdots & \vdots \\ \mathcal{I}_{p_{H-1},\lambda} - \mathcal{I}_{p_H,\lambda} & \begin{pmatrix} \mathcal{I}_{p_1,p_{H-1}} - \mathcal{I}_{p_1,p_H} \\ -\mathcal{I}_{p_{H-1},p_H} + \mathcal{I}_{p_H,p_H} \end{pmatrix} & \cdots & \begin{pmatrix} \mathcal{I}_{p_{H-1},p_{H-1}} - 2\mathcal{I}_{p_{H-1},p_H} \\ +\mathcal{I}_{p_H,p_H} \end{pmatrix} & 0 & 1 \\ 0 & 0 & \cdots & 0 & 0 & 1 \\ 0 & 1 & \cdots & 1 & 1 & 0 \end{vmatrix}.$$

Developing the determinant first by column $H+1$ and the resulting determinant by the last row yields

$$\det \tilde{\mathcal{I}}(\tilde{\boldsymbol{\theta}}) = -\begin{vmatrix} \mathcal{I}_{\lambda,\lambda} & \mathcal{I}_{\lambda,p_1} - \mathcal{I}_{\lambda,p_H} & \cdots & \mathcal{I}_{\lambda,p_{H-1}} - \mathcal{I}_{\lambda,p_H} & 0 \\ \mathcal{I}_{p_1,\lambda} - \mathcal{I}_{p_H,\lambda} & \begin{pmatrix} \mathcal{I}_{p_1,p_1} - 2\mathcal{I}_{p_1,p_H} \\ +\mathcal{I}_{p_H,p_H} \end{pmatrix} & \cdots & \begin{pmatrix} \mathcal{I}_{p_1,p_{H-1}} - \mathcal{I}_{p_1,p_H} \\ -\mathcal{I}_{p_H,p_{H-1}} + \mathcal{I}_{p_H,p_H} \end{pmatrix} & 1 \\ \vdots & \vdots & \ddots & \vdots & \vdots \\ \mathcal{I}_{p_{H-1},\lambda} - \mathcal{I}_{p_H,\lambda} & \begin{pmatrix} \mathcal{I}_{p_1,p_{H-1}} - \mathcal{I}_{p_1,p_H} \\ -\mathcal{I}_{p_{H-1},p_H} + \mathcal{I}_{p_H,p_H} \end{pmatrix} & \cdots & \begin{pmatrix} \mathcal{I}_{p_{H-1},p_{H-1}} - 2\mathcal{I}_{p_{H-1},p_H} \\ +\mathcal{I}_{p_H,p_H} \end{pmatrix} & 1 \\ 0 & 0 & \cdots & 0 & 1 \end{vmatrix}$$

$$= -\begin{vmatrix} \mathcal{I}_{\lambda,\lambda} & \mathcal{I}_{\lambda,p_1} - \mathcal{I}_{\lambda,p_H} & \cdots & \mathcal{I}_{\lambda,p_{H-1}} - \mathcal{I}_{\lambda,p_H} \\ \mathcal{I}_{p_1,\lambda} - \mathcal{I}_{p_H,\lambda} & \begin{pmatrix} \mathcal{I}_{p_1,p_1} - 2\mathcal{I}_{p_1,p_H} \\ +\mathcal{I}_{p_H,p_H} \end{pmatrix} & \cdots & \begin{pmatrix} \mathcal{I}_{p_1,p_{H-1}} - \mathcal{I}_{p_1,p_H} \\ -\mathcal{I}_{p_H,p_{H-1}} + \mathcal{I}_{p_H,p_H} \end{pmatrix} \\ \vdots & \vdots & \ddots & \\ \mathcal{I}_{p_{H-1},\lambda} - \mathcal{I}_{p_H,\lambda} & \begin{pmatrix} \mathcal{I}_{p_1,p_{H-1}} - \mathcal{I}_{p_1,p_H} \\ -\mathcal{I}_{p_{H-1},p_H} + \mathcal{I}_{p_H,p_H} \end{pmatrix} & \cdots & \begin{pmatrix} \mathcal{I}_{p_{H-1},p_{H-1}} - 2\mathcal{I}_{p_{H-1},p_H} \\ +\mathcal{I}_{p_H,p_H} \end{pmatrix} \end{vmatrix}$$

$$= -\det \mathcal{I}'(\boldsymbol{\theta}').$$

Hence,

$$\det \tilde{\mathcal{I}}(\tilde{\boldsymbol{\theta}}) = -\det \mathcal{I}'(\boldsymbol{\theta}'). \tag{A.34}$$

The same calculations with obvious modifications hold true if the k-th row and l-th column $(k, l = 1, \ldots, H-1)$ are removed from the matrix $\tilde{\mathcal{I}}(\tilde{\boldsymbol{\theta}})$, denoted $\tilde{\mathcal{I}}^{(kl)}(\tilde{\boldsymbol{\theta}})$, i.e.,

$$\det \tilde{\mathcal{I}}^{(kl)}(\tilde{\boldsymbol{\theta}}) = -\det \mathcal{I}'^{(kl)}(\boldsymbol{\theta}'). \tag{A.35}$$

Application of Cramer's rule hence gives

$$\tilde{\mathcal{I}}^{-1}_{kl}(\tilde{\boldsymbol{\theta}}) = (-1)^{k+l} \frac{\det \tilde{\mathcal{I}}^{(lk)}(\tilde{\boldsymbol{\theta}})}{\det \tilde{\mathcal{I}}(\tilde{\boldsymbol{\theta}})} = (-1)^{k+l} \frac{\mathcal{I}'^{(lk)}(\boldsymbol{\theta}')}{\det \mathcal{I}'(\boldsymbol{\theta}')} = \mathcal{I}'^{-1}_{kl}(\boldsymbol{\theta}'), \tag{A.36}$$

for $k, l = 1, \ldots, H$, i.e., the matrix $\mathcal{I}'^{-1}(\boldsymbol{\theta}')$ is the matrix obtained by deleting rows and columns $(H+1)$ and $(H+2)$ from the matrix $\tilde{\mathcal{I}}^{-1}_{kl}(\tilde{\boldsymbol{\theta}})$.

Thus, for obtaining the inverse Fisher information of a likelihood function with a parameter space involving a simplex, it is equivalent to eliminate a redundant parameter and to introduce a Lagrange multiplier as a nuisance parameter. However, in the case of eliminating a redundant parameter the matrix $\mathcal{I}'^{-1}(\boldsymbol{\theta}')$ does not contain the covariances, involving the eliminated parameter. These have to be calculated from $\mathcal{I}'^{-1}(\boldsymbol{\theta}')$ according to (A.16a)-(A.16c).

Note that the equivalence also justifies the definition of the Fisher information by (A.28). Namely, under standard regulatory conditions (existence of derivatives of the

likelihood function up to order three and interchangeability of integration and differentiation), is given by $\mathcal{I}'_{i,j} := -\mathbb{E}\left[\frac{\partial^2 l}{\partial\theta'_i\partial\theta'_j}(\boldsymbol{\theta}')\right]$. At first, this holds only for the likelihood function $l(\boldsymbol{\theta}')$ obtained by eliminating a redundant parameter. (Note that $\Lambda(\tilde{\boldsymbol{\theta}})$ is not a likelihood function.) However, the equivalence of the corresponding entries of $\tilde{\mathcal{I}}^{-1}(\tilde{\boldsymbol{\theta}})$ and $\mathcal{I}'^{-1}(\boldsymbol{\theta}')$ justify the definition (A.28). Importantly, the entries of the matrices $\tilde{\mathcal{I}}(\tilde{\boldsymbol{\theta}})$ and $\mathcal{I}'(\boldsymbol{\theta}')$ do not coincide. Therefore, in the context of the function Λ, we used quotes when referring to the Fisher information.

Furthermore, note that the calculations are still valid if the expectations in (A.28)-(A.33) are dropped. Hence, the argument is also valid for the inverse of the observed information matrix.

Entries of information matrix in higher dimensional space

For the present model, the entries of the Fisher information defined in (A.28) are derived as follows. For $\theta_i = \theta_j = \lambda$, we have

$$\begin{aligned}\mathcal{I}_{\lambda,\lambda} &= -\,\mathbb{E}\left[\frac{\partial^2 L}{\partial\lambda^2}(\boldsymbol{\theta})\right] = -\mathbb{E}\left[\frac{\partial^2}{\partial\lambda^2}\Big(\sum_{\boldsymbol{x}\in\mathscr{O}} n_{\boldsymbol{x}}\log P_{\boldsymbol{x}}\Big)\right] = -\mathbb{E}\left[\sum_{\boldsymbol{x}\in\mathscr{O}} n_{\boldsymbol{x}}\frac{\partial^2}{\partial\lambda^2}\log P_{\boldsymbol{x}}\right]\\ &= -\sum_{\boldsymbol{x}\in\mathscr{O}}\left[\mathbb{E}n_{\boldsymbol{x}}\Big(-\frac{1}{P_{\boldsymbol{x}}^2}\Big(\frac{\partial P_{\boldsymbol{x}}}{\partial\lambda}\Big)^2 + \frac{1}{P_{\boldsymbol{x}}}\frac{\partial^2 P_{\boldsymbol{x}}}{\partial\lambda^2}\Big)\right].\end{aligned}$$

We have

$$\mathbb{E}[n_{\boldsymbol{x}}] = NP_{\boldsymbol{x}} \tag{A.37}$$

since $n_{\boldsymbol{x}}$ follows a multinomial distribution with parameters N and $P_{\boldsymbol{x}}$. Thus,

$$\mathcal{I}_{\lambda,\lambda} = -\sum_{\boldsymbol{x}\in\mathscr{O}}\left[NP_{\boldsymbol{x}}\Big(-\frac{1}{P_{\boldsymbol{x}}^2}\Big(\frac{\partial P_{\boldsymbol{x}}}{\partial\lambda}\Big)^2 + \frac{1}{P_{\boldsymbol{x}}}\frac{\partial^2 P_{\boldsymbol{x}}}{\partial\lambda^2}\Big)\right] = N\sum_{\boldsymbol{x}\in\mathscr{O}}\left[\frac{1}{P_{\boldsymbol{x}}}\Big(\frac{\partial P_{\boldsymbol{x}}}{\partial\lambda}\Big)^2 - \frac{\partial^2 P_{\boldsymbol{x}}}{\partial\lambda^2}\right].$$

Recall that $P_{\boldsymbol{x}} = \sum_{\boldsymbol{y}\in\mathscr{A}_{\boldsymbol{x}}}(-1)^{|\boldsymbol{x}|-|\boldsymbol{y}|}G\Big(\sum_{\boldsymbol{h}\in A_{\boldsymbol{y}}} p_{\boldsymbol{h}}\Big)$ from (48a). Hence,

$$\frac{\partial P_{\boldsymbol{x}}}{\partial\lambda} = \sum_{\boldsymbol{y}\in\mathscr{A}_{\boldsymbol{x}}}(-1)^{|\boldsymbol{x}|-|\boldsymbol{y}|}\frac{\partial}{\partial\lambda}G\Big(\sum_{\boldsymbol{h}\in A_{\boldsymbol{y}}} p_{\boldsymbol{h}}\Big).$$

Moreover,

$$\sum_{\boldsymbol{x}\in\mathscr{O}}\frac{\partial^2 P_{\boldsymbol{x}}}{\partial\lambda^2} = \frac{\partial^2}{\partial\lambda^2}\sum_{\boldsymbol{x}\in\mathscr{O}} P_{\boldsymbol{x}} = \frac{\partial^2 1}{\partial\lambda^2} = 0$$

Together this yields

$$\begin{aligned}\mathcal{I}_{\lambda,\lambda} =& N\sum_{\boldsymbol{x}\in\mathscr{O}}\left[\Big(\sum_{\boldsymbol{y}\in\mathscr{A}_{\boldsymbol{x}}}(-1)^{|\boldsymbol{x}|-|\boldsymbol{y}|}G\Big(\sum_{\boldsymbol{h}\in A_{\boldsymbol{y}}} p_{\boldsymbol{h}}\Big)\Big)^{-1}\right.\\ &\left.\times\Big(\sum_{\boldsymbol{y}\in\mathscr{A}_{\boldsymbol{x}}}(-1)^{|\boldsymbol{x}|-|\boldsymbol{y}|}\frac{\partial}{\partial\lambda}G\Big(\sum_{\boldsymbol{h}\in A_{\boldsymbol{y}}} p_{\boldsymbol{h}}\Big)\Big)^2\right].\end{aligned} \tag{A.38a}$$

Equation (A.38a) involves the first derivative of the probability-generating function of MOI. For any z that does not depend on λ these are given by

$$\frac{\partial}{\partial\lambda}G(z) = \frac{ze^{\lambda z}}{e^{\lambda}-1} - e^{\lambda}\frac{e^{\lambda z}-1}{(e^{\lambda}-1)^2}. \tag{A.38b}$$

Using (A.37) the entries $\mathcal{I}_{\lambda,p_{\boldsymbol{i}}}$ are

$$\begin{aligned}
\mathcal{I}_{\lambda,p_{\boldsymbol{i}}} =& -\mathbb{E}\Big[\frac{\partial^2 L}{\partial\lambda\partial p_{\boldsymbol{i}}}(\boldsymbol{\theta})\Big] = -\mathbb{E}\Bigg[\sum_{\boldsymbol{x}\in\mathscr{O}} n_{\boldsymbol{x}}\frac{\partial^2}{\partial\lambda\partial p_{\boldsymbol{i}}}\log\left(P_{\boldsymbol{x}}\right)\Bigg] \\
=& -\sum_{\boldsymbol{x}\in\mathscr{O}}\Bigg[\mathbb{E}n_{\boldsymbol{x}}\Bigg(-\frac{1}{P_{\boldsymbol{x}}^2}\frac{\partial P_{\boldsymbol{x}}}{\partial\lambda}\frac{\partial P_{\boldsymbol{x}}}{\partial p_{\boldsymbol{i}}} + \frac{1}{P_{\boldsymbol{x}}}\frac{\partial^2 P_{\boldsymbol{x}}}{\partial\lambda\partial p_{\boldsymbol{i}}}\Bigg)\Bigg] \\
=& N\sum_{\boldsymbol{x}\in\mathscr{O}}\Bigg[\frac{1}{P_{\boldsymbol{x}}}\frac{\partial P_{\boldsymbol{x}}}{\partial\lambda}\frac{\partial P_{\boldsymbol{x}}}{\partial p_{\boldsymbol{i}}} - \frac{\partial^2 P_{\boldsymbol{x}}}{\partial\lambda\partial p_{\boldsymbol{j}}}\Bigg],
\end{aligned}$$

where

$$\begin{aligned}
\frac{\partial P_{\boldsymbol{x}}}{\partial\lambda}\frac{\partial P_{\boldsymbol{x}}}{\partial p_{\boldsymbol{i}}} =& \sum_{\boldsymbol{y}\in\mathscr{A}_{\boldsymbol{x}}}(-1)^{|\boldsymbol{x}|-|\boldsymbol{y}|}\frac{\partial}{\partial\lambda}G\Big(\sum_{\boldsymbol{h}\in A_{\boldsymbol{y}}}p_{\boldsymbol{h}}\Big) \\
&\times\sum_{\boldsymbol{y}\in\mathscr{A}_{\boldsymbol{x}}}(-1)^{|\boldsymbol{x}|-|\boldsymbol{y}|}\frac{\partial}{\partial p_{\boldsymbol{i}}}G\Big(\sum_{\boldsymbol{h}\in A_{\boldsymbol{y}}}p_{\boldsymbol{h}}\Big)
\end{aligned}$$

and

$$\frac{\partial^2 P_{\boldsymbol{x}}}{\partial\lambda\partial p_{\boldsymbol{i}}} = \sum_{\boldsymbol{y}\in\mathscr{A}_{\boldsymbol{x}}}(-1)^{|\boldsymbol{x}|-|\boldsymbol{y}|}\frac{\partial^2}{\partial\lambda\partial p_{\boldsymbol{i}}}G\Big(\sum_{\boldsymbol{h}\in A_{\boldsymbol{y}}}p_{\boldsymbol{h}}\Big),$$

with

$$\sum_{\boldsymbol{x}\in\mathscr{O}}\frac{\partial^2 P_{\boldsymbol{x}}}{\partial\lambda\partial p_{\boldsymbol{i}}} = \frac{\partial^2}{\partial\lambda\partial p_{\boldsymbol{i}}}\sum_{\boldsymbol{x}\in\mathscr{O}}P_{\boldsymbol{x}} = \frac{\partial^2 1}{\partial\lambda\partial p_{\boldsymbol{i}}} = 0.$$

Therefore,

$$\begin{aligned}
\mathcal{I}_{\lambda,p_{\boldsymbol{i}}} =& N\sum_{\boldsymbol{x}\in\mathscr{O}}\Bigg[\Bigg(\sum_{\boldsymbol{y}\in\mathscr{A}_{\boldsymbol{x}}}(-1)^{|\boldsymbol{x}|-|\boldsymbol{y}|}G\Big(\sum_{\boldsymbol{h}\in A_{\boldsymbol{y}}}p_{\boldsymbol{h}}\Big)\Bigg)^{-1} \\
&\times\Bigg(\sum_{\boldsymbol{y}\in\mathscr{A}_{\boldsymbol{x}}}(-1)^{|\boldsymbol{x}|-|\boldsymbol{y}|}\frac{\partial}{\partial\lambda}G\Big(\sum_{\boldsymbol{h}\in A_{\boldsymbol{y}}}p_{\boldsymbol{h}}\Big)\Bigg) \\
&\times\Bigg(\sum_{\boldsymbol{y}\in\mathscr{A}_{\boldsymbol{x}}}(-1)^{|\boldsymbol{x}|-|\boldsymbol{y}|}\frac{\partial}{\partial p_{\boldsymbol{i}}}G\Big(\sum_{\boldsymbol{h}\in A_{\boldsymbol{y}}}p_{\boldsymbol{h}}\Big)\Bigg)\Bigg].
\end{aligned} \tag{A.39a}$$

The partial derivatives of the generating function of MOI in the above equation need particular attention. While $\frac{\partial}{\partial\lambda}G\Big(\sum_{\boldsymbol{h}\in A_{\boldsymbol{y}}}p_{\boldsymbol{h}}\Big)$ is given by (A.38b),

$$\begin{aligned}
\frac{\partial}{\partial p_{\boldsymbol{i}}}G\Big(\sum_{\boldsymbol{h}\in A_{\boldsymbol{y}}}p_{\boldsymbol{h}}\Big) =& \frac{1}{e^{\lambda}-1}\frac{\partial}{\partial p_{\boldsymbol{i}}}\Bigg(\exp\big(\lambda\sum_{\boldsymbol{h}\in A_{\boldsymbol{y}}}p_{\boldsymbol{h}}\big)-1\Bigg) \\
=& \frac{\lambda}{e^{\lambda}-1}\exp\big(\lambda\sum_{\boldsymbol{h}\in A_{\boldsymbol{y}}}p_{\boldsymbol{h}}\big)I_{A_{\boldsymbol{y}}}(\boldsymbol{i}),
\end{aligned} \tag{A.39b}$$

with

$$I_{A_{\boldsymbol{y}}}(\boldsymbol{i}) = \begin{cases} 1 & \text{if } \boldsymbol{i} \in A_{\boldsymbol{y}}, \\ 0 & \text{if } \boldsymbol{i} \notin A_{\boldsymbol{y}}. \end{cases}$$

Following the same approach as above, the entries $\mathcal{I}_{p_i,p_i}$ are defined by

$$\begin{aligned} \mathcal{I}_{p_{\boldsymbol{i}},p_{\boldsymbol{i}}} =& - \mathbb{E}\Big[\frac{\partial^2 L}{\partial p_{\boldsymbol{i}}^2}(\boldsymbol{\theta})\Big] = -\mathbb{E}\left[\frac{\partial^2}{\partial p_{\boldsymbol{i}}^2}\sum_{\boldsymbol{x}\in\mathscr{O}} n_{\boldsymbol{x}} \log P_{\boldsymbol{x}}\right] \\ =& - \sum_{\boldsymbol{x}\in\mathscr{O}}\left[\mathbb{E}n_{\boldsymbol{x}}\left(- \frac{1}{P_{\boldsymbol{x}}^2}\left(\frac{\partial P_{\boldsymbol{x}}}{\partial p_{\boldsymbol{i}}}\right)^2 + \frac{1}{P_{\boldsymbol{x}}}\frac{\partial^2 P_{\boldsymbol{x}}}{\partial p_{\boldsymbol{i}}^2}\right)\right] = N\sum_{\boldsymbol{x}\in\mathscr{O}}\left[\frac{1}{P_{\boldsymbol{x}}}\left(\frac{\partial P_{\boldsymbol{x}}}{\partial p_{\boldsymbol{i}}}\right)^2 - \frac{\partial^2 P_{\boldsymbol{x}}}{\partial p_{\boldsymbol{i}}^2}\right]. \end{aligned}$$

Here,

$$\frac{\partial P_{\boldsymbol{x}}}{\partial p_{\boldsymbol{i}}} = \sum_{\boldsymbol{y}\in\mathscr{A}_{\boldsymbol{x}}} (-1)^{|\boldsymbol{x}|-|\boldsymbol{y}|}\frac{\partial}{\partial p_{\boldsymbol{i}}} G\Big(\sum_{\boldsymbol{h}\in A_{\boldsymbol{y}}} p_{\boldsymbol{h}}\Big),$$

and

$$\frac{\partial^2 P_{\boldsymbol{x}}}{\partial p_i^2} = \sum_{\boldsymbol{y}\in\mathscr{A}_{\boldsymbol{x}}} (-1)^{|\boldsymbol{x}|-|\boldsymbol{y}|}\frac{\partial^2}{\partial p_i^2} G\Big(\sum_{\boldsymbol{h}\in A_{\boldsymbol{y}}} p_{\boldsymbol{h}}\Big),$$

with

$$\sum_{\boldsymbol{x}\in\mathscr{O}} \frac{\partial^2 P_{\boldsymbol{x}}}{\partial p_i^2} = \frac{\partial^2}{\partial p_i^2}\sum_{\boldsymbol{x}\in\mathscr{O}} P_{\boldsymbol{x}} = \frac{\partial^2 1}{\partial p_i^2} = 0,$$

so that

$$\begin{aligned} \mathcal{I}_{p_{\boldsymbol{i}},p_{\boldsymbol{i}}} =& N\sum_{\boldsymbol{x}\in\mathscr{O}}\left[\left(\sum_{\boldsymbol{y}\in\mathscr{A}_{\boldsymbol{x}}}(-1)^{|\boldsymbol{x}|-|\boldsymbol{y}|} G\Big(\sum_{\boldsymbol{h}\in A_{\boldsymbol{y}}} p_{\boldsymbol{h}}\Big)\right)^{-1}\right. \\ & \left. \times \left(\sum_{\boldsymbol{y}\in\mathscr{A}_{\boldsymbol{x}}}(-1)^{|\boldsymbol{x}|-|\boldsymbol{y}|}\frac{\partial}{\partial p_{\boldsymbol{i}}} G\Big(\sum_{\boldsymbol{h}\in A_{\boldsymbol{y}}} p_{\boldsymbol{h}}\Big)\right)^2\right], \end{aligned} \tag{A.40a}$$

with $\frac{\partial}{\partial p_{\boldsymbol{i}}} G\Big(\sum_{\boldsymbol{h}\in A_{\boldsymbol{y}}} p_{\boldsymbol{h}}\Big)$ given by (A.39b).

Finally, the entries $\mathcal{I}_{p_{\boldsymbol{i}},p_{\boldsymbol{j}}}$ for $\boldsymbol{i} \neq \boldsymbol{j}$ are given by

$$\begin{aligned} \mathcal{I}_{p_{\boldsymbol{i}},p_{\boldsymbol{j}}} =& - \mathbb{E}\Big[\frac{\partial^2 L}{\partial p_{\boldsymbol{i}}\partial p_{\boldsymbol{j}}}(\boldsymbol{\theta})\Big] = -\mathbb{E}\left[\frac{\partial^2}{\partial p_{\boldsymbol{i}}\partial p_{\boldsymbol{j}}}\left(\sum_{\boldsymbol{x}\in\mathscr{O}} n_{\boldsymbol{x}} \log P_{\boldsymbol{x}}\right)\right] \\ =& - \sum_{\boldsymbol{x}\in\mathscr{O}}\left[\mathbb{E}n_{\boldsymbol{x}}\left(- \frac{1}{P_{\boldsymbol{x}}^2}\frac{\partial P_{\boldsymbol{x}}}{\partial p_{\boldsymbol{i}}}\frac{\partial P_{\boldsymbol{x}}}{\partial p_{\boldsymbol{j}}} + \frac{1}{P_{\boldsymbol{x}}}\frac{\partial^2 P_{\boldsymbol{x}}}{\partial p_{\boldsymbol{i}}\partial p_{\boldsymbol{j}}}\right)\right] \\ =& N\sum_{\boldsymbol{x}\in\mathscr{O}}\left[\frac{1}{P_{\boldsymbol{x}}}\frac{\partial P_{\boldsymbol{x}}}{\partial p_{\boldsymbol{i}}}\frac{\partial P_{\boldsymbol{x}}}{\partial p_{\boldsymbol{j}}} - \frac{\partial^2 P_{\boldsymbol{x}}}{\partial p_{\boldsymbol{i}}\partial p_{\boldsymbol{j}}}\right]. \end{aligned}$$

From the expression of $\frac{\partial P_{\boldsymbol{x}}}{\partial p_{\boldsymbol{i}}}$ given above, one derives

$$\frac{\partial^2 P_{\boldsymbol{x}}}{\partial p_{\boldsymbol{i}}\partial p_{\boldsymbol{j}}} = \sum_{\boldsymbol{y}\in\mathscr{A}_{\boldsymbol{x}}} (-1)^{|\boldsymbol{x}|-|\boldsymbol{y}|}\frac{\partial^2}{\partial p_{\boldsymbol{i}}\partial p_{\boldsymbol{j}}} G\Big(\sum_{\boldsymbol{h}\in A_{\boldsymbol{y}}} p_{\boldsymbol{h}}\Big),$$

with

$$\sum_{\boldsymbol{x}\in\mathscr{O}} \frac{\partial^2 P_{\boldsymbol{x}}}{\partial p_{\boldsymbol{i}}\partial p_{\boldsymbol{j}}} = \frac{\partial^2}{\partial p_{\boldsymbol{i}}\partial p_{\boldsymbol{j}}} \sum_{\boldsymbol{x}\in\mathscr{O}} P_{\boldsymbol{x}} = \frac{\partial^2 1}{\partial p_{\boldsymbol{i}}\partial p_{\boldsymbol{j}}} = 0.$$

Thus,

$$\begin{aligned}\mathcal{I}_{p_{\boldsymbol{i}},p_{\boldsymbol{j}}} = N \sum_{\boldsymbol{x}\in\mathscr{O}} \Bigg[\Big(\sum_{\boldsymbol{y}\in\mathscr{A}_{\boldsymbol{x}}} (-1)^{|\boldsymbol{x}|-|\boldsymbol{y}|} G\Big(\sum_{\boldsymbol{h}\in A_{\boldsymbol{y}}} p_{\boldsymbol{h}} \Big) \Big)^{-1} \\ \times \Big(\sum_{\boldsymbol{y}\in\mathscr{A}_{\boldsymbol{x}}} (-1)^{|\boldsymbol{x}|-|\boldsymbol{y}|} \frac{\partial}{\partial p_{\boldsymbol{i}}} G\Big(\sum_{\boldsymbol{h}\in A_{\boldsymbol{y}}} p_{\boldsymbol{h}} \Big) \Big) \\ \times \Big(\sum_{\boldsymbol{y}\in\mathscr{A}_{\boldsymbol{x}}} (-1)^{|\boldsymbol{x}|-|\boldsymbol{y}|} \frac{\partial}{\partial p_{\boldsymbol{j}}} G\Big(\sum_{\boldsymbol{h}\in A_{\boldsymbol{y}}} p_{\boldsymbol{h}} \Big) \Big) \Bigg].\end{aligned} \tag{A.41a}$$

Although the entries of the information matrix are now derived and can be either used in (A.18) or (A.28), the summations are combinatorial rather involved as one has to sum over all possible observations.

The Fisher information is usually defined as the product of the score function, and only coincides with the negative expectation of the Hessian matrix if the before-mentioned regulatory conditions hold. Sometimes, it is easier to calculate the Fisher information from the product of the score function. This is explored next.

Derivation of the information matrix from the score function

Consider the log-likelihood function $l(\boldsymbol{\theta}')$. The Fisher information is defined from the score function by

$$\mathcal{S}_{i,j} \coloneqq \mathbb{E}\left[\frac{\partial l(\boldsymbol{\theta}')}{\partial \theta'_i} \frac{\partial l(\boldsymbol{\theta}')}{\partial \theta'_j} \right]. \tag{A.42}$$

This definition of the Fisher information is equivalent to $\mathcal{I}'(\boldsymbol{\theta}')$ given in (A.28) under the following three regulatory conditions (cf. [4], section 2.3): (i) the partial derivatives of l with respect to all parameters exist almost everywhere (with respect to the set of possible parameters), (ii) integration and differentiation can be interchanged for the density underlying the likelihood function, and (iii) the support of the density function does not depend on the parameter choice. In the present case, these conditions clearly hold.

Note that, this argument is not valid when introducing a Lagrange multiplier in the likelihood function as a nuisance parameter, because the resulting function $\Lambda(\tilde{\boldsymbol{\theta}})$ is not a likelihood function. The Fisher information cannot be defined from the score function in this case. In fact, this would lead to a singular matrix for any admissible parameter,

implying that necessarily

$$\mathbb{E}\left[\frac{\partial\Lambda(\tilde{\boldsymbol{\theta}})}{\partial\tilde{\theta}_i}\frac{\partial\Lambda(\tilde{\boldsymbol{\theta}})}{\partial\tilde{\theta}_j}\right] \neq -\mathbb{E}\left[\frac{\partial^2\Lambda(\tilde{\boldsymbol{\theta}})}{\partial\tilde{\theta}_i\partial\tilde{\theta}_j}\right].$$

Remember, the corresponding entries of $\tilde{\mathcal{I}}^{-1}(\tilde{\boldsymbol{\theta}})$ and $\mathcal{I}'^{-1}(\boldsymbol{\theta}')$ are identical, not those of $\tilde{\mathcal{I}}(\tilde{\boldsymbol{\theta}})$ and $\mathcal{I}'(\boldsymbol{\theta}')$.

Thus, to derive the Fisher information from the score function, one has to eliminate a redundant parameter. To clarify the point, the equivalence between the Fisher information derived from the product of the score function and the negative Hessian matrix is shown directly in what follows.

As mentioned above, one parameter needs to be eliminated first. While any of the H possible haplotype frequencies could be expressed by the others, here, the frequency of the H-th haplotype denoted $\boldsymbol{h}_H$ is chosen to be expressed as $p_H = 1 - \sum_{k=1}^{H-1} p_k$. Since this substitution eliminates the redundant parameter p_H, it is convenient to rewrite the observation space $\mathscr{O}$ as the disjoint union of all observations compatible with haplotype $\boldsymbol{h}_H$,

$$\mathscr{O}_1 \coloneqq \left\{\boldsymbol{x} \in \mathscr{O} \,|\, \boldsymbol{h}_H^{(k)} \in x_k \text{ for all } k \in \{1,2,\ldots,L\}\right\},$$

and the set $\mathscr{O}_2 \coloneqq \mathscr{O} \setminus \mathscr{O}_1$ of all observations not compatible with $\boldsymbol{h}_H$. Hence, the log-likelihood function $l(\boldsymbol{\theta}')$ in the reduced space Θ' is redefined from (53) as

$$\begin{aligned} l(\boldsymbol{\theta}') = L(\boldsymbol{\theta}) = \sum_{\boldsymbol{x}\in\mathscr{O}_1} n_{\boldsymbol{x}} \log\Bigg(&\sum_{\boldsymbol{y}\in\mathscr{A}_{\boldsymbol{x}}^{(1)}} (-1)^{|\boldsymbol{x}|-|\boldsymbol{y}|} G\Big(\sum_{\boldsymbol{h}\in A_{\boldsymbol{y}}} p_{\boldsymbol{h}}\Big) \\ &+ \sum_{\boldsymbol{y}\in\mathscr{A}_{\boldsymbol{x}}^{(2)}} (-1)^{|\boldsymbol{x}|-|\boldsymbol{y}|} G\Big(\sum_{\boldsymbol{h}\in A_{\boldsymbol{y}}} p_{\boldsymbol{h}}\Big)\Bigg) \\ + \sum_{\boldsymbol{x}\in\mathscr{O}_2} n_{\boldsymbol{x}} \log\Bigg(&\sum_{\boldsymbol{y}\in\mathscr{A}_{\boldsymbol{x}}^{(2)}} (-1)^{|\boldsymbol{x}|-|\boldsymbol{y}|} G\Big(\sum_{\boldsymbol{h}\in A_{\boldsymbol{y}}} p_{\boldsymbol{h}}\Big)\Bigg), \end{aligned} \tag{A.43}$$

where $\mathscr{A}_{\boldsymbol{x}}^{(1)} := \mathscr{A}_{\boldsymbol{x}} \cap \mathscr{O}_1$ and $\mathscr{A}_{\boldsymbol{x}}^{(2)} := \mathscr{A}_{\boldsymbol{x}} \cap \mathscr{O}_2$. Note that, for all $\boldsymbol{y} \in \mathscr{A}_{\boldsymbol{x}}^{(1)}$,

$$\sum_{\boldsymbol{h}\in A_{\boldsymbol{y}}} p_{\boldsymbol{h}} = \sum_{\substack{\boldsymbol{h}\in A_{\boldsymbol{y}}\\ \boldsymbol{h}\neq\boldsymbol{h}_H}} p_{\boldsymbol{h}} + p_H = \sum_{\substack{\boldsymbol{h}\in A_{\boldsymbol{y}}\\ \boldsymbol{h}\neq\boldsymbol{h}_H}} p_{\boldsymbol{h}} + 1 - \sum_{\substack{\boldsymbol{h}\in \mathscr{H}\\ \boldsymbol{h}\neq\boldsymbol{h}_H}} p_{\boldsymbol{h}} = 1 - \sum_{\substack{\boldsymbol{h}\notin A_{\boldsymbol{y}}\\ \boldsymbol{h}\neq\boldsymbol{h}_H}} p_{\boldsymbol{h}}.$$

Therefore, the log-likelihood function becomes

$$\begin{aligned} l(\boldsymbol{\theta}') = \sum_{\boldsymbol{x}\in\mathscr{O}_1} n_{\boldsymbol{x}} \log\Bigg(&\sum_{\boldsymbol{y}\in\mathscr{A}_{\boldsymbol{x}}^{(1)}} (-1)^{|\boldsymbol{x}|-|\boldsymbol{y}|} G\Big(1 - \sum_{\substack{\boldsymbol{h}\notin A_{\boldsymbol{y}}\\ \boldsymbol{h}\neq\boldsymbol{h}_H}} p_{\boldsymbol{h}}\Big) \\ &+ \sum_{\boldsymbol{y}\in\mathscr{A}_{\boldsymbol{x}}^{(2)}} (-1)^{|\boldsymbol{x}|-|\boldsymbol{y}|} G\Big(\sum_{\boldsymbol{h}\in A_{\boldsymbol{y}}} p_{\boldsymbol{h}}\Big)\Bigg) \\ + \sum_{\boldsymbol{x}\in\mathscr{O}_2} n_{\boldsymbol{x}} \log\Bigg(&\sum_{\boldsymbol{y}\in\mathscr{A}_{\boldsymbol{x}}^{(2)}} (-1)^{|\boldsymbol{x}|-|\boldsymbol{y}|} G\Big(\sum_{\boldsymbol{h}\in A_{\boldsymbol{y}}} p_{\boldsymbol{h}}\Big)\Bigg). \end{aligned}$$

To simplify the notation, let

$$P_{\boldsymbol{x}}^{(1)} \coloneqq \sum_{\boldsymbol{y}\in\mathscr{A}_{\boldsymbol{x}}^{(1)}} (-1)^{|\boldsymbol{x}|-|\boldsymbol{y}|} G\Bigg(1 - \sum_{\substack{\boldsymbol{h}\notin A_{\boldsymbol{y}}\\ \boldsymbol{h}\neq\boldsymbol{h}_H}} p_{\boldsymbol{h}}\Bigg), \tag{A.44a}$$

and

$$P_{\boldsymbol{x}}^{(2)} \coloneqq \sum_{\boldsymbol{y}\in\mathscr{A}_{\boldsymbol{x}}^{(2)}} (-1)^{|\boldsymbol{x}|-|\boldsymbol{y}|} G\Bigg(\sum_{\boldsymbol{h}\in A_{\boldsymbol{y}}} p_{\boldsymbol{h}}\Bigg). \tag{A.44b}$$

Note that, $\mathscr{A}_{\boldsymbol{x}}^{(1)} = \emptyset$ for all $\boldsymbol{x} \in \mathscr{O}_2$. Hence, $P_{\boldsymbol{x}}^{(1)} = 0$ for all $\boldsymbol{x} \in \mathscr{O}_2$. Therefore, the likelihood function is rewritten as

$$l(\boldsymbol{\theta}') = \sum_{\boldsymbol{x}\in\mathscr{O}_1} n_{\boldsymbol{x}} \log\big(P_{\boldsymbol{x}}^{(1)} + P_{\boldsymbol{x}}^{(2)}\big) + \sum_{\boldsymbol{x}\in\mathscr{O}_2} n_{\boldsymbol{x}} \log P_{\boldsymbol{x}}^{(2)}. \tag{A.44c}$$

The entries of the Fisher information are derived from (A.42) using the definition of the likelihood function in (A.44c). In particular, for $\theta_i' = \theta_j' = \lambda$, one obtains

$$\begin{aligned}
\mathcal{S}_{\lambda,\lambda} =& \mathbb{E}\left[\left(\frac{\partial l(\boldsymbol{\theta}')}{\partial\lambda}\right)^2\right] = \mathbb{E}\left[\left(\sum_{\boldsymbol{x}\in\mathscr{O}_1} n_{\boldsymbol{x}} \frac{\partial}{\partial\lambda}\log\big(P_{\boldsymbol{x}}^{(1)} + P_{\boldsymbol{x}}^{(2)}\big) + \sum_{\boldsymbol{x}\in\mathscr{O}_2} n_{\boldsymbol{x}} \frac{\partial}{\partial\lambda}\log P_{\boldsymbol{x}}^{(2)}\right)^2\right]\\
=&\mathbb{E}\left[\left(\sum_{\boldsymbol{x}\in\mathscr{O}_1} n_{\boldsymbol{x}} \frac{1}{P_{\boldsymbol{x}}^{(1)} + P_{\boldsymbol{x}}^{(2)}} \frac{\partial}{\partial\lambda}\big(P_{\boldsymbol{x}}^{(1)} + P_{\boldsymbol{x}}^{(2)}\big) + \sum_{\boldsymbol{x}\in\mathscr{O}_2} n_{\boldsymbol{x}} \frac{1}{P_{\boldsymbol{x}}^{(2)}} \frac{\partial}{\partial\lambda} P_{\boldsymbol{x}}^{(2)}\right)^2\right]\\
=&\mathbb{E}\left[\left(\sum_{\boldsymbol{x}\in\mathscr{O}_1} n_{\boldsymbol{x}} S_{\boldsymbol{x}}^{(1,2)} + \sum_{\boldsymbol{x}\in\mathscr{O}_2} n_{\boldsymbol{x}} S_{\boldsymbol{x}}^{(2)}\right)^2\right],
\end{aligned}$$

where $S_{\boldsymbol{x}}^{(1,2)} \coloneqq \frac{1}{P_{\boldsymbol{x}}^{(1)}+P_{\boldsymbol{x}}^{(2)}} \frac{\partial}{\partial\lambda}\big(P_{\boldsymbol{x}}^{(1)} + P_{\boldsymbol{x}}^{(2)}\big)$, and $S_{\boldsymbol{x}}^{(2)} \coloneqq \frac{1}{P_{\boldsymbol{x}}^{(2)}} \frac{\partial}{\partial\lambda} P_{\boldsymbol{x}}^{(2)}$. Therefore,

$$\begin{aligned}
\mathcal{S}_{\lambda,\lambda} =& \mathbb{E}\left[\left(\sum_{\boldsymbol{x}\in\mathscr{O}_1} n_{\boldsymbol{x}} S_{\boldsymbol{x}}^{(1,2)}\right)^2 + 2\sum_{\boldsymbol{x}\in\mathscr{O}_1} n_{\boldsymbol{x}} S_{\boldsymbol{x}}^{(1,2)} \sum_{\boldsymbol{x}\in\mathscr{O}_2} n_{\boldsymbol{x}} S_{\boldsymbol{x}}^{(2)} + \left(\sum_{\boldsymbol{x}\in\mathscr{O}_2} n_{\boldsymbol{x}} S_{\boldsymbol{x}}^{(2)}\right)^2\right]\\
=&\mathbb{E}\Bigg[\sum_{\boldsymbol{x}\in\mathscr{O}_1}\sum_{\boldsymbol{y}\in\mathscr{O}_1} n_{\boldsymbol{x}} n_{\boldsymbol{y}} S_{\boldsymbol{x}}^{(1,2)} S_{\boldsymbol{y}}^{(1,2)} + 2\sum_{\boldsymbol{x}\in\mathscr{O}_1}\sum_{\boldsymbol{y}\in\mathscr{O}_2} n_{\boldsymbol{x}} n_{\boldsymbol{y}} S_{\boldsymbol{x}}^{(1,2)} S_{\boldsymbol{y}}^{(2)}\\
&+ \sum_{\boldsymbol{x}\in\mathscr{O}_2}\sum_{\boldsymbol{y}\in\mathscr{O}_2} n_{\boldsymbol{x}} n_{\boldsymbol{y}} S_{\boldsymbol{x}}^{(2)} S_{\boldsymbol{y}}^{(2)}\Bigg]\\
=& \sum_{\boldsymbol{x}\in\mathscr{O}_1}\sum_{\boldsymbol{y}\in\mathscr{O}_1} \mathbb{E}\big[n_{\boldsymbol{x}} n_{\boldsymbol{y}}\big] S_{\boldsymbol{x}}^{(1,2)} S_{\boldsymbol{y}}^{(1,2)} + 2\sum_{\boldsymbol{x}\in\mathscr{O}_1}\sum_{\boldsymbol{y}\in\mathscr{O}_2} \mathbb{E}\big[n_{\boldsymbol{x}} n_{\boldsymbol{y}}\big] S_{\boldsymbol{x}}^{(1,2)} S_{\boldsymbol{y}}^{(2)}\\
&+ \sum_{\boldsymbol{x}\in\mathscr{O}_2}\sum_{\boldsymbol{y}\in\mathscr{O}_2} \mathbb{E}\big[n_{\boldsymbol{x}} n_{\boldsymbol{y}}\big] S_{\boldsymbol{x}}^{(2)} S_{\boldsymbol{y}}^{(2)}.
\end{aligned}$$

Recall that $n_{\boldsymbol{x}}$ follows a multinomial distribution with parameters N and $P_{\boldsymbol{x}}$. Hence, $\mathbb{E}\big[n_{\boldsymbol{x}}\big] = NP_{\boldsymbol{x}}$, and $\mathrm{Cov}(n_{\boldsymbol{x}}, n_{\boldsymbol{y}}) = \mathbb{E}\big[n_{\boldsymbol{x}} n_{\boldsymbol{y}}\big] - \mathbb{E}\big[n_{\boldsymbol{x}}\big]\mathbb{E}\big[n_{\boldsymbol{y}}\big] = -NP_{\boldsymbol{x}}P_{\boldsymbol{y}}$ for $\boldsymbol{x} \neq \boldsymbol{y}$, and $\mathrm{Cov}(n_{\boldsymbol{x}}, n_{\boldsymbol{x}}) = \mathbb{E}\big[n_{\boldsymbol{x}}^2\big] - \mathbb{E}\big[n_{\boldsymbol{x}}\big]^2 = \mathrm{Var}(n_{\boldsymbol{x}}) = NP_{\boldsymbol{x}}(1 - P_{\boldsymbol{x}})$, which yields

$$\mathbb{E}\big[n_{\boldsymbol{x}}^2\big] = NP_{\boldsymbol{x}}(1 - P_{\boldsymbol{x}}) + (NP_{\boldsymbol{x}})^2 = N^2P_{\boldsymbol{x}}^2 - NP_{\boldsymbol{x}}^2 + NP_{\boldsymbol{x}}. \tag{A.47a}$$

Moreover, for $\boldsymbol{x} \neq \boldsymbol{y}$, $\operatorname{Cov}(n_{\boldsymbol{x}}, n_{\boldsymbol{y}}) = \mathbb{E}\big[n_{\boldsymbol{x}} n_{\boldsymbol{y}}\big] - N^2 P_{\boldsymbol{x}} P_{\boldsymbol{y}} = -N P_{\boldsymbol{x}} P_{\boldsymbol{y}}$, and

$$\mathbb{E}\big[n_{\boldsymbol{x}} n_{\boldsymbol{y}}\big] = -N P_{\boldsymbol{x}} P_{\boldsymbol{y}} + N^2 P_{\boldsymbol{x}} P_{\boldsymbol{y}} = N(N-1) P_{\boldsymbol{x}} P_{\boldsymbol{y}}. \tag{A.47b}$$

Thus,

$$\begin{aligned}
\mathcal{S}_{\lambda,\lambda} = {} & \sum_{\boldsymbol{x}\in\mathscr{O}_1} S_{\boldsymbol{x}}^{2(1,2)} \big(N^2 P_{\boldsymbol{x}}^2 - N P_{\boldsymbol{x}}^2 + N P_{\boldsymbol{x}}\big) + N(N-1) \sum_{\boldsymbol{x}\in\mathscr{O}_1} \sum_{\substack{\boldsymbol{y}\in\mathscr{O}_1\\ \boldsymbol{y}\neq\boldsymbol{x}}} S_{\boldsymbol{x}}^{(1,2)} S_{\boldsymbol{y}}^{(1,2)} P_{\boldsymbol{x}} P_{\boldsymbol{y}} \\
& + 2N(N-1) \sum_{\boldsymbol{x}\in\mathscr{O}_1} \sum_{\boldsymbol{y}\in\mathscr{O}_2} S_{\boldsymbol{x}}^{(1,2)} S_{\boldsymbol{y}}^{(2)} P_{\boldsymbol{x}} P_{\boldsymbol{y}} \\
& + \sum_{\boldsymbol{x}\in\mathscr{O}_2} S_{\boldsymbol{x}}^{2(2)} \big(N^2 P_{\boldsymbol{x}}^2 - N P_{\boldsymbol{x}}^2 + N P_{\boldsymbol{x}}\big) + N(N-1) \sum_{\boldsymbol{x}\in\mathscr{O}_2} \sum_{\substack{\boldsymbol{y}\in\mathscr{O}_2\\ \boldsymbol{y}\neq\boldsymbol{x}}} S_{\boldsymbol{x}}^{(2)} S_{\boldsymbol{y}}^{(2)} P_{\boldsymbol{x}} P_{\boldsymbol{y}} \\
= {} & \sum_{\boldsymbol{x}\in\mathscr{O}_1} S_{\boldsymbol{x}}^{2(1,2)} \big(N^2 P_{\boldsymbol{x}}^2 - N P_{\boldsymbol{x}}^2 + N P_{\boldsymbol{x}}\big) + N(N-1) \sum_{\boldsymbol{x}\in\mathscr{O}_1} S_{\boldsymbol{x}}^{(1,2)} P_{\boldsymbol{x}} \sum_{\substack{\boldsymbol{y}\in\mathscr{O}_1\\ \boldsymbol{y}\neq\boldsymbol{x}}} S_{\boldsymbol{y}}^{(1,2)} P_{\boldsymbol{y}} \\
& + 2N(N-1) \sum_{\boldsymbol{x}\in\mathscr{O}_1} S_{\boldsymbol{x}}^{(1,2)} P_{\boldsymbol{x}} \sum_{\boldsymbol{y}\in\mathscr{O}_2} S_{\boldsymbol{y}}^{(2)} P_{\boldsymbol{y}} \\
& \sum_{\boldsymbol{x}\in\mathscr{O}_2} S_{\boldsymbol{x}}^{2(2)} \big(N^2 P_{\boldsymbol{x}}^2 - N P_{\boldsymbol{x}}^2 + N P_{\boldsymbol{x}}\big) + N(N-1) \sum_{\boldsymbol{x}\in\mathscr{O}_2} S_{\boldsymbol{x}}^{(2)} P_{\boldsymbol{x}} \sum_{\substack{\boldsymbol{y}\in\mathscr{O}_2\\ \boldsymbol{y}\neq\boldsymbol{x}}} S_{\boldsymbol{y}}^{(2)} P_{\boldsymbol{y}}.
\end{aligned}$$

Using the definitions of $S_{\boldsymbol{x}}^{(1,2)}$ and $S_{\boldsymbol{x}}^{(2)}$ yields

$$\begin{aligned}
\mathcal{S}_{\lambda,\lambda} = {} & \sum_{\boldsymbol{x}\in\mathscr{O}_1} \left(\frac{P_{\boldsymbol{x}}}{P_{\boldsymbol{x}}^{(1)} + P_{\boldsymbol{x}}^{(2)}} \frac{\partial}{\partial\lambda} \big(P_{\boldsymbol{x}}^{(1)} + P_{\boldsymbol{x}}^{(2)}\big) \right)^2 \big(N^2 - N + N P_{\boldsymbol{x}}^{-1}\big) \\
& + N(N-1) \sum_{\boldsymbol{x}\in\mathscr{O}_1} \frac{P_{\boldsymbol{x}}}{P_{\boldsymbol{x}}^{(1)} + P_{\boldsymbol{x}}^{(2)}} \frac{\partial}{\partial\lambda} \big(P_{\boldsymbol{x}}^{(1)} + P_{\boldsymbol{x}}^{(2)}\big) \sum_{\substack{\boldsymbol{y}\in\mathscr{O}_1\\ \boldsymbol{y}\neq\boldsymbol{x}}} \frac{P_{\boldsymbol{y}}}{P_{\boldsymbol{y}}^{(1)} + P_{\boldsymbol{y}}^{(2)}} \frac{\partial}{\partial\lambda} \big(P_{\boldsymbol{y}}^{(1)} + P_{\boldsymbol{y}}^{(2)}\big) \\
& + 2N(N-1) \sum_{\boldsymbol{x}\in\mathscr{O}_1} \frac{P_{\boldsymbol{x}}}{P_{\boldsymbol{x}}^{(1)} + P_{\boldsymbol{x}}^{(2)}} \frac{\partial}{\partial\lambda} \big(P_{\boldsymbol{x}}^{(1)} + P_{\boldsymbol{x}}^{(2)}\big) \sum_{\boldsymbol{y}\in\mathscr{O}_2} \frac{P_{\boldsymbol{y}}}{P_{\boldsymbol{y}}^{(2)}} \frac{\partial}{\partial\lambda} P_{\boldsymbol{y}}^{(2)} \\
& + N(N-1) \sum_{\boldsymbol{x}\in\mathscr{O}_2} \frac{P_{\boldsymbol{x}}}{P_{\boldsymbol{x}}^{(2)}} \frac{\partial}{\partial\lambda} P_{\boldsymbol{x}}^{(2)} \sum_{\substack{\boldsymbol{y}\in\mathscr{O}_2\\ \boldsymbol{y}\neq\boldsymbol{x}}} \frac{P_{\boldsymbol{y}}}{P_{\boldsymbol{y}}^{(2)}} \frac{\partial}{\partial\lambda} P_{\boldsymbol{y}}^{(2)} \\
& + \sum_{\boldsymbol{x}\in\mathscr{O}_2} \left(\frac{P_{\boldsymbol{x}}}{P_{\boldsymbol{x}}^{(2)}} \frac{\partial}{\partial\lambda} P_{\boldsymbol{x}}^{(2)} \right)^2 \big(N^2 - N + N P_{\boldsymbol{x}}^{-1}\big),
\end{aligned}$$

Recall that, $P_{\boldsymbol{x}} = P_{\boldsymbol{x}}^{(1)} + P_{\boldsymbol{x}}^{(2)}$. Therefore,

$$
\begin{aligned}
\mathcal{S}_{\lambda,\lambda} = &\sum_{\boldsymbol{x}\in\mathscr{O}_1} \left(\frac{\partial}{\partial\lambda} P_{\boldsymbol{x}}\right)^2 \left(N^2 - N + NP_{\boldsymbol{x}}^{-1}\right) \\
&+ N(N-1) \sum_{\boldsymbol{x}\in\mathscr{O}_1} \frac{\partial}{\partial\lambda} P_{\boldsymbol{x}} \sum_{\substack{\boldsymbol{y}\in\mathscr{O}_1 \\ \boldsymbol{y}\neq\boldsymbol{x}}} \frac{\partial}{\partial\lambda} P_{\boldsymbol{y}} \\
&+ 2N(N-1) \sum_{\boldsymbol{x}\in\mathscr{O}_1} \frac{\partial}{\partial\lambda} P_{\boldsymbol{x}} \sum_{\boldsymbol{y}\in\mathscr{O}_2} \frac{P_{\boldsymbol{y}}}{P_{\boldsymbol{y}}^{(2)}} \frac{\partial}{\partial\lambda} P_{\boldsymbol{y}}^{(2)} \\
&+ N(N-1) \sum_{\boldsymbol{x}\in\mathscr{O}_2} \frac{P_{\boldsymbol{x}}}{P_{\boldsymbol{x}}^{(2)}} \frac{\partial}{\partial\lambda} P_{\boldsymbol{x}}^{(2)} \sum_{\substack{\boldsymbol{y}\in\mathscr{O}_2 \\ \boldsymbol{y}\neq\boldsymbol{x}}} \frac{P_{\boldsymbol{y}}}{P_{\boldsymbol{y}}^{(2)}} \frac{\partial}{\partial\lambda} P_{\boldsymbol{y}}^{(2)} \\
&+ \sum_{\boldsymbol{x}\in\mathscr{O}_2} \left(\frac{P_{\boldsymbol{x}}}{P_{\boldsymbol{x}}^{(2)}} \frac{\partial}{\partial\lambda} P_{\boldsymbol{x}}^{(2)}\right)^2 \left(N^2 - N + NP_{\boldsymbol{x}}^{-1}\right),
\end{aligned}
$$

Moreover, $\dfrac{P_{\boldsymbol{x}}}{P_{\boldsymbol{x}}^{(2)}} = \dfrac{P_{\boldsymbol{x}}^{(1)} + P_{\boldsymbol{x}}^{(2)}}{P_{\boldsymbol{x}}^{(2)}} = \dfrac{P_{\boldsymbol{x}}^{(2)}}{P_{\boldsymbol{x}}^{(2)}} = 1$ for all $\boldsymbol{x} \in \mathscr{O}_2$. Thus,

$$
\begin{aligned}
\mathcal{S}_{\lambda,\lambda} = &\sum_{\boldsymbol{x}\in\mathscr{O}_1} \left(\frac{\partial}{\partial\lambda} P_{\boldsymbol{x}}\right)^2 \left(N^2 - N + NP_{\boldsymbol{x}}^{-1}\right) + N(N-1) \sum_{\boldsymbol{x}\in\mathscr{O}_1} \frac{\partial}{\partial\lambda} P_{\boldsymbol{x}} \sum_{\substack{\boldsymbol{y}\in\mathscr{O}_1 \\ \boldsymbol{y}\neq\boldsymbol{x}}} \frac{\partial}{\partial\lambda} P_{\boldsymbol{y}} \\
&+ 2N(N-1) \sum_{\boldsymbol{x}\in\mathscr{O}_1} \frac{\partial}{\partial\lambda} P_{\boldsymbol{x}} \sum_{\boldsymbol{y}\in\mathscr{O}_2} \frac{\partial}{\partial\lambda} P_{\boldsymbol{y}}^{(2)} + N(N-1) \sum_{\boldsymbol{x}\in\mathscr{O}_2} \frac{\partial}{\partial\lambda} P_{\boldsymbol{x}}^{(2)} \sum_{\substack{\boldsymbol{y}\in\mathscr{O}_2 \\ \boldsymbol{y}\neq\boldsymbol{x}}} \frac{\partial}{\partial\lambda} P_{\boldsymbol{y}}^{(2)} \\
&+ \sum_{\boldsymbol{x}\in\mathscr{O}_2} \left(\frac{\partial}{\partial\lambda} P_{\boldsymbol{x}}^{(2)}\right)^2 \left(N^2 - N + NP_{\boldsymbol{x}}^{-1}\right) \\
= &N \sum_{\boldsymbol{x}\in\mathscr{O}_1} P_{\boldsymbol{x}}^{-1} \left(\frac{\partial}{\partial\lambda} P_{\boldsymbol{x}}\right)^2 + N(N-1) \sum_{\boldsymbol{x}\in\mathscr{O}_1} \left(\frac{\partial}{\partial\lambda} P_{\boldsymbol{x}}\right)^2 \\
&+ N(N-1) \sum_{\boldsymbol{x}\in\mathscr{O}_1} \frac{\partial}{\partial\lambda} P_{\boldsymbol{x}} \sum_{\substack{\boldsymbol{y}\in\mathscr{O}_1 \\ \boldsymbol{y}\neq\boldsymbol{x}}} \frac{\partial}{\partial\lambda} P_{\boldsymbol{y}} + 2N(N-1) \sum_{\boldsymbol{x}\in\mathscr{O}_1} \frac{\partial}{\partial\lambda} P_{\boldsymbol{x}} \sum_{\boldsymbol{y}\in\mathscr{O}_2} \frac{\partial}{\partial\lambda} P_{\boldsymbol{y}}^{(2)} \\
&+ N(N-1) \sum_{\boldsymbol{x}\in\mathscr{O}_2} \frac{\partial}{\partial\lambda} P_{\boldsymbol{x}}^{(2)} \sum_{\substack{\boldsymbol{y}\in\mathscr{O}_2 \\ \boldsymbol{y}\neq\boldsymbol{x}}} \frac{\partial}{\partial\lambda} P_{\boldsymbol{y}}^{(2)} + N(N-1) \sum_{\boldsymbol{x}\in\mathscr{O}_2} \left(\frac{\partial}{\partial\lambda} P_{\boldsymbol{x}}^{(2)}\right)^2 \\
&+ N \sum_{\boldsymbol{x}\in\mathscr{O}_2} P_{\boldsymbol{x}}^{-1} \left(\frac{\partial}{\partial\lambda} P_{\boldsymbol{x}}^{(2)}\right)^2.
\end{aligned}
$$

Note that, the sum of the second and third terms becomes

$$
N(N-1) \sum_{\boldsymbol{x}\in\mathscr{O}_1} \frac{\partial}{\partial\lambda} P_{\boldsymbol{x}} \sum_{\boldsymbol{y}\in\mathscr{O}_1} \frac{\partial}{\partial\lambda} P_{\boldsymbol{y}}.
$$

When adding the fifth and sixth terms, the above equality also holds for $\boldsymbol{x} \in \mathscr{O}_2$. This

yields

$$\begin{aligned}\mathcal{S}_{\lambda,\lambda} =& N\sum_{\boldsymbol{x}\in\mathscr{O}_1} P_{\boldsymbol{x}}^{-1}\left(\frac{\partial}{\partial\lambda}P_{\boldsymbol{x}}\right)^2 + N(N-1)\sum_{\boldsymbol{x}\in\mathscr{O}_1}\frac{\partial}{\partial\lambda}P_{\boldsymbol{x}}\sum_{\boldsymbol{y}\in\mathscr{O}_1}\frac{\partial}{\partial\lambda}P_{\boldsymbol{y}} \\ &+ 2N(N-1)\sum_{\boldsymbol{x}\in\mathscr{O}_1}\frac{\partial}{\partial\lambda}P_{\boldsymbol{x}}\sum_{\boldsymbol{y}\in\mathscr{O}_2}\frac{\partial}{\partial\lambda}P_{\boldsymbol{y}}^{(2)} + N(N-1)\sum_{\boldsymbol{x}\in\mathscr{O}_2}\frac{\partial}{\partial\lambda}P_{\boldsymbol{x}}^{(2)}\sum_{\boldsymbol{y}\in\mathscr{O}_2}\frac{\partial}{\partial\lambda}P_{\boldsymbol{y}}^{(2)} \\ &+ N\sum_{\boldsymbol{x}\in\mathscr{O}_2} P_{\boldsymbol{x}}^{-1}\left(\frac{\partial}{\partial\lambda}P_{\boldsymbol{x}}^{(2)}\right)^2.\end{aligned}$$

Since $P_{\boldsymbol{x}} = P_{\boldsymbol{x}}^{(1)} + P_{\boldsymbol{x}}^{(2)} = P_{\boldsymbol{x}}^{(2)}$ for all $\boldsymbol{x}\in\mathscr{O}_2$, adding the second, third, and fourth terms yield

$$N(N-1)\left(\sum_{\boldsymbol{x}\in\mathscr{O}_1}\frac{\partial}{\partial\lambda}P_{\boldsymbol{x}} + \sum_{\boldsymbol{x}\in\mathscr{O}_2}\frac{\partial}{\partial\lambda}P_{\boldsymbol{x}}\right)^2 = N(N-1)\left(\sum_{\boldsymbol{x}\in\mathscr{O}}\frac{\partial}{\partial\lambda}P_{\boldsymbol{x}}\right)^2 = 0.$$

Therefore,

$$\mathcal{S}_{\lambda,\lambda} = N\sum_{\boldsymbol{x}\in\mathscr{O}_1} P_{\boldsymbol{x}}^{-1}\left(\frac{\partial}{\partial\lambda}P_{\boldsymbol{x}}\right)^2 + N\sum_{\boldsymbol{x}\in\mathscr{O}_2} P_{\boldsymbol{x}}^{-1}\left(\frac{\partial}{\partial\lambda}P_{\boldsymbol{x}}^{(2)}\right)^2.$$

This sum becomes

$$\mathcal{S}_{\lambda,\lambda} = N\sum_{\boldsymbol{x}\in\mathscr{O}} P_{\boldsymbol{x}}^{-1}\left(\frac{\partial}{\partial\lambda}P_{\boldsymbol{x}}\right)^2 = \mathcal{I}_{\lambda,\lambda} \tag{A.48a}$$

by comparison with (A.38a).

Next, for $\theta_i' = \lambda$ and $\theta_j' = p_{\boldsymbol{i}}$,

$$\mathcal{S}_{\lambda,p_{\boldsymbol{i}}} = \mathbb{E}\left[\frac{\partial l(\boldsymbol{\theta}')}{\partial\lambda}\frac{\partial l(\boldsymbol{\theta}')}{\partial p_{\boldsymbol{i}}}\right].$$

Following the same approach as above, one obtains

$$
\begin{aligned}
\mathcal{S}_{\lambda,p_{\boldsymbol{i}}} = &\sum_{\boldsymbol{x}\in\mathscr{O}_1} \frac{P_{\boldsymbol{x}}}{P_{\boldsymbol{x}}^{(1)}+P_{\boldsymbol{x}}^{(2)}} \frac{\partial}{\partial\lambda}\big(P_{\boldsymbol{x}}^{(1)}+P_{\boldsymbol{x}}^{(2)}\big) \frac{\partial}{\partial p_{\boldsymbol{i}}}\big(P_{\boldsymbol{x}}^{(1)}+P_{\boldsymbol{x}}^{(2)}\big)\big(N^2-N+NP_{\boldsymbol{x}}^{-1}\big) \\
&+N(N-1)\sum_{\boldsymbol{x}\in\mathscr{O}_1} \frac{P_{\boldsymbol{x}}}{P_{\boldsymbol{x}}^{(1)}+P_{\boldsymbol{x}}^{(2)}} \frac{\partial}{\partial\lambda}\big(P_{\boldsymbol{x}}^{(1)}+P_{\boldsymbol{x}}^{(2)}\big) \sum_{\substack{\boldsymbol{y}\in\mathscr{O}_1\\ \boldsymbol{y}\neq\boldsymbol{x}}} \frac{P_{\boldsymbol{y}}}{P_{\boldsymbol{y}}^{(1)}+P_{\boldsymbol{y}}^{(2)}} \frac{\partial}{\partial p_{\boldsymbol{i}}}\big(P_{\boldsymbol{y}}^{(1)}+P_{\boldsymbol{y}}^{(2)}\big) \\
&+N(N-1)\sum_{\boldsymbol{x}\in\mathscr{O}_1} \frac{P_{\boldsymbol{x}}}{P_{\boldsymbol{x}}^{(1)}+P_{\boldsymbol{x}}^{(2)}} \frac{\partial}{\partial\lambda}\big(P_{\boldsymbol{x}}^{(1)}+P_{\boldsymbol{x}}^{(2)}\big) \sum_{\boldsymbol{y}\in\mathscr{O}_2} \frac{P_{\boldsymbol{y}}}{P_{\boldsymbol{y}}^{(2)}} \frac{\partial}{\partial p_{\boldsymbol{i}}} P_{\boldsymbol{y}}^{(2)} \\
&+N(N-1)\sum_{\boldsymbol{x}\in\mathscr{O}_1} \frac{P_{\boldsymbol{x}}}{P_{\boldsymbol{x}}^{(1)}+P_{\boldsymbol{x}}^{(2)}} \frac{\partial}{\partial p_{\boldsymbol{i}}}\big(P_{\boldsymbol{x}}^{(1)}+P_{\boldsymbol{x}}^{(2)}\big) \sum_{\boldsymbol{y}\in\mathscr{O}_2} \frac{P_{\boldsymbol{y}}}{P_{\boldsymbol{y}}^{(2)}} \frac{\partial}{\partial \lambda} P_{\boldsymbol{y}}^{(2)} \\
&+N(N-1)\sum_{\boldsymbol{x}\in\mathscr{O}_2} \frac{P_{\boldsymbol{x}}}{P_{\boldsymbol{x}}^{(2)}} \frac{\partial}{\partial\lambda} P_{\boldsymbol{x}}^{(2)} \sum_{\substack{\boldsymbol{y}\in\mathscr{O}_2\\ \boldsymbol{y}\neq\boldsymbol{x}}} \frac{P_{\boldsymbol{y}}}{P_{\boldsymbol{y}}^{(2)}} \frac{\partial}{\partial p_{\boldsymbol{i}}} P_{\boldsymbol{y}}^{(2)} \\
&+\sum_{\boldsymbol{x}\in\mathscr{O}_2} \left(\frac{P_{\boldsymbol{x}}}{P_{\boldsymbol{x}}^{(2)}}\right)^2 \frac{\partial}{\partial\lambda} P_{\boldsymbol{x}}^{(2)} \frac{\partial}{\partial p_{\boldsymbol{i}}} P_{\boldsymbol{x}}^{(2)} \big(N^2-N+NP_{\boldsymbol{x}}^{-1}\big) \\
= &\sum_{\boldsymbol{x}\in\mathscr{O}_1} \frac{\partial}{\partial\lambda} P_{\boldsymbol{x}} \frac{\partial}{\partial p_{\boldsymbol{i}}} P_{\boldsymbol{x}} \big(N^2-N+NP_{\boldsymbol{x}}^{-1}\big) \\
&+N(N-1)\sum_{\boldsymbol{x}\in\mathscr{O}_1} \frac{\partial}{\partial\lambda} P_{\boldsymbol{x}} \sum_{\substack{\boldsymbol{y}\in\mathscr{O}_1\\ \boldsymbol{y}\neq\boldsymbol{x}}} \frac{\partial}{\partial p_{\boldsymbol{i}}} P_{\boldsymbol{y}} + N(N-1)\sum_{\boldsymbol{x}\in\mathscr{O}_1} \frac{\partial}{\partial\lambda} P_{\boldsymbol{x}} \sum_{\boldsymbol{y}\in\mathscr{O}_2} \frac{\partial}{\partial p_{\boldsymbol{i}}} P_{\boldsymbol{y}} \\
&+N(N-1)\sum_{\boldsymbol{x}\in\mathscr{O}_1} \frac{\partial}{\partial p_{\boldsymbol{i}}} P_{\boldsymbol{x}} \sum_{\boldsymbol{y}\in\mathscr{O}_2} \frac{\partial}{\partial \lambda} P_{\boldsymbol{y}} + N(N-1)\sum_{\boldsymbol{x}\in\mathscr{O}_2} \frac{\partial}{\partial\lambda} P_{\boldsymbol{x}} \sum_{\substack{\boldsymbol{y}\in\mathscr{O}_2\\ \boldsymbol{y}\neq\boldsymbol{x}}} \frac{\partial}{\partial p_{\boldsymbol{i}}} P_{\boldsymbol{y}} \\
&+\sum_{\boldsymbol{x}\in\mathscr{O}_2} \frac{\partial}{\partial\lambda} P_{\boldsymbol{x}} \frac{\partial}{\partial p_{\boldsymbol{i}}} P_{\boldsymbol{x}}^{(2)} \big(N^2-N+NP_{\boldsymbol{x}}^{-1}\big).
\end{aligned}
$$

Hence,

$$
\begin{aligned}
\mathcal{S}_{\lambda,p_{\boldsymbol{i}}} = &N\sum_{\boldsymbol{x}\in\mathscr{O}_1} P_{\boldsymbol{x}}^{-1} \frac{\partial}{\partial\lambda} P_{\boldsymbol{x}} \frac{\partial}{\partial p_{\boldsymbol{i}}} P_{\boldsymbol{x}} \\
&+N(N-1)\sum_{\boldsymbol{x}\in\mathscr{O}_1} \frac{\partial}{\partial\lambda} P_{\boldsymbol{x}} \sum_{\boldsymbol{y}\in\mathscr{O}_1} \frac{\partial}{\partial p_{\boldsymbol{i}}} P_{\boldsymbol{y}} + N(N-1)\sum_{\boldsymbol{x}\in\mathscr{O}_1} \frac{\partial}{\partial\lambda} P_{\boldsymbol{x}} \sum_{\boldsymbol{y}\in\mathscr{O}_2} \frac{\partial}{\partial p_{\boldsymbol{i}}} P_{\boldsymbol{y}} \\
&+N(N-1)\sum_{\boldsymbol{x}\in\mathscr{O}_1} \frac{\partial}{\partial p_{\boldsymbol{i}}} P_{\boldsymbol{x}} \sum_{\boldsymbol{y}\in\mathscr{O}_2} \frac{\partial}{\partial \lambda} P_{\boldsymbol{y}} + N(N-1)\sum_{\boldsymbol{x}\in\mathscr{O}_2} \frac{\partial}{\partial\lambda} P_{\boldsymbol{x}} \sum_{\boldsymbol{y}\in\mathscr{O}_2} \frac{\partial}{\partial p_{\boldsymbol{i}}} P_{\boldsymbol{y}} \\
&+N\sum_{\boldsymbol{x}\in\mathscr{O}_2} P_{\boldsymbol{x}}^{-1} \frac{\partial}{\partial\lambda} P_{\boldsymbol{x}} \frac{\partial}{\partial p_{\boldsymbol{i}}} P_{\boldsymbol{x}}.
\end{aligned}
$$

Note that,

$$
\begin{aligned}
&\sum_{\boldsymbol{x}\in\mathscr{O}_1} \frac{\partial}{\partial\lambda} P_{\boldsymbol{x}} \sum_{\boldsymbol{y}\in\mathscr{O}_1} \frac{\partial}{\partial p_{\boldsymbol{i}}} P_{\boldsymbol{y}} + \sum_{\boldsymbol{x}\in\mathscr{O}_1} \frac{\partial}{\partial\lambda} P_{\boldsymbol{x}} \sum_{\boldsymbol{y}\in\mathscr{O}_2} \frac{\partial}{\partial p_{\boldsymbol{i}}} P_{\boldsymbol{y}} + \sum_{\boldsymbol{x}\in\mathscr{O}_1} \frac{\partial}{\partial p_{\boldsymbol{i}}} P_{\boldsymbol{x}} \sum_{\boldsymbol{y}\in\mathscr{O}_2} \frac{\partial}{\partial\lambda} P_{\boldsymbol{y}} \\
&+\sum_{\boldsymbol{x}\in\mathscr{O}_2} \frac{\partial}{\partial\lambda} P_{\boldsymbol{x}} \sum_{\boldsymbol{y}\in\mathscr{O}_2} \frac{\partial}{\partial p_{\boldsymbol{i}}} P_{\boldsymbol{y}} \\
= &\sum_{\boldsymbol{x}\in\mathscr{O}} \frac{\partial}{\partial\lambda} P_{\boldsymbol{x}} \sum_{\boldsymbol{y}\in\mathscr{O}} \frac{\partial}{\partial p_{\boldsymbol{i}}} P_{\boldsymbol{y}} = \frac{\partial}{\partial\lambda} \sum_{\boldsymbol{x}\in\mathscr{O}} P_{\boldsymbol{x}} \frac{\partial}{\partial p_{\boldsymbol{i}}} \sum_{\boldsymbol{y}\in\mathscr{O}} P_{\boldsymbol{y}} = 0.
\end{aligned}
$$

Therefore,

$$\mathcal{S}_{\lambda,p_{\boldsymbol{i}}} = N \sum_{\boldsymbol{x}\in\mathscr{O}_1} P_{\boldsymbol{x}}^{-1} \frac{\partial}{\partial\lambda} P_{\boldsymbol{x}} \frac{\partial}{\partial p_{\boldsymbol{i}}} P_{\boldsymbol{x}} + N \sum_{\boldsymbol{x}\in\mathscr{O}_2} P_{\boldsymbol{x}}^{-1} \frac{\partial}{\partial\lambda} P_{\boldsymbol{x}} \frac{\partial}{\partial p_{\boldsymbol{i}}} P_{\boldsymbol{x}},$$

yielding

$$\mathcal{S}_{\lambda,p_{\boldsymbol{i}}} = N \sum_{\boldsymbol{x}\in\mathscr{O}} P_{\boldsymbol{x}}^{-1} \frac{\partial}{\partial\lambda} P_{\boldsymbol{x}} \frac{\partial}{\partial p_{\boldsymbol{i}}} P_{\boldsymbol{x}} = \mathcal{I}_{\lambda,p_{\boldsymbol{i}}} \tag{A.48b}$$

by comparison with (A.39a).

For $\theta'_i = \theta'_j = p_{\boldsymbol{i}}$,

$$\mathcal{S}_{p_{\boldsymbol{i}},p_{\boldsymbol{i}}} = \mathbb{E}\left[\left(\frac{\partial l(\boldsymbol{\theta}')}{\partial p_{\boldsymbol{i}}}\right)^2\right],$$

the same approach as above and comparison with (A.40a) yields

$$\mathcal{S}_{p_{\boldsymbol{i}},p_{\boldsymbol{i}}} = N \sum_{\boldsymbol{x}\in\mathscr{O}} P_{\boldsymbol{x}}^{-1} \left(\frac{\partial}{\partial p_{\boldsymbol{i}}} P_{\boldsymbol{x}}\right)^2 = \mathcal{I}_{p_{\boldsymbol{i}},p_{\boldsymbol{i}}}. \tag{A.48c}$$

Finally, for $\theta'_i = p_{\boldsymbol{i}}$ and $\theta'_j = p_{\boldsymbol{j}}$,

$$\mathcal{S}_{p_{\boldsymbol{i}},p_{\boldsymbol{j}}} = \mathbb{E}\left[\frac{\partial l(\boldsymbol{\theta}')}{\partial p_{\boldsymbol{i}}} \frac{\partial l(\boldsymbol{\theta}')}{\partial p_{\boldsymbol{j}}}\right],$$

which becomes according to (A.41a)

$$\mathcal{S}_{p_{\boldsymbol{i}},p_{\boldsymbol{j}}} = N \sum_{\boldsymbol{x}\in\mathscr{O}} P_{\boldsymbol{x}}^{-1} \frac{\partial}{\partial p_{\boldsymbol{i}}} P_{\boldsymbol{x}} \frac{\partial}{\partial p_{\boldsymbol{j}}} P_{\boldsymbol{x}} = \mathcal{I}_{p_{\boldsymbol{i}},p_{\boldsymbol{j}}}. \tag{A.48d}$$

References

1. Tsoungui Obama HCJ, Schneider KA. A Maximum-Likelihood Method to Estimate Haplotype Frequencies and Prevalence alongside Multiplicity of Infection from SNP Data. Frontiers in Epidemiology. 2022;2.

2. Obama HCJT, Schneider KA. Estimating Multiplicity of Infection, Haplotype Frequencies, and Linkage Disequilibria from Multi-Allelic Markers for Molecular Disease Surveillance; 2023.

3. Schneider KA, Escalante AA. A Likelihood Approach to Estimate the Number of Co-Infections. PLOS ONE. 2014;9(7):e97899. doi:10.1371/journal.pone.0097899.

4. Schervish MJ. Sufficient Statistics. In: Schervish MJ, editor. Theory of Statistics. New York, NY: Springer; 1995. p. 111.
