# Supplementary material for: Molecular surveillance of multiplicity of infection, haplotype frequencies, and prevalence in infectious diseases
Source: PLoS Comput Biol. 2026 Jul 17;22(7):e1012955. doi: 10.1371/journal.pcbi.1012955 (PMC13485136; doi:10.1371/journal.pcbi.1012955)
Supplement: Supplementary file 2 [file pcbi.1012955.s002.pdf]

Documentation R code of manuscript "Molecular surveillance of multiplicity of infection, haplotype frequencies, and prevalence in infectious diseases" (V1.0.0)

Henri Christian Junior Tsoungui Obama, Kristan Alexander Schneider

Overview

The article "Molecular surveillance of multiplicity of infection, haplotype frequencies, and prevalence in infectious diseases" describes a maximum-likelihood method to estimate haplotype frequencies and multiplicity of infection (MOI) from unphased molecular data, as well as ways to perform further population-genetic analyses. This documentation describes how to apply the method in practice using the functions provided in the R script "MOI-MLE.R". The R script "MOI-tutorial.R" contains the code used throughout this documentation, and can be used as a tutorial to get acquainted with the method. The dataset "dataset.xlsx", available in Supporting information S1 Data, is used throughout this documentation. All materials are also available on GitHub at (https://github.com/Maths-against-Malaria/generalModel).

Loading the script in R

First, download the R script "MOI-MLE.R" from the GitHub repository https://github.com/Maths-against-Malaria/generalModel or the Supporting information S1 Data. Next, load the functions in the R script "MOI-MLE.R" using the command "source(<PATH>/MOI-MLE.R)", where <PATH> refers to the directory, where the R script is stored. For example, if you saved it in the directory "/home/johndoe/documents/src/", run the following code:

```
# Load R script
source("/home/johndoe/documents/src/MOI-MLE.R")
```

If the package "openxlsx" is not installed in your R environment, it will be installed in the background.

Once the script is loaded, several functions become available in your R environment, particularly, the main function "MLE", which calculates the maximum-likelihood estimates (MLEs) of MOI and haplotype frequencies from unphased molecular data, and the functions "PREV", "FI", and "pairwiseLD", which calculate the haplotype prevalence, the asymptotic variance of the estimator, and pairwise linkage disequilibrium (LD). Note that, the functions take molecular data in a specific format as input. The required data format is described next.

Data format

In the required format (referred to as a standard format), observations (samples) are represented by multiple rows. Namely, at each marker, the alleles present in an observation are entered in consecutive rows. This requires, that the first column in such a dataset specifies the sample IDs. As an illustration of a dataset with four markers, with alleles named (i) 130, 133 at marker m1; (ii) 201, 207, 210 at marker m2; (iii) 89, 91, 94, 99 at marker m3; and (iv) 140, 145, 148 at marker m4. Consider the following dataset with this genetic architecture (GA):

ID	m1	m2	m3	m4
S1	130	207	99	140
S1	133	201		
S1		210		
S2	133	201	99	140
S2	133	210	91	145
S3	130	207	91	148
S4	133			140
⋮	⋮	⋮	⋮	⋮
S99			89	145
S99			99	148
S100		201	94	148
S100		207		140

Here, observation S1, representing a super-infection, spans across three consecutive rows. Two alleles (130, 133) are present at marker m1, three alleles (201, 207, 210) at marker m2, one allele at marker m3 (99), and one allele (140) at marker m4.

Observation S3 spans over a single row, corresponding to a single infection with haplotype 130/207/91/148. In observation S100, any allelic information is missing at marker m1.

Hint: data in other formats

For datasets not in the standard format, the package **MLMOI** provides flexible functions to transform the data into the standard format while detecting input errors. The following code should be executed to install and load the package **MLMOI**:

```
# Install library "MLMOI"
install.packages("MLMOI")

# Load library
library("MLMOI")

# Consult documentation
?MLMOI
```

Note that the current version of the package "MLMOI" depends on the package "Rjava", and will not function without a correct installation of the latter.

Loading the dataset

To import the dataset "dataset.xlsx" (available in Supporting information S1 Data), which consists of information from four markers in standard format and is stored in "/home/johndoe/documents/exampleDataset/", first load the package "openxlsx" and load the dataset using the function "read.xlsx". To do so, run the following code:

```
# Load library openxlsx
library("openxlsx")

# Import the dataset in standard format
data <- read.xlsx('/home/johndoe/documents/exampleDataset/dataset.xlsx', 1)
```

The loaded dataset is assigned to the variable "data", which contains the following data frame:

```
## > data
##        ID   m1   m2   m3   m4
## 1     ID1  133  210   94  148
```

```
## 2      ID2  133  210   91  140
## 3      ID3  130  210   91  140
##        ...  ...  ...  ...  ...
## 147   ID99  133 <NA> <NA>  140
## 148  ID100  130  201   94  148
## 149  ID100 <NA>  210 <NA> <NA>
```

Estimating haplotype frequencies and MOI

Once the script "MOI-MLE.R" and the dataset <DATA> are loaded in the R environment, the function "MLE(<DATA>, <markers>, <...>)" derives the maximum likelihood estimates (MLEs) of the haplotype frequencies and MOI parameter. The input <DATA> is a dataset in the standard format, <markers> is an index vector specifying the columns of the markers in the data to be analyzed, and <...> represents optional arguments.

The function "MLE(<DATA>, <markers>, <...>)" outputs a list with four elements: (i) the estimated MOI parameter $\hat{\lambda}$; (ii) an array of the estimated non-zero haplotype frequencies $\hat{p}$ (the estimates are labeled by haplotypes composed by their allelic configuration by default – argument 'allelesName = TRUE' – or a simplified format, i.e., allele names at each marker represented by integers if the option 'allelesName = FALSE' is set; labels start with 'p' and '.' separates markers); (iii) an array of all detected haplotypes; and (iv) the effective sample size used for the estimation, i.e., the number of samples without missing data. The following code estimates MLEs from the above example dataset for markers 1 to 4:

```
## Choose markers of interest
markers <- 1:4

## Estimate MLEs
MLE(data, markers)
```

```
## $lambda
## [1] 0.968123
##
## $haplotype_frequencies
##                         [,1]
## p130.201.89.145  2.094589e-02
## p130.201.91.140  5.487696e-02
## p130.201.91.148  9.939004e-03
##      ...              ...
## p130.207.94.148  6.897002e-68
## p130.201.99.148  2.288823e-70
## p133.207.99.148  7.179864e-65
##
## $detected_haplotypes
##        m1    m2    m3   m4
##  [1,] "130" "201" "89" "145"
##  [2,] "130" "201" "91" "140"
##  [3,] "130" "201" "91" "148"
##  ...   ...   ...   ...   ...
## [55,] "130" "207" "94" "148"
## [56,] "130" "201" "99" "148"
## [57,] "133" "207" "99" "148"
##
## $used_sample_size
## [1] 82
```

Estimation of haplotype frequencies with known MOI

If a prior estimate of MOI is available, e.g., from empirical observations, it is possible to obtain MLEs for haplotype frequencies using the prior estimate as a plug-in. The function "MLE(<DATA>, <markers>, ...)" is capable of doing so by providing the plugin value of the MOI parameter. To do so, set the argument "plugin = $\hat{\lambda}_{\text{plugin}}$", where $\hat{\lambda}_{\text{plugin}}$ is the known value of the MOI parameter λ.

Assuming the plugin value $\hat{\lambda}_{\text{plugin}} = 1.0$, the MLEs for haplotype frequencies from the example dataset are obtained by running the code:

```
MLE(data, markers, plugin = 1.0, allelesName = FALSE)
```

```
## $lambda
## [1] 1
##
## $haplotype_frequencies
##                   [,1]
## p1.1.1.2  2.092049e-02
## p1.1.2.1  5.489177e-02
## p1.1.2.3  9.927741e-03
##    ...         ...
## p1.2.3.3  1.225492e-67
## p1.1.4.3  1.912450e-70
## p2.2.4.3  5.959151e-65
##
## $detected_haplotypes
##       [,1] [,2] [,3] [,4]
## [1,]     1    1    1    2
## [2,]     1    1    2    1
## [3,]     1    1    2    3
##  ...   ...  ...  ...  ...
## [55,]    1    2    3    3
## [56,]    1    1    4    3
## [57,]    2    2    4    3
##
## $used_sample_size
## [1] 82
```

Note that setting the argument "allelesName = FALSE" replaced allele 130 by the integer 1, and allele 133 by 2 at marker m1. The integers 1 and 2 represent the order of alleles 130, and 133 at marker m1, respectively.

Obtaining bias-corrected estimates

The bias of MLEs — although in general small – can be corrected. Bias-corrected estimates are obtained by setting the argument "isBC = TRUE". A non-parametric bootstrap bias correction is applied. Additionally, the number of bootstrap replicates can be set with the argument "replBC", with default value "replBC = 10 000".

To obtain bootstrap bias-corrected MLEs, using 15 000 replicates the following code should be executed:

```
MLE(data, markers, isBC = TRUE, replBC = 15000)
```

```
## $lambda
##    lambda
## 0.9594864
##
```

```
## $haplotype_frequencies
##                          [,1]
## p130.201.89.145  2.092126e-02
## p130.201.91.140  5.538426e-02
## p130.201.91.148  9.321593e-03
##      ...              ...
## p130.207.94.148  1.379400e-67
## p130.201.99.148  4.577646e-70
## p133.207.99.148  1.435973e-64
##
## $detected_haplotypes
##       m1    m2    m3   m4
##  [1,] "130" "201" "89" "145"
##  [2,] "130" "201" "91" "140"
##  [3,] "130" "201" "91" "148"
##  ...   ...   ...  ...  ...
## [55,] "130" "207" "94" "148"
## [56,] "130" "201" "99" "148"
## [57,] "133" "207" "99" "148"
##
## $used_sample_size
## [1] 82
```

Bootstrap confidence intervals

Equally-tailed $(1-\alpha) \times 100\%$ bootstrap confidence intervals (BCIs) can be obtained alongside the MLEs. This is achieved by setting the argument "isCI = TRUE" in the function MLE. By default 10 000 bootstrap replicates are used, which can be changed by setting a value to the argument "replCI'. Moreover, the significance level α (with default value 5%) can be specified by setting the argument "alpha", e.g., alpha = 0.10 for a 90% confidence level. With this option, MLEs are presented in an array with 3 columns in which the first column contains the MLEs while the second and third contain the lower and upper confidence points, respectively.

In particular, for the example dataset, using replCI = 15 000 bootstrap replicates and a confidence level of 90%, i.e., alpha = 0.10, the MLEs with BCIs are obtained by executing the following code:

```
MLE(data, markers, isCI = TRUE, replCI = 15000, alpha = 0.10)
```

```
## $lambda
##                  5%       95%
## 0.9681230 0.7862279 1.1747457
##
## $haplotype_frequencies
##                                 5%           95%
## p130.201.89.145  2.094589e-02  3.45638e-103 4.536652e-02
## p130.201.91.140  5.487696e-02  1.540735e-02 9.896500e-02
## p130.201.91.148  9.939004e-03  0.000000e+00 3.252737e-02
##      ...             ...            ...          ...
## p130.207.94.148  6.897002e-68  0.000000e+00 1.452442e-22
## p130.201.99.148  2.288823e-70  0.000000e+00 4.592070e-16
## p133.207.99.148  7.179864e-65  0.000000e+00 1.493176e-11
##
## $detected_haplotypes
##       m1    m2    m3   m4
##  [1,] "130" "201" "89" "145"
```

```
##  [2,] "130" "201" "91" "140"
##  [3,] "130" "201" "91" "148"
##  ...   ...   ...   ...  ...
## [55,] "130" "207" "94" "148"
## [56,] "130" "201" "99" "148"
## [57,] "133" "207" "99" "148"
##
## $used_sample_size
## [1] 82
```

Note that bias-corrected estimates with BCIs can be obtained by specifying the corresponding argument as in Section Obtaining bias-corrected estimates. For the example data, the bias-corrected estimates with 20 000 bootstrap replicates, i.e., replBC = 20 000 and 15 000 bootstrap replicates for confidence intervals with 90% confidence level is obtained by executing:

```
MLE(data, markers, isBC = TRUE, replBC = 20000, isCI = TRUE, replCI = 15000, alpha = 0.10)
```

```
## $lambda
##                   5%       95%
## 0.9580943 0.7850747 1.1687869
##
## $haplotype_frequencies
##                                5%            95%
## p130.201.89.145  2.112350e-02  1.709749e-117 4.567998e-02
## p130.201.91.140  5.534044e-02  1.606634e-02  9.778057e-02
## p130.201.91.148  9.214784e-03  0.000000e+00  3.254658e-02
##      ...              ...           ...           ...
## p130.207.94.148  1.379400e-67  0.000000e+00 1.005545e-22
## p130.201.99.148  4.577646e-70  0.000000e+00 6.421287e-16
## p133.207.99.148  1.435973e-64  0.000000e+00 1.359428e-11
##
## $detected_haplotypes
##       m1    m2    m3   m4
##  [1,] "130" "201" "89" "145"
##  [2,] "130" "201" "91" "140"
##  [3,] "130" "201" "91" "148"
##  ...   ...   ...   ...  ...
## [55,] "130" "207" "94" "148"
## [56,] "130" "201" "99" "148"
## [57,] "133" "207" "99" "148"
##
## $used_sample_size
## [1] 82
```

Note that obtaining bootstrap bias-corrected estimates alongside bootstrap confidence intervals can be computationally expensive, especially for a high number of bootstrap replicates, e.g., 20 000 bootstrap replicates for bias correction and 15 000 bootstrap replicates for confidence intervals involves 300 000 000 estimations.

Estimating haplotype prevalence

Estimates of haplotype prevalence are derived similarly to MLEs. More precisely, the function "PREV(<DATA>, <markers>,…)" derives estimates of haplotypes prevalence alongside MOI from the dataset <DATA>, considering the markers at columns <markers>. The output is similar to that of the function "MLE", with the second element of the output list containing an array of estimates of haplotype

prevalences. Moreover, bootstrap confidence intervals can also be obtained similarly as in the function "MLE" by setting the argument "isCI=TRUE".

The following code estimates haplotype prevalence from the first and second locus markers with 90% confidence intervals, using 15 000 bootstrap replicates:

```
## Choose markers of interest
markers <- 1:2

## Estimate haplotype prevalence
PREV(data, markers, isCI = TRUE, replCI = 15000, alpha = 0.10)
```

```
## $lambda
##             5%       95%
## 0.9677134 0.7486591 1.2183122

## $haplotypes_prevalence
##                      5%        95%
## q130.201 0.19483616 0.12359886 0.2698953
## q130.207 0.09150407 0.03839826 0.1483252
## q130.210 0.38820854 0.29851024 0.4788783
## q133.201 0.16246128 0.09648930 0.2318936
## q133.207 0.18284535 0.11476234 0.2556547
## q133.210 0.39224923 0.30338246 0.4830803

## $detected_haplotypes
##      m1    m2
## [1,] "130" "201"
## [2,] "130" "207"
## [3,] "130" "210"
## [4,] "133" "201"
## [5,] "133" "207"
## [6,] "133" "210"

## $used_sample_size
## [1] 85
```

Pairwise linkage disequilibrium estimates

The script "MOI-MLE.R" also provides a function to derive the pairwise linkage disequilibrium (LD) measures D' and r^2. The function is defined as "pairwiseLD(<DATA>, <markers>, <...>)", where the argument <DATA> is the dataset in the standard format, <markers> is a vector containing the column of the two markers of interest. The estimates of LD can be obtained with bootstrap confidence intervals as described in Section Bootstrap confidence intervals.

Given the example data, pairwise LD between the markers at columns 1 and 4, with a 90% confidence interval based on 20 000 bootstrap replicates, is obtained by executing the following code:

```
markersPair <- c(1,4)
pairwiseLD(data, markersPair, isCI = TRUE, replCI = 20000, alpha = 0.10)
```

```
##             5%  95%
## D'   0.06 0.03 0.24
## r^2  0.00 0.00 0.03
```

The code outputs a two-by-three array in which the first row contains the estimates of LD based on D' (first

column), alongside the lower and upper confidence points at columns two and three, respectively. The second row provides the estimates of pairwise LD based on r^2 with the corresponding lower and upper confidence points.

Asymptotic variance of MLEs

The asymptotic variance in terms of the observed and expected Fisher Information matrices can be calculated for (i) the MLEs, i.e., MOI parameter, and haplotype frequencies $(\hat{\lambda}, \hat{p}_1, \ldots, \hat{p}_H)$, (ii) mean MOI, and haplotype frequencies $(\hat{\psi}, \hat{p}_1, \ldots, \hat{p}_H)$, and (iii) mean MOI, and haplotype prevalence $(\hat{\psi}, \hat{q}_1, \ldots, \hat{q}_H)$.

Estimates are obtained using the function "FI(<DATA>, <markers>, <...>)". The dataset <DATA> is in the standard format, and the input <markers> is the vector of indexes of the markers of interest in the dataset. The optional inputs <...> are the boolean parameters "isPsi", used to estimate the variance for the mean MOI $\hat{\psi}$ instead of the MOI parameter $\hat{\lambda}$ (i.e., isPsi = FALSE by default), "isPrev", used to estimate the variance of the estimates of haplotype prevalence instead of haplotype frequencies (i.e., isPrev = FALSE by default), "isObserv" used to derive variance from the observed Fisher Information matrix instead of its expectation (i.e., isObserv = FALSE by default), and "allelesName", which is explained in Estimating haplotype frequencies and MOI.

The function outputs a list of two elements, the first is the covariance matrix, and the second a vector of variance for each parameter, i.e., the diagonal of the covariance matrix. By keeping the arguments "isPsi" and "isPrev" as default, the output is obtained for the parameters $(\hat{\lambda}, \hat{p}_1, \ldots, \hat{p}_H)$. However, setting "isPsi = TRUE" and "isPrev = FALSE", the output is obtained for $(\hat{\psi}, \hat{p}_1, \ldots, \hat{p}_H)$, and setting "isPsi = TRUE" and "isPrev = TRUE" yields estimates of asymptotic variance output for $(\hat{\psi}, \hat{q}_1, \ldots, \hat{q}_H)$.

Considering markers 1 and 2 in the example dataset, the expected Fisher Information matrix for the MLEs $(\hat{\lambda}, \hat{p}_1, \ldots, \hat{p}_H)$ is obtained by running the code:

```r
## Calculate asymptotic covariance matrix for MOI parameter and haplotype frequencies
markers <- c(1,2)
FI(data, markers)
```

```
$`Covariance matrix`
##            Lambda p130.201 p130.207 p130.210 p133.201 p133.207 p133.210
## Lambda    0.02553 -0.00018 -0.00012  0.00029 -0.00015 -0.00021  0.00038
## p130.201 -0.00018  0.00117 -0.00010 -0.00049 -0.00033 -0.00009 -0.00017
## p130.207 -0.00012 -0.00010  0.00062 -0.00025 -0.00001 -0.00021 -0.00004
## p130.210  0.00029 -0.00049 -0.00025  0.00210 -0.00011 -0.00019 -0.00105
## p133.201 -0.00015 -0.00033 -0.00001 -0.00011  0.00103 -0.00014 -0.00044
## p133.207 -0.00021 -0.00009 -0.00021 -0.00019 -0.00014  0.00105 -0.00042
## p133.210  0.00038 -0.00017 -0.00004 -0.00105 -0.00044 -0.00042  0.00212

## $Variance
##   Lambda p130.201 p130.207 p130.210 p133.201 p133.207 p133.210
##  0.02553  0.00117  0.00062  0.00210  0.00103  0.00105  0.00212
```

The following code needs to be executed to derive the expected asymptotic variance for the mean MOI and haplotype prevalences $(\hat{\psi}, \hat{q}_1, \ldots, \hat{q}_H)$ based on the first and second marker in the example dataset:

```r
## Calculate asymptotic covariance matrix for mean MOI and prevalence
markers <- c(1,2)
FI(data, markers, isPsi =  TRUE, isPrev = TRUE)
```

```
## $`Covariance matrix`
##          Mean_MOI q130.201 q130.207 q130.210 q133.201 q133.207 q133.210
## Mean_MOI  0.01100 -0.00080 -0.00039 -0.00125 -0.00067 -0.00077 -0.00122
## q130.201 -0.00080  0.00059 -0.00001 -0.00016 -0.00010  0.00002  0.00000
```

```
## q130.207 -0.00039 -0.00001  0.00026 -0.00008  0.00002 -0.00006  0.00002
## q130.210 -0.00125 -0.00016 -0.00008  0.00142  0.00002 -0.00001 -0.00051
## q133.201 -0.00067 -0.00010  0.00002  0.00002  0.00049 -0.00002 -0.00015
## q133.207 -0.00077  0.00002 -0.00006 -0.00001 -0.00002  0.00052 -0.00013
## q133.210 -0.00122  0.00000  0.00002 -0.00051 -0.00015 -0.00013  0.00143

## $Variance
## Mean_MOI q130.201 q130.207 q130.210 q133.201 q133.207 q133.210
## 0.01100  0.00059  0.00026  0.00142  0.00049  0.00052  0.00143
```

To obtain the observed Fisher information rather than the expected information, one should set the argument "isObserv = TRUE". The following code provides such estimates:

```r
markers <- c(1,2)
FI(data, markers, isPsi = TRUE, isPrev = TRUE, isObserv = TRUE)
```

```
## $`Covariance matrix`
##          Mean_MOI q130.201 q130.207 q130.210 q133.201 q133.207 q133.210
## Mean_MOI  0.01043 -0.00075 -0.00037 -0.00120 -0.00063 -0.00071 -0.00118
## q130.201 -0.00075  0.00051 -0.00001 -0.00010 -0.00003  0.00000 -0.00007
## q130.207 -0.00037 -0.00001  0.00023 -0.00006  0.00001 -0.00003  0.00000
## q130.210 -0.00120 -0.00010 -0.00006  0.00128 -0.00004 -0.00004 -0.00039
## q133.201 -0.00063 -0.00003  0.00001 -0.00004  0.00041 -0.00001 -0.00009
## q133.207 -0.00071  0.00000 -0.00003 -0.00004 -0.00001  0.00048 -0.00011
## q133.210 -0.00118 -0.00007  0.00000 -0.00039 -0.00009 -0.00011  0.00130

## $Variance
## Mean_MOI q130.201 q130.207 q130.210 q133.201 q133.207 q133.210
## 0.01043  0.00051  0.00023  0.00128  0.00041  0.00048  0.00130
```

Generating datasets

The function "datasetGen(<P>, <lambda>, <N>, <GA>)" generates simulated datasets (e.g., for simulation purposes). The data generated is in a specific format, referred here to as "input format". Before the usage of the function is presented, the specifics of the input format are described.

Input data format

Assume the data is generated for a genetic architecture of L markers containing allelic information, e.g., single nucleotide polymorphisms (SNPs), microsatellite markers (STRs), or a combination of both marker types. Furthermore, assume n_k alleles segregating at marker k ($k \in 1, \ldots, L$). Therefore, each record in the dataset (sample), is a vector of length L, where the k-th entry codes which alleles are present at marker k. Importantly, each sample has a sample ID.

For each marker, binary coding indicates which alleles are present. Namely, at marker k the absence and presence of the n_k possible alleles can be represented by a 0-1 vector of length n_k, with '0' and '1' corresponding to absence and presence, respectively. For example, the vector $(1, 0, \ldots, 0)$ indicates the presence of only the first allele, the vector $(1, 1, \ldots, 1)$ the presence of all alleles, and the vector $\mathbf{0} = (0, \ldots, 0)$, i.e., the absence of all alleles, indicates missing data. Each such vector uniquely corresponds to the binary representation of an integer ranging from 0 to $2^{n_k} - 1$, with 0 indicating missing data at locus k, 1 indicating the presence of only allele 1, and $2^{n_k} - 1$ indicating the presence of all alleles. This integer encodes the absence and presence of alleles at each marker. This data format is referred to as "input format".

As an example, in this format, a dataset of sample size $N = 100$ with two markers with $n_1 = 4$ and $n_2 = 3$ alleles is an array with integer entries ranging from 0 to $2^{n_1} - 1 = 15$ and 0 to $2^{n_2} - 1 = 7$ at the first and second marker, respectively. A specific example is:

ID	Marker1	Marker2
samp1	3	7
samp2	2	5
samp3	11	2
⋮	⋮	⋮
samp99	0	0
samp100	0	3

For sample 'samp1', the entries 3 and 7, correspond to the 0-1 vectors $(1, 1, 0, 0)$ and $(1, 1, 1)$, indicating that the first two alleles are present at Marker1 and all three alleles are present at Marker2. In sample "samp99" the entries 0, corresponding to $(0, 0, 0, 0)$ and $(0, 0, 0)$ indicate missing data at both markers. Sample samp100 has missing data only at Marker1, but the first two alleles are present at Marker2, as entry 3 corresponds to the vector $(1, 1, 0)$.

Generating data in the input format

To generate datasets in the input format with the function "datasetGen(<P>, <lambda>, <N> <GA>)", one needs to specify the following arguments: (i) a vector representing the haplotype distribution '<P>', e.g., <P> = $c(0.45, 0.25, 0.2, 0.1)$; (ii) the MOI parameter '<lambda>', e.g., <lambda> = 0.5; (iii) the sample size '<N>', e.g., <N> = 100; and (iv) a vector containing the genetic architecture '<GA>', e.g., <GA> = $c(2, 2)$ (two markers with 2 alleles each). The length of vector <P> should equate the product $n_1 n_2 \dots n_l$, i.e., for <GA> = $c(2, 2)$, <P> should be of length 2×2=4. Haplotypes can have a frequency 0.

Given the above arguments, the function "datasetGen(<P>, <lambda>, <N> <GA>)" uses the framework presented in the manuscript to sample <N> MOI values based on a conditional Poisson distribution. Multinomial sampling of haplotypes for each of the <N> MOI values yields the <N> records of the dataset in the correct input format. These operations are performed internally by subroutine functions defined in the script "MOI-MLE.R".

The following code generates a dataset for a haplotype frequency distribution <P> = $c(0.45, 0.25, 0.2, 0.1)$, MOI parameter <lambda> = 0.5, sample size <N> = 100, and the genetic architecture <GA> = $c(2, 2)$:

```r
## Generate simulated data
data <- datasetGen(c(0.45,0.25,0.2,0.1), 0.5, 100, c(2,2))
```

The output is an array stored as the variable "data". It has the following form:

```
## > data
##        [,1] [,2]
##   [1,]    1    2
##   [2,]    1    1
##   [3,]    2    1
...  ...     ...  ...
##  [98,]    1    1
##  [99,]    1    3
## [100,]    1    3
```

Deriving MLEs from simulated datasets

The estimation of haplotype frequencies and MOI from simulated data is performed in two steps. First, the function "datasetXNx(data)" converts the simulated data into a list of length two containing an array (matrix) of observations present in the dataset, and a vector of the number of times each observation occurs in the dataset, i.e., using the notation in the article "Molecular disease surveillance using a computational method to estimate haplotype frequencies, prevalence and multiplicity of infection", each row in the array

corresponds to an observation $\boldsymbol{x}$ and the corresponding entry in the vector to $n_{\boldsymbol{x}}$. To create a converted version, say "dataC", of the dataset "data" generated above run the code:

```
## Convert simulated data
dataC <- datasetXNx(data)
```

The converted data "dataC" has the following form:

```
## > dataC
## $Observations
##       [,1] [,2]
##  [1,]    1    1
##  [2,]    1    2
##  [3,]    1    3
##  [4,]    2    1
##  [5,]    2    2
##  [6,]    2    3
##  [7,]    3    1
##  [8,]    3    2
##  [9,]    3    3

## $Observations_count
## 1-1 1-2 1-3 2-1 2-2 2-3 3-1 3-2 3-3
##  34  30   8  12   6   2   2   2   4
```

Finally, the MLEs are obtained by passing the converted dataset as an argument to the function "baseModel(<DATA>, <GA>)", alongside the genetic architecture used to simulate the dataset. For the dataset above, the following code derives the MLEs:

```
## Deriving the MLEs
baseModel(dataC, c(2,2))
```

```
## $lambda
## [1] 0.5397058

## $haplotype_frequencies
##         [,1]
## 1 0.39537523
## 3 0.36298019
## 2 0.14870510
## 4 0.09293948
```

The output is a list of two elements. The first is the estimate of the MOI parameter, and the second is an array containing the estimated haplotype frequencies. Haplotypes are named by integers ranging from 1 to the product $n_1 n_2 \dots n_l$ – here 2×2=4. See article for a detailed description of this representation.

Importantly, if one is not interested in the converted dataset, the MLEs can be obtained in one step by running the code:

```
## Deriving the MLEs
baseModel(datasetXNx(data), c(2,2))
```

```
## $lambda
## [1] 0.5397058

## $haplotype_frequencies
##         [,1]
## 1 0.39537523
## 3 0.36298019
```

```
## 2 0.14870510
## 4 0.09293948
```
