# Supplementary material for: Molecular surveillance of multiplicity of infection, haplotype frequencies, and prevalence in infectious diseases
Source: PLoS Comput Biol. 2026 Jul 17;22(7):e1012955. doi: 10.1371/journal.pcbi.1012955 (PMC13485136; doi:10.1371/journal.pcbi.1012955)
Supplement: Supplementary file 3 [file pcbi.1012955.s005.pdf]

Response to reviewers

Our responses are highlighted in blue and indented.

Reviewer #1:

Obama et al., present a novel statistical method for inferring the multiplicity of infection MOI and haplotype frequencies of pathogens within individuals from genetic data. The method addresses an important problem, as MOI can significantly complicate efforts to model the transmission, persistence and prevalence of infectious diseases.

Due to time constraints, I have not been able to go through all the derivations provided in full-detail. However, I have checked that the associated code implementing the methods runs as described. While I believe that this method provides a valuable addition to the literature, its limitations could be better described and I have outlined a number of issues that need to be addressed prior to publication.

> **Response**: We are grateful for the reviewer's constructive suggestions, particularly on benchmarking the performance of our method. We also want to point out that this work was part of the PhD thesis of the first author HCJTO, and was recently defended successfully. The mathematical details have been checked by 3 reviewers of the thesis, hence we can reassure that these are correct. While we have a large amount of agreement with the reviewer, we intentionally tried to keep certain arguments and corrections brief to facilitate the readability of the manuscript. In our response, we want to give more details about the background of our presentation.

Major issues:

1. The authors focus primarily on data that considers only a very small number of loci (<100 markers). While this is certainly useful, many modern genomic studies of major human pathogens including Malaria typically consider many thousands of loci. For example, the MalariaGen project has whole genome sequencing from over 20,000 Plasmodium falciparum samples. It is important that the authors more clearly demonstrate the computational limitations of their approach. This could easily be done by generating a plot of the compute time versus dataset complexity in both the number of loci and number of samples. The authors should also describe some of the literature that attempts to make use of the large number of loci to more

accurately estimate MOI and phase genotypes using WGS data. For example, methods such as DEploid (Zhu et al., 1017) and coiaf (Paschalidis et al., 2023) can consider many thousands of SNPs in a reasonable amount of time.

Response: We realized that we kept our discussion on alternative approaches rather brief in the introduction, mainly because this manuscript is about a multi-allelic generalization of our previously published biallelic method. Our method is an "exact full-haplotype" method intended for a relatively small set of genetic markers, as it occurs in many malaria applications. In the revised version, in the introduction, we are more explicit about this fact/limitation and use the two suggested references of DEploid and coiaf to contrast the different targeted data applications. Particularly, we discuss why sequencing errors are more important for data sets with a large number of SNPs than for a standardized panel of resistance-conferring mutations (which is a typical application for our method). Additionally, in the discussion, we mention how we can use techniques of DEploid and coiaf methods to improve the computational time of the model described in the manuscript.

We found it a fantastic suggestion to benchmark the computational time of the model. We now analyze the whole genome Pf8 dataset of the MalariaGen project in two ways. First, we use the ready-to-use dataset on drug resistance markers. The data is subdivided for each year, by country, and study. The model is benchmarked by analyzing each of the resulting datasets and by recording the time taken to derive the maximum-likelihood estimates in each case. The computational time is then plotted for each dataset as a function of the maximum number of polymorphic markers in samples. The plot, as well as its description, is added to the manuscript in a new subsection of the results section. Here, the devil is in the details. The Pf8 data set is not perfect regarding the quality of the data. We used an approach to include even low-quality data (which is suggested to be disregarded by the MalariaGen project) to allow for larger and more complex data sets. Some studies presumably also contain samples from different endemic regions within a country. We did not stratify those datasets, as we are interested in computational speed rather than meaningful analyses. Second, we extracted SNP data from chromosome 4 of the Pf8 dataset around *Pfdhfr* from a study conducted in Cameroon in 2013. The choice was rather arbitrary, and mainly used because our data application examples are also from Cameroon. We used this data to create several data sets starting

from all SNPs flanking *Pfdhfr* in a certain window. We increased the window size from 100bp up- and downstream in steps of 100bp to 1.1kb and ascertained the computational speed for all datasets.

2. The manuscript is currently quite hard to follow as many of the parameters are defined in the methods but not in the main text where they are first introduced (i.e lines 197-200). While I appreciate that including all derivations in the results would be tedious, the parameters should all be defined where they are first stated.

Response: We fully agree with the reviewer. When preparing the manuscript, we did not think of the fact that the methods section is at the end of the manuscript in PLoS Comp. Biol., and we flipped the sections at a rather late stage, with a certain blind spot on readability. We now made sure to specify what the model parameters are at each of their first occurrences, and explained the notation wherever it seemed appropriate.

3. The implementation of the model currently estimates unrealistically low haplotype frequencies. For example, line 29 of the 'MOI-tutorial.R' code outputs many (~50%) haplotypes with frequencies well below the limit of detection (0.01) given the example only contains 100 samples. The tendency of the approach to assign unrealistic frequencies to certain haplotypes needs to be discussed carefully in the text and perhaps a warning could be associated with results such as these that are unlikely to be accurate. Ideally, the method would include an approach to shrink these frequencies to zero perhaps using a regularisation penalty.

Response: The reviewer is correct! In fact, currently we are working on an approximate implementation that limits the number of possible haplotypes. This method is intended to be exact, i.e., to include all possible haplotypes which are compatible with the observations. For the applications we have in mind, e.g., drug-resistance, or a moderate quantity of genetic markers, we believe it is important to do so. This becomes pointless once the number of genetic markers increases. E.g., when considering 30 STR markers with 10 alleles each (which our method could not handle computationally anyway), there will be 10^30 possible haplotypes, a number which exceeds the number of parasite haplotypes which have ever existed. However, when considering only 5–6 markers, the situation is different. In general, our method gives very low frequency estimates to haplotypes, whose existence is not guaranteed by the data. In other words, if a haplotype occurs in a single infection in 1 out of 500 samples, its frequency will be on the order of 0.2% and its existence is guaranteed. However, if a haplotype is not directly

observed (presumably because it does not exist), the frequency estimate is typically very low (e.g., 10^-256). In practice, these frequencies would be rounded to 0, without jeopardizing the accuracy of the frequencies of “meaningful” haplotypes. The more relevant practical problem is that many haplotypes can exist with a frequency of around 0.2% and these cannot be reported in tables. This problem is also not mitigated if we restrict the number of haplotypes. In applications such as drug resistance, one also does not want to neglect certain haplotypes at low frequency. In fact, one wants to detect haplotypes with specific resistance-conferring mutations while they are rare. This is the reason we refrain from addressing the suggestion of shrinkage methods (which we think is a good idea) in the revision. In practice, it is often clear which haplotypes are of interest and which can safely be ignored. The long story short, we agree with the reviewer, added some discussion, but want to point out that — for the sake of brevity — we could not add all the details that would be worth discussion.

4. Identifying SNPs and microsatellites is an error prone process. It is important that the authors consider what impact errors in the input data have on the results of the method. A plot indicating the impact of different levels of error in the input data would be very useful to wider readers.

Response: We agree partly with this comment. The severity of the problem is different depending on the applications. When considering standardized microsatellite panels or SNP panels, such as SNPs from barcodes or resistant markers, the existence of certain alleles is secured. When it comes to microsatellites, one is actually not interested in the haplotypes per se but rather measures of, e.g., genetic diversity. These will not be too much impacted by errors in the data. Drug resistance markers are well established, and sequencing errors are less of a problem and easier to spot, particularly since only non-synonymous amino-acid substitutions are of interest. It is different when one attempts to look at a larger number of SNPs, without having a chance to manually verify each SNP. This becomes evident from working with the Pf8 data set, for instance. Since the data does not contain raw reads, it is infeasible to check how the data was mapped to the reference genome and how trustworthy the sequence alignment is. Since this is often performed by companies like Novogen, researchers often do not know the details of how their own samples were processed. One strategy to address data quality is to preprocess the data, as is done in coiaf. Another approach is to preprocess the data with an error model, another one, like in DEploid is to assume an error model in the likelihood function, and a third

approach would be to assume an error model to manipulate the data set and rerun the estimates in a bootstrap fashion to ascertain uncertainty. Particularly, with multi-allelic markers like microsatellites, the best mutational model is difficult to determine. We believe adding any mutational model in the current manuscript would exceed its scope. Hence, we decided to address the problem in the discussion and make suggestions for future extensions.

Minor issues:

5. The authors refer to the method being applicable to ‘arbitrary genetic architecture’ in a number of places. It is not clear what ‘arbitrary’ means in this context. For example, the method is not applicable to loci that are not consistently present in all genomes of a species. I think this statement should be replaced by a more specific one.

Response: We agree with this statement, and we have clarified this in the text. The genetic architecture in our context refers to the number of markers and the number of alleles per marker. Therefore, the term ‘arbitrary’ refers to a random number of markers and alleles per marker. We pointed that out more prominently. We also want to mention that the formulae apply to an arbitrary genetic architecture. However, the implementation of the resulting algorithm is limited by computational constraints.

6. The summary of alternative methods (lines 94-107) should be made slightly more detailed. Currently, it requires the reader to look up many of the references provided to understand what the authors are referring to (i.e I don’t automatically have a good intuition for what ‘ad-hoc methods’ are). It would be great if the ‘ad-hoc’ methods and the first statistical model (ref. 2) are explained with an additional sentence or two.

Response: This is a good suggestion. We added the additional sentences in the introduction; see also our responses above.

7. Line 74: “Co-transmitted” would normally mean the transmission of multiple genotypes within a single transmission event. This does not match up with the definition of MOI which is correctly and nicely stated later on.

Response: We understand the confusion this term might raise, and we have improved the wording to avoid this problem. However, we intentionally have a “fuzzy” definition in line 74, because the next paragraph starts with the fact that MOI is ambiguously defined in the literature, which motivates our more clear-cut definition.

8. Some of the figures have quite low resolution which should be addressed.

> **Response:** We know the problem, and frequently make the same comment when we are in the reviewer role. We uploaded the high-resolution formats (unfortunately, PLoS stopped accepting vectorized formats). Sometimes, in the PDF that is created by the editorial manager, figures look like they have low resolution, but the high-resolution versions are in the editorial management system.

Reviewer #2:

Tsoungui Obama and Schneider propose a method to estimate haplotype frequencies, haplotype prevalences and the multiplicity of infection based on unphased data from multiple multiallelic markers. The key advance relative to the authors' previous work (references [10] and [12]) appears to be a generalisation to the case of multiple multiallelic markers. An expectation-maximisation algorithm is derived to compute maximum likelihood estimates for haplotype frequencies and the multiplicity of infection, which are then plugged into derived formulae for haplotype prevalences. Asymptotic variances and finite sample properties of these estimators are considered. The practical utility of this method in the context of malaria genomic epidemiology is illustrated through an analysis of markers associated with drug resistance in Plasmodium falciparum.

Major comments

1. It would be useful if the authors could articulate more clearly the advance represented by the current manuscript, relative to the references [9], [10], [12], [22] and [25], particularly in the introduction and discussion. For instance, in the section "Deriving the EM-algorithm" of the Mathematical Appendix, the authors state that their "derivation generalizes the ones in [1] for n biallelic loci and [2] for two multiallelic loci to the case of n multiallelic loci. Namely, it turns out that the structure of the derivations is almost identical and only needs slight modifications"; this parallel, however, does not seem to be mentioned in the main text.

> **Response:** The structure of [10] and [12] is essentially the same as in this work. There are only slight differences in the notation. In fact, after completing [10] and [12], it became clear to us that a general genetic architecture could be used as presented here. We point this out explicitly in the revised version. The likelihood function has the same form, as it should have, and the structure of the EM-algorithm is the same.

However, we needed to explain the notation, particularly for the results on the Fisher information, which were not present in [10] and [12]. Due to changes in the notation, we did not want to just refer to [10] and [12] and state that the proofs are applicable with “obvious modifications”, but rather present the proof in a concise, standalone notation. [9, 22, 25] are applied to single markers. We wanted to avoid lengthy comparisons in the introduction and discussion. We added an explanation in the discussion why it is insufficient to use [9,10, 12] for the data analysis used as an example. Furthermore, we added that in the case of a single marker [9, 22, 25], the model has an exponential family for which is lost for two or more markers.

2. To improve the readability of the main text, perhaps the authors could consider introducing a table of notation? The equations stated in the main text results, with forward-reference to equations in the methods, are difficult to interpret. For instance, the PGF G first appears in Equation (1b) but is defined later on in the text.

Response: We agree. When we prepared the manuscript, we initially described the methods before the results section. In the PLoS Computational Biology format, this is reversed, and we did not notice the problem. We added a notation Table and made sure the notation is properly described in the text when it appears.

3. The authors frame the statistical model with respect to the notion of superinfection vs co-transmission. However, my understanding is that the statistical model is more simply predicated on the assumption of a Poisson-distributed number of unrelated/independent clones within a sample. I wonder if it might be clearer to introduce the model with respect to the latter notion, and then link this back to a more ‘mechanistic’ notion of superinfection? To aid readability, perhaps the authors could include a self-contained summary or list of model assumptions?

Response: The reviewer is correct. Essentially, a more mathematical presentation could state that the Poisson parameter is estimated and the common frequency distribution of the multinomial distributions. Also, in practice, the super-infections approximate co-infections if there is not too much relatedness between the transmitted haplotypes. The assumption of independent clones essentially reflects the idea of super-infections, as clones will not be independent if co-infections dominate. Anyhow, for three reasons, we believe the current presentation is more appropriate. First, the biological interpretation between super- and co-infections is important, and

more methods being developed for fine-grained data (100s of SNPs) to assess relatedness of haplotypes within an infection. Therefore, we believe the current presentation is better suited. Second, researchers who can verify the methodological details are not our primary target audience. The method is developed for biologists. We know that a certain methodological rigor is necessary for our purpose, but we tried to make the method understandable by the many illustrations we provided for a less mathematical audience. For empirical researchers, motivating the biology is more important than the mechanistic assumptions. Third, to be consistent with our previous research, we want to follow the current format. We added a comment that the model is equivalent to multinomial sampling with a Poisson-distributed number of trials on a fixed frequency distribution. Although the idea of adding a figure that describes the model is appealing, and we actually came up with a good idea, we feel that we already have many figures. Particularly since we added two new ones in the revised version.

Minor comments

4. Perhaps the authors could provide several examples of pathogens or organisms, beyond falciparum malaria, to which this method could be applied? Perhaps the authors could also comment on the interpretation of superinfection if this method was applied to vivax malaria, especially given the inoculation of multiple hypnozoites per mosquito bite and related relapses?

Response: We added a discussion regarding P. vivax. Namely, for the estimates of the frequency distribution, relapses are not relevant according to the model of Schneider Salas (2022). We added this comment in the discussion when reflecting on super- vs. co-infections. We also added some other examples of diseases to which the model could be applicable.

5. Some of the terminology used by the authors is ambiguous. For instance, the term “general genetic architecture” is defined to refer to “multiple multiallelic markers” on lines 590 and 647, but is used earlier in the introduction without definition. Rather than referring to “more complicated underlying genetic architectures” or “simple genetic architectures” (line 118), it would be useful to explicitly specify the case in consideration.

Response: We agree and specify the “general genetic architecture” now when it first occurs.

6. In Table 2, why are there MOI estimates < 1if the method is tailored to disease-positive specimens?

> **Response:** This is because we report the MOI parameter (lambda) of the conditional Poisson distribution in Table 2 rather than the mean MOI. We changed this in the revised version to avoid confusion.

7. Line 783: should “beta distribution” be replaced with “gamma distribution”?

> **Response:** Of course, the negative binomial is a gamma-Poisson mixture.

8. In the Introduction (line 102), the authors mention that references [13] and [14] also handle multiple multiallelic markers; given the generalisation of the authors’ previous work to the case of multiple multiallelic markers appears to be a key advance, a brief explanation of the key difference between references [13] and [14], and the present model, would be useful.

> **Response:** Now we refer to the data application and the discussion, where we explain why the generalization is important for certain analyses.
