# Supplementary material for: Molecular surveillance of multiplicity of infection, haplotype frequencies, and prevalence in infectious diseases
Source: PLoS Comput Biol. 2026 Jul 17;22(7):e1012955. doi: 10.1371/journal.pcbi.1012955 (PMC13485136; doi:10.1371/journal.pcbi.1012955)
Supplement: Supplementary file 4 [file pcbi.1012955.s006.pdf]

Response to reviewers: Round 2

We appreciate the positive feedback. Our responses are marked in blue.

Reviewer #1:

1. The authors have addressed most of my concerns, and I appreciate the addition of the new benchmarking section. While CPU time is now reported, several references are made to the algorithm running out of memory. The memory usage of the algorithm should also be stated, or at the very least the maximum amount of memory available during benchmarking should be clearly specified so that readers can properly interpret what "memory overflow" refers to

   Response: We appreciate the positive feedback!
   We clarified the specifications of the computer (in Methods) on which the benchmarking simulations were run. We also specified what type of errors occurred (session crashed or memory exhausted) in the text where appropriate. We want to point out that memory allocation in R is operating system-specific. While R directly allocates memory, on Windows, it is handled by the operating system; on Linux/macOS (we used the latter). These facts are now clearly described in the manuscript.

Reviewer #2:

Major comments:

1. The authors have largely addressed my previous comments. The readability of the revised manuscript is much improved with respect to notation, and the method is better contextualized in the literature.

   Response: We appreciate the positive feedback!

2. I appreciate the authors' point that the manuscript is largely targeted to a biological audience. However, I would still argue that a concise, self-contained list of key model assumptions and rationales/limitations could be practically useful for practitioners to decide whether the present model is suited to their particular context (e.g. the number of markers, the expected degree of genotyping error and the associated error structure, genotype missingness, whether markers are relatively fast or slow-evolving, etc), and to be aware of potential biases or artefacts when interpreting the model outputs (e.g. due to outliers, imputation or limited sample sizes). Given the model assumptions are

largely motivated in the context of falciparum malaria, this could be particularly useful for broader applications to other diseases.

Response: We found this a good suggestion. We added a section "Key assumptions and applicability" to the results after the estimator was introduced. We found this to be a prominent position for readers to notice these important aspects. This section involves some discussion, as the reason for the model assumptions has to be explained carefully. We believe we touched on important aspects, without going into too much detail. Especially regarding the error structure of the model, we could not go into the level of detail we wished to. Ideally, one would get different types of molecular data and with different assays from the same samples and compare the results. E.g., for SNP barcodes TaqMan assays or melting assays can have different errors, and those will be different from obtaining the same SNPs from whole sequencing data. There are many aspects we could not discuss in detail, such as error models under the assumption of linkage equilibrium (e.g., THE REAL McCOIL, or the method of Plucinskietal 2015 AAC to distinguish recrudescence from reinfection), which implicitly assumes independent errors between markers. We refrained from discussing details between different methods. This would be more appropriate in the context of an overview article comparing methods and their advantages/limitations. Anyhow, we think we reach middle ground.

3. Perhaps the authors could also comment on any biases that might arise if this method (predicated on the assumption of independent pathogen variants) is applied to data with significant relatedness between pathogen variants (e.g. due to significant co-transmission as characterised by Wong et al [doi: 10.1186/s13073-017-0398-0] or related relapses of vivax malaria)? Perhaps the authors could also expand on lines 595-596 to discuss possible biases in MOI estimates due to selection?

    Response: This is an extremely important point, especially since it becomes more and more feasible to generate molecular data that permits studying relatedness. We address this point briefly in the new section (see above) and comment on this in more detail in the discussion. Specifically, we expanded the paragraph that mentioned relatedness. We also expanded lines 595-596 and explained in more detail why MOI estimates are more reliable from neutral markers.

Minor comments:

1. It might be worth including SH in Table 1.

    Response: We added the definition of Sh in Table 1 as recommended.

Other changes: During the revision, we noticed an error in the derivation of the Fisher information of transformed parameters (see page 16). Namely, we confused a matrix and its inverse, which resulted in a more lengthy derivation. This did not affect the results of the manuscript. We double-checked the correctness of the implementation of the methods.
