# Supplementary material for: Molecular surveillance of multiplicity of infection, haplotype frequencies, and prevalence in infectious diseases
Source: PLoS Comput Biol. 2026 Jul 17;22(7):e1012955. doi: 10.1371/journal.pcbi.1012955 (PMC13485136; doi:10.1371/journal.pcbi.1012955)
Supplement: Peer review history

# Peer review: Decision Letter 0

**DOI:** 10.1371/journal.pcbi.1012955.r001

**Authors:** James McCaw, Jennifer Flegg

30 Jul 2025

PCOMPBIOL-D-25-00481

Molecular surveillance of multiplicity of infection, haplotype frequencies, and prevalence in infectious diseases

PLOS Computational Biology

Dear Dr. Tsoungui Obama,

Thank you for submitting your manuscript to PLOS Computational Biology. I apologise for the extended time to get back to you, but we have now secured two reviews of your manuscript.

After careful consideration, we feel that it has merit but does not fully meet PLOS Computational Biology's publication criteria as it currently stands. Therefore, we invite you to submit a revised version of the manuscript that addresses the points raised during the review process.

Please submit your revised manuscript within 60 days Sep 29 2025 11:59PM. If you will need more time than this to complete your revisions, please reply to this message or contact the journal office at ploscompbiol@plos.org. When you're ready to submit your revision, log on to https://www.editorialmanager.com/pcompbiol/ and select the 'Submissions Needing Revision' folder to locate your manuscript file.

Please include the following items when submitting your revised manuscript:

* A rebuttal letter that responds to each point raised by the editor and reviewer(s). You should upload this letter as a separate file labeled 'Response to Reviewers'. This file does not need to include responses to formatting updates and technical items listed in the 'Journal Requirements' section below.

* A marked-up copy of your manuscript that highlights changes made to the original version. You should upload this as a separate file labeled 'Revised Manuscript with Track Changes'.

* An unmarked version of your revised paper without tracked changes. You should upload this as a separate file labeled 'Manuscript'.

If you would like to make changes to your financial disclosure, competing interests statement, or data availability statement, please make these updates within the submission form at the time of resubmission. Guidelines for resubmitting your figure files are available below the reviewer comments at the end of this letter

We look forward to receiving your revised manuscript.

Kind regards,

James M McCaw, PhD

Academic Editor

PLOS Computational Biology

Jennifer Flegg

Section Editor

PLOS Computational Biology

**Journal Requirements:**

1) We ask that a manuscript source file is provided at Revision. Please upload your manuscript file as a .doc, .docx, .rtf or .tex. If you are providing a .tex file, please upload it under the item type u2018LaTeX Source Fileu2019 and leave your .pdf version as the item type u2018Manuscriptu2019.

2) Some material included in your submission may be copyrighted. According to PLOSu2019s copyright policy, authors who use figures or other material (e.g., graphics, clipart, maps) from another author or copyright holder must demonstrate or obtain permission to publish this material under the Creative Commons Attribution 4.0 International (CC BY 4.0) License used by PLOS journals. Please closely review the details of PLOSu2019s copyright requirements here: PLOS Licenses and Copyright. If you need to request permissions from a copyright holder, you may use PLOS's Copyright Content Permission form.

Please respond directly to this email and provide any known details concerning your material's license terms and permissions required for reuse, even if you have not yet obtained copyright permissions or are unsure of your material's copyright compatibility. Once you have responded and addressed all other outstanding technical requirements, you may resubmit your manuscript within Editorial Manager.

Potential Copyright Issues:

i) Figures 7, and 9. Please confirm whether you drew the images / clip-art within the figure panels by hand. If you did not draw the images, please provide (a) a link to the source of the images or icons and their license / terms of use; or (b) written permission from the copyright holder to publish the images or icons under our CC BY 4.0 license. Alternatively, you may replace the images with open source alternatives. See these open source resources you may use to replace images / clip-art:

- https://commons.wikimedia.org

- https://openclipart.org/.

3) Please amend your detailed Financial Disclosure statement. This is published with the article. It must therefore be completed in full sentences and contain the exact wording you wish to be published.

1) State what role the funders took in the study. If the funders had no role in your study, please state: "The funders had no role in study design, data collection and analysis, decision to publish, or preparation of the manuscript."

2) If any authors received a salary from any of your funders, please state which authors and which funders.

4) We note that your Data Availability Statement in the online submission form is currently as follows: "Yes - all data are fully available without restriction." Please provide a complete Data Availability Statement in the submission form, ensuring you include all necessary access information. If your research concerns only data provided within your submission, please write "All data are in the manuscript and/or supporting information files" as your Data Availability Statement. If your research concerns data from external sources, please amend your Data Availability Statement to include the full link to the data.

Note: If the reviewer comments include a recommendation to cite specific previously published works, please review and evaluate these publications to determine whether they are relevant and should be cited. There is no requirement to cite these works unless the editor has indicated otherwise.

**Reviewers' comments:**

Reviewer's Responses to Questions

Reviewer #1: Obama et al., present a novel statistical method for inferring the multiplicity of infection MOI and haplotype frequencies of pathogens within individuals from genetic data. The method addresses an important problem, as MOI can significantly complicate efforts to model the transmission, persistence and prevalence of infectious diseases.

Due to time constraints, I have not been able to go through all the derivations provided in full-detail. However, I have checked that the associated code implementing the methods runs as described. While I believe that this method provides a valuable addition to the literature, its limitations could be better described and I have outlined a number of issues that need to be addressed prior to publication.

Major issues:

- The authors focus primarily on data that considers only a very small number of loci (<100 markers). While this is certainly useful, many modern genomic studies of major human pathogens including Malaria typically consider many thousands of loci. For example, the MalariaGen project has whole genome sequencing from over 20,000 Plasmodium falciparum samples. It is important that the authors more clearly demonstrate the computational limitations of their approach. This could easily be done by generating a plot of the compute time versus dataset complexity in both the number of loci and number of samples. The authors should also describe some of the literature that attempts to make use of the large number of loci to more accurately estimate MOI and phase genotypes using WGS data. For example, methods such as DEploid (Zhu et al., 1017) and coiaf (Paschalidis et al., 2023) can consider many thousands of SNPs in a reasonable amount of time.

- The manuscript is currently quite hard to follow as many of the parameters are defined in the methods but not in the main text where they are first introduced (i.e lines 197-200). While I appreciate that including all derivations in the results would be tedious, the parameters should all be defined where they are first stated.

- The implementation of the model currently estimates unrealistically low haplotype frequencies. For example, line 29 of the ‘MOI-tutorial.R’ code outputs many (~50%) haplotypes with frequencies well below the limit of detection (0.01) given the example only contains 100 samples. The tendency of the approach to assign unrealistic frequencies to certain haplotypes needs to be discussed carefully in the text and perhaps a warning could be associated with results such as these that are unlikely to be accurate. Ideally, the method would include an approach to shrink these frequencies to zero perhaps using a regularisation penalty.

- Identifying SNPs and microsatellites is an error prone process. It is important that the authors consider what impact errors in the input data have on the results of the method. A plot indicating the impact of different levels of error in the input data would be very useful to wider readers.

Minor issues:

- The authors refer to the method being applicable to ‘arbitrary genetic architecture’ in a number of places. It is not clear what ‘arbitrary’ means in this context. For example, the method is not applicable to loci that are not consistently present in all genomes of a species. I think this statement should be replaced by a more specific one.

- The summary of alternative methods (lines 94-107) should be made slightly more detailed. Currently, it requires the reader to look up many of the references provided to understand what the authors are referring to (i.e I don’t automatically have a good intuition for what ‘ad-hoc methods’ are). It would be great if the ‘ad-hoc’ methods and the first statistical model (ref. 2) are explained with an additional sentence or two.

- Line 74: “Co-transmitted” would normally mean the transmission of multiple genotypes within a single transmission event. This does not match up with the definition of MOI which is correctly and nicely stated later on.

- Some of the figures have quite low resolution which should be addressed.

Reviewer #2: Tsoungui Obama and Schneider propose a method to estimate haplotype frequencies, haplotype prevalences and the multiplicity of infection based on unphased data from multiple multiallelic markers. The key advance relative to the authors’ previous work (references [10] and [12]) appears to be a generalisation to the case of multiple multiallelic markers. An expectation-maximisation algorithm is derived to compute maximum likelihood estimates for haplotype frequencies and the multiplicity of infection, which are then plugged into derived formulae for haplotype prevalences. Asymptotic variances and finite sample properties of these estimators are considered. The practical utility of this method in the context of malaria genomic epidemiology is illustrated through an analysis of markers associated with drug resistance in Plasmodium falciparum.

Major comments

• It would be useful if the authors could articulate more clearly the advance represented by the current manuscript, relative to the references [9], [10], [12], [22] and [25], particularly in the introduction and discussion. For instance, in the section “Deriving the EM-algorithm” of the Mathematical Appendix, the authors state that their “derivation generalizes the ones in [1] for n biallelic loci and [2] for two multiallelic loci to the case of n multiallelic loci. Namely, it turns out that the structure of the derivations is almost identical and only needs slight modifications”; this parallel, however, does not seem to be mentioned in the main text.

• To improve the readability of the main text, perhaps the authors could consider introducing a table of notation? The equations stated in the main text results, with forward-reference to equations in the methods, are difficult to interpret. For instance, the PGF G first appears in Equation (1b) but is defined later on in the text.

• The authors frame the statistical model with respect to the notion of superinfection vs co-transmission. However, my understanding is that the statistical model is more simply predicated on the assumption of a Poisson-distributed number of unrelated/independent clones within a sample. I wonder if it might be clearer to introduce the model with respect to the latter notion, and then link this back to a more ‘mechanistic’ notion of superinfection? To aid readability, perhaps the authors could include a self-contained summary or list of model assumptions?

Minor comments

• Perhaps the authors could provide several examples of pathogens or organisms, beyond falciparum malaria, to which this method could be applied? Perhaps the authors could also comment on the interpretation of superinfection if this method was applied to vivax malaria, especially given the inoculation of multiple hypnozoites per mosquito bite and related relapses?

• Some of the terminology used by the authors is ambiguous. For instance, the term “general genetic architecture” is defined to refer to “multiple multiallelic markers” on lines 590 and 647, but is used earlier in the introduction without definition. Rather than referring to “more complicated underlying genetic architectures” or “simple genetic architectures” (line 118), it would be useful to explicitly specify the case in consideration.

• In Table 2, why are there MOI estimates < 1if the method is tailored to disease-positive specimens?

• Line 783: should “beta distribution” be replaced with “gamma distribution”?

• In the Introduction (line 102), the authors mention that references [13] and [14] also handle multiple multiallelic markers; given the generalisation of the authors’ previous work to the case of multiple multiallelic markers appears to be a key advance, a brief explanation of the key difference between references [13] and [14], and the present model, would be useful.

**********

**Have the authors made all data and (if applicable) computational code underlying the findings in their manuscript fully available?**

The PLOS Data policy requires authors to make all data and code underlying the findings described in their manuscript fully available without restriction, with rare exception (please refer to the Data Availability Statement in the manuscript PDF file). The data and code should be provided as part of the manuscript or its supporting information, or deposited to a public repository. For example, in addition to summary statistics, the data points behind means, medians and variance measures should be available. If there are restrictions on publicly sharing data or code —e.g. participant privacy or use of data from a third party—those must be specified.

Reviewer #1: Yes

Reviewer #2: Yes

**********

PLOS authors have the option to publish the peer review history of their article (what does this mean?). If published, this will include your full peer review and any attached files.

If you choose “no”, your identity will remain anonymous but your review may still be made public.

**Do you want your identity to be public for this peer review?**  For information about this choice, including consent withdrawal, please see our Privacy Policy.

Reviewer #1: No

Reviewer #2: No

[NOTE: If reviewer comments were submitted as an attachment file, they will be attached to this email and accessible via the submission site. Please log into your account, locate the manuscript record, and check for the action link "View Attachments". If this link does not appear, there are no attachment files.]

**Figure resubmission:**

While revising your submission, please upload your figure files to the Preflight Analysis and Conversion Engine (PACE) digital diagnostic tool, https://pacev2.apexcovantage.com/. PACE helps ensure that figures meet PLOS requirements. To use PACE, you must first register as a user. Registration is free. Then, login and navigate to the UPLOAD tab, where you will find detailed instructions on how to use the tool. If you encounter any issues or have any questions when using PACE, please email PLOS at figures@plos.org. Please note that Supporting Information files do not need this step. If there are other versions of figure files still present in your submission file inventory at resubmission, please replace them with the PACE-processed versions.

**Reproducibility:**

?>

# Author comment: Author response to Decision Letter 1

**DOI:** 10.1371/journal.pcbi.1012955.r002

11 Nov 2025

Attachment Submitted filename: Response to reviewers.pdf

# Peer review: Decision Letter 1

**DOI:** 10.1371/journal.pcbi.1012955.r003

**Authors:** James McCaw, Jennifer Flegg

22 Feb 2026

PCOMPBIOL-D-25-00481R1

Molecular surveillance of multiplicity of infection, haplotype frequencies, and prevalence in infectious diseases

PLOS Computational Biology

Dear Dr. Tsoungui Obama,

Thank you for submitting your manuscript to PLOS Computational Biology.

I apologise for the time that your revision was out for review, but it coincided with the extended summer holiday period in the southern hemisphere and both reviewers and I (as editor) were away for some time.

Both (original) reviewers and I agree that you have substantially improved the manuscript, and have just a few additional queries and minor requirements for you to address.

Please note one of the reviewer's comments were provided to me from outside of the formal PCB system so I have pasted them in to this email by hand. Please address them as usual.

Below is the remainder of the standard PCB form-letter and reviewer's comments.

James

After careful consideration, we feel that it has merit but does not fully meet PLOS Computational Biology's publication criteria as it currently stands. Therefore, we invite you to submit a revised version of the manuscript that addresses the points raised during the review process.

Please submit your revised manuscript by Apr 24 2026 11:59PM. If you will need more time than this to complete your revisions, please reply to this message or contact the journal office at ploscompbiol@plos.org. When you're ready to submit your revision, log on to https://www.editorialmanager.com/pcompbiol/ and select the 'Submissions Needing Revision' folder to locate your manuscript file.

Please include the following items when submitting your revised manuscript:

* A letter that responds to each point raised by the editor and reviewer(s). You should upload this letter as a separate file labeled 'Response to Reviewers'. This file does not need to include responses to formatting updates and technical items listed in the 'Journal Requirements' section below.

* A marked-up copy of your manuscript that highlights changes made to the original version. You should upload this as a separate file labeled 'Revised Manuscript with Track Changes'.

* An unmarked version of your revised paper without tracked changes. You should upload this as a separate file labeled 'Manuscript'.

If you would like to make changes to your financial disclosure, competing interests statement, or data availability statement, please make these updates within the submission form at the time of resubmission. Guidelines for resubmitting your figure files are available below the reviewer comments at the end of this letter.

We look forward to receiving your revised manuscript.

Kind regards,

James M McCaw, PhD

Academic Editor

PLOS Computational Biology

Jennifer Flegg

Section Editor

PLOS Computational Biology

**Journal Requirements:**

If the reviewer comments include a recommendation to cite specific previously published works, please review and evaluate these publications to determine whether they are relevant and should be cited. There is no requirement to cite these works unless the editor has indicated otherwise.

**Reviewers' comments:**

Reviewer's Responses to Questions

**Comments to the Authors:**

**Please note here if the review is uploaded as an attachment.**

Reviewer #1: The authors have addressed most of my concerns, and I appreciate the addition of the new benchmarking section. While CPU time is now reported, several references are made to the algorithm running out of memory. The memory usage of the algorithm should also be stated, or at the very least the maximum amount of memory available during benchmarking should be clearly specified so that readers can properly interpret what “memory overflow” refers to.

Reviewer #2: The authors have largely addressed my previous comments. The readability of the revised manuscript is much improved with respect to notation, and the method is better contextualised in the literature.

I appreciate the authors’ point that the manuscript is largely targeted to a biological audience. However, I would still argue that a concise, self-contained list of key model assumptions and rationales/limitations could be practically useful for practitioners to decide whether the present model is suited to their particular context (e.g. the number of markers, the expected degree of genotyping error and the associated error structure, genotype missingness, whether markers are relatively fast or slow-evolving, etc), and to be aware of potential biases or artefacts when interpreting the model outputs (e.g. due to outliers, imputation or limited sample sizes). Given the model assumptions are largely motivated in the context of falciparum malaria, this could be particularly useful for broader applications to other diseases.

Perhaps the authors could also comment on any biases that might arise if this method (predicated on the assumption of independent pathogen variants) is applied to data with significant relatedness between pathogen variants (e.g. due to significant co transmission as characterised by Wong et al [doi: 10.1186/s13073-017-0398-0] or related relapses of vivax malaria)? Perhaps the authors could also expand on lines 595-596 to discuss possible biases in MOI estimates due to selection?

Minor comment: it might be worth including SH in Table 1.

**********

**Have the authors made all data and (if applicable) computational code underlying the findings in their manuscript fully available?**

The PLOS Data policy requires authors to make all data and code underlying the findings described in their manuscript fully available without restriction, with rare exception (please refer to the Data Availability Statement in the manuscript PDF file). The data and code should be provided as part of the manuscript or its supporting information, or deposited to a public repository. For example, in addition to summary statistics, the data points behind means, medians and variance measures should be available. If there are restrictions on publicly sharing data or code —e.g. participant privacy or use of data from a third party—those must be specified.

Reviewer #1: Yes

**********

PLOS authors have the option to publish the peer review history of their article (what does this mean?). If published, this will include your full peer review and any attached files.

If you choose “no”, your identity will remain anonymous but your review may still be made public.

**Do you want your identity to be public for this peer review?**  For information about this choice, including consent withdrawal, please see our Privacy Policy.

Reviewer #1: No

**Figure resubmission:**

After uploading your figures to PLOS’s NAAS tool - https://ngplosjournals.pagemajik.ai/artanalysis, NAAS will process the files provided and display the results in the "Uploaded Files" section of the page as the processing is complete. If the uploaded figures meet our requirements (or NAAS is able to fix the files to meet our requirements), the figure will be marked as "fixed" above. If NAAS is unable to fix the files, a red "failed" label will appear above. When NAAS has confirmed that the figure files meet our requirements, please download the file via the download option, and include these NAAS processed figure files when submitting your revised manuscript.

**Reproducibility:**

To enhance the reproducibility of your results, we recommend that authors of applicable studies deposit laboratory protocols in protocols.io, where a protocol can be assigned its own identifier (DOI) such that it can be cited independently in the future. Additionally, PLOS ONE offers an option to publish peer-reviewed clinical study protocols. Read more information on sharing protocols at https://plos.org/protocols?utm_medium=editorial-email&utm_source=authorletters&utm_campaign=protocols

# Author comment: Author response to Decision Letter 2

**DOI:** 10.1371/journal.pcbi.1012955.r004

10 Mar 2026

Attachment Submitted filename: Response_to_reviewers.pdf

# Editor report: Decision Letter 2

**DOI:** 10.1371/journal.pcbi.1012955.r005

**Authors:** James McCaw, Jennifer Flegg

11 Mar 2026

Dear Dr. Tsoungui Obama,

We are pleased to inform you that your manuscript 'Molecular surveillance of multiplicity of infection, haplotype frequencies, and prevalence in infectious diseases' has been provisionally accepted for publication in PLOS Computational Biology.

Before your manuscript can be formally accepted you will need to complete some formatting changes, which you will receive in a follow up email. A member of our team will be in touch with a set of requests.

Please note that your manuscript will not be scheduled for publication until you have made the required changes, so a swift response is appreciated.

IMPORTANT: The editorial review process is now complete. PLOS will only permit corrections to spelling, formatting or significant scientific errors from this point onwards. Requests for major changes, or any which affect the scientific understanding of your work, will cause delays to the publication date of your manuscript.

Should you, your institution's press office or the journal office choose to press release your paper, you will automatically be opted out of early publication. We ask that you notify us now if you or your institution is planning to press release the article. All press must be co-ordinated with PLOS.

Thank you again for supporting Open Access publishing; we are looking forward to publishing your work in PLOS Computational Biology.

Best regards,

James M McCaw, PhD

Academic Editor

PLOS Computational Biology

Jennifer Flegg

Section Editor

PLOS Computational Biology

***********************************************************

# Editor report: Acceptance letter

**DOI:** 10.1371/journal.pcbi.1012955.r006

**Authors:** James McCaw, Jennifer Flegg

PCOMPBIOL-D-25-00481R2

Molecular surveillance of multiplicity of infection, haplotype frequencies, and prevalence in infectious diseases

Dear Dr Tsoungui Obama,

I am pleased to inform you that your manuscript has been formally accepted for publication in PLOS Computational Biology. Your manuscript is now with our production department and you will be notified of the publication date in due course.

The corresponding author will soon be receiving a typeset proof for review, to ensure errors have not been introduced during production. Please review the PDF proof of your manuscript carefully, as this is the last chance to correct any errors. Please note that major changes, or those which affect the scientific understanding of the work, will likely cause delays to the publication date of your manuscript.

Soon after your final files are uploaded, unless you have opted out, the early version of your manuscript will be published online. The date of the early version will be your article's publication date. The final article will be published to the same URL, and all versions of the paper will be accessible to readers.

For Research, Software, and Methods articles, you will receive an invoice from PLOS for your publication fee after your manuscript has reached the completed accept phase. If you receive an email requesting payment before acceptance or for any other service, this may be a phishing scheme. Learn how to identify phishing emails and protect your accounts at https://explore.plos.org/phishing.

Thank you again for supporting PLOS Computational Biology and open-access publishing. We are looking forward to publishing your work!

With kind regards,

Anita Estes

PLOS Computational Biology | Carlyle House, Carlyle Road, Cambridge CB4 3DN | United Kingdom ploscompbiol@plos.org | Phone +44 (0) 1223-442824 | ploscompbiol.org | @PLOSCompBiol
